# Quartz-Seq: a highly reproducible and sensitive single-cell RNA sequencing method, reveals non-genetic gene-expression heterogeneity

**DOI:** 10.1186/gb-2013-14-4-r31

**Published:** 2013-04-17

**Authors:** Yohei Sasagawa, Itoshi Nikaido, Tetsutaro Hayashi, Hiroki Danno, Kenichiro D Uno, Takeshi Imai, Hiroki R Ueda

**Affiliations:** 1 Functional Genomics Unit, RIKEN Center for Developmental Biology, 2-2-3 Minatojima-minamimachi, Chuo-ku, Kobe, Hyogo 650-0047, Japan; 2 Genome Resource and Analysis Unit, RIKEN Center for Developmental Biology, 2-2-3 Minatojima-minamimachi, Chuo-ku, Kobe, Hyogo 650-0047, Japan; 3 Laboratory for Systems biology, RIKEN Center for Developmental Biology, 2-2-3 Minatojima-minamimachi, Chuo-ku, Kobe, Hyogo 650-0047, Japan; 4 Laboratory for Sensory Circuit Formation, RIKEN Center for Developmental Biology, 2-2-3 Minatojima-minamimachi, Chuo-ku, Kobe, Hyogo 650-0047, Japan; 5 JST, PRESTO, 2-2-3 Minatojima-minamimachi, Chuo-ku, Kobe, Hyogo 650-0047, Japan; 6 Laboratory for Synthetic Biology, Quantitative Biology Center, RIKEN, 2-2-3 Minatojima-minamimachi, Chuo-ku, Kobe, Hyogo 650-0047, Japan; 7 Bioinformatics Research Unit, Advanced Center for Computing and Communication, RIKEN, 2-1 Hirosawa, Wako, Saitama 351-0198, Japan

**Keywords:** Single cell, RNA-seq, Transcriptome, Sequencing, Bioinformatics, Cellular heterogeneity, Cell biology

## Abstract

Development of a highly reproducible and sensitive single-cell RNA sequencing (RNA-seq) method would facilitate the understanding of the biological roles and underlying mechanisms of non-genetic cellular heterogeneity. In this study, we report a novel single-cell RNA-seq method called Quartz-Seq that has a simpler protocol and higher reproducibility and sensitivity than existing methods. We show that single-cell Quartz-Seq can quantitatively detect various kinds of non-genetic cellular heterogeneity, and can detect different cell types and different cell-cycle phases of a single cell type. Moreover, this method can comprehensively reveal gene-expression heterogeneity between single cells of the same cell type in the same cell-cycle phase.

## Background

Non-genetic cellular heterogeneity at the mRNA and protein levels has been observed within cell populations in diverse developmental processes and physiological conditions [1-4]. However, the comprehensive and quantitative analysis of this cellular heterogeneity and its changes in response to perturbations has been extremely challenging. Recently, several researchers reported quantification of gene-expression heterogeneity within genetically identical cell populations, and elucidation of its biological roles and underlying mechanisms [5-8]. Although gene-expression heterogeneities have been quantitatively measured for several target genes using single-molecule imaging or single-cell quantitative (q)PCR, comprehensive studies on the quantification of gene-expression heterogeneity are limited [9] and thus further work is required. Because global gene-expression heterogeneity may provide biological information (for example, on cell fate, culture environment, and drug response), the question of how to comprehensively and quantitatively detect the heterogeneity of mRNA expression in single cells and how to extract biological information from those data remains to be addressed.

Single-cell RNA sequencing (RNA-seq) analysis has been shown to be an effective approach for the comprehensive quantification of gene-expression heterogeneity that reflects the cellular heterogeneity at the single-cell level [10,11]. To understand the biological roles and underlying mechanisms of such heterogeneity, an ideal single-cell transcriptome analysis method would provide a simple, highly reproducible, and sensitive method for measuring the gene-expression heterogeneity of cell populations. In addition, this method should be able to distinguish clearly the gene-expression heterogeneity from experimental errors.

Single-cell transcriptome analyses, which can be achieved through the use of various platforms, such as microarrays, massively parallel sequencers and bead arrays [12-17], are able to identify cell-type markers and/or rare cell types in tissues. These platforms require nanogram quantities of DNA as the starting material. However, a typical single cell has approximately 10 pg of total RNA and often contains only 0.1 pg of polyadenylated RNA, hence, o obtain the amount of DNA starting material that is required by these platforms, it is necessary to perform whole-transcript amplification (WTA).

Previous WTA methods for single cells fall into two categories, based on the modifications that are introduced into the first-strand cDNAs in the PCR-based methods. One approach is based on the poly-A tailing reaction, and the other on the template-switching reaction. In principle, the goal of poly-A tailing is to obtain both full-length first-strand cDNAs and truncated cDNAs. The aim of template switching is to obtain first-strand cDNAs that have reached the 5' ends of the RNA templates. These modified cDNAs are amplifiable by subsequent PCR enrichment methods.

Kurimoto *et al*. reported a quantitative WTA method based on the poly-A-tailing reaction for single-cell microarrays [12]. They used this single-cell transcriptome analysis, and published initial validation data for technical replicates, each of which required 10 pg of total RNA. The Pearson correlation coefficient (PCC) for the reproducibility of this method using 10 pg of total RNA per reaction was approximately 0.85 [12]. Using a method similar to the one used by Kurimoto *et al*., Tang *et al*. performed single-cell RNA-seq. When they applied their method to a single mouse oocyte (around 1 ng of total RNA), these researchers were able to detect a larger number of genes than could be identified using a microarray approach [13]. However, these methods are complicated because they require multiple PCR tubes for a single cell, and gel purification is required for the removal of unexpected byproducts [18,19]. Furthermore, detailed quantitative analysis of the performance of the Tang *et al*. single-cell RNA-seq method, including its reproducibility and sensitivity, has not been analyzed.

Two single-cell RNA-seq methods based on the template-switching reaction have been reported. Islam *et al*. described a method called single-cell tagged reverse transcription sequencing (STRT-seq), which is a highly multiplexed single-cell RNA-seq method that can detect the restricted 5' ends of mRNAs [14]. Ramsköld *et al*. developed Smart-Seq (the WTA part of Smart-Seq is now marketed as SMARTer Ultra Low RNA Kit for Illumina Sequencing, Clontech, Mountain View, CA, USA), which exhibits a greater read coverage across transcripts than previously developed methods [16]. The PCCs for the reproducibility of the methods using 10 pg of total RNA were both approximately 0.7. Recently, Hashimshony *et al*. described CEL-Seq (Cell Expression by Linear amplification and Sequencing), which is an *in vitro *transcription (IVT)-based method but not a PCR-based method. CEL-Seq is a highly multiplexed single-cell RNA-seq method that can detect the 3' end of mRNA [17]. CEL-Seq was shown to detect significantly more genes in single mouse embryonic stem (ES) cells compared with STRT-Seq. The performance of these reported methods is sufficient for the identification of cell-type markers. However, their specifications for WTA did not validate whether the methods are sufficient to quantitatively assess the global gene-expression heterogeneity that is indicative of cellular heterogeneity. Because the PCC for reproducibility is greater than 0.95 for conventional non-WTA RNA-seq, it would be desirable to improve the reproducibility and sensitivity of single-cell RNA-seq to a greater degree than is possible with existing methods.

To comprehensively and quantitatively detect gene-expression heterogeneity, we have developed a simple and highly quantitative single-cell RNA-seq approach that we term Quartz-Seq. In this study, we identified some defective factors that allowed us to simplify the experimental procedures and improve the quantitative performance. In particular, to maintain the simplicity and enhance the quantitative performance of WTA, we improved three crucial aspects: 1) we achieved robust suppression of byproduct synthesis; 2) we identified a robust PCR enzyme that allows the use of a single-tube reaction; and 3) we determined the optimal conditions of reverse transcription (RT) and second-strand synthesis for the capturing mRNA and the first-strand cDNA. We also performed a quantitative comparison between our method and previously developed methods using 10 pg of total RNA as the starting material the reproducibility and sensitivity of the Quartz-Seq method was better than those of the other methods.

When used in the global expression analysis of real single cells, the single-cell Quartz-Seq approach successfully detected gene expression heterogeneity even between cells of the same cell type and in the same cell-cycle phase. This observed gene-expression heterogeneity was found to be highly reproducible in two independent experiments, and could be distinguished from experimental errors, which were measured through technical replicates of pooled samples. We also found that single-cell Quartz-Seq was able to discriminate more easily between different cell types and/or between different cell-cycle phases. Therefore, single-cell Quartz-Seq is a useful method for the comprehensive identification and quantitative assessment of cellular heterogeneity.

## Results

### Whole-transcript amplification for single-cell Quartz-Seq and Quartz-Chip

**Figure 1:**
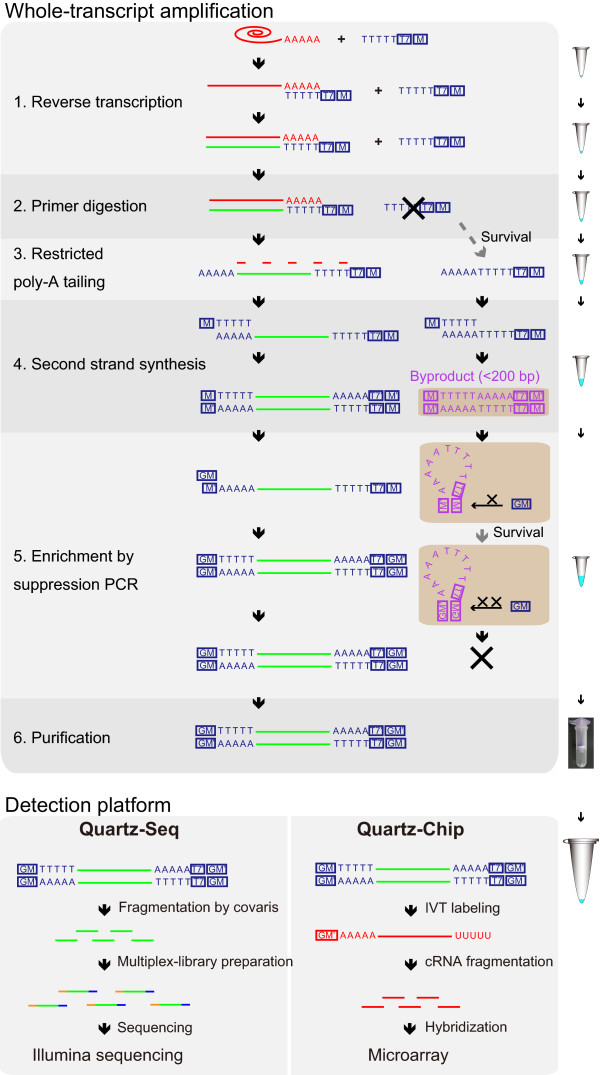
**Schematic of the single-cell Quartz-Seq and Quartz-Chip methods**. All of the steps of the whole-transcript amplification were executed in a single PCR tube. The first-strand cDNA was synthesized using the reverse transcription (RT) primer, which contains oligo-dT24, the T7 promoter (T7) and the PCR target region (M) sequences. After the first-strand synthesis, the majority of the RT primer was digested by exonuclease I, although it was not possible to eliminate the RT primer completely using this procedure. A poly-A tail was then added to the 3' ends of the first-strand cDNA and to any surviving RT primer. After the second-strand synthesis with the tagging primer, the resulting cDNA and the byproducts from the surviving primers contained the whole-transcript amplification (WTA) adaptor sequences, which include the RT primer sequence and the tagging primer sequence. These DNAs were used for the suppression PCR, which used the suppression PCR primer. Enrichment of the short DNA fragments, such as the byproducts, was suppressed. After the enrichment, the high-quality cDNA, which did not contain any byproducts, was obtained. The amplified cDNAs then had the T7 promoter sequence at the 3' ends of the DNA. These cDNAs were used for the Illumina sequencing and microarray experiments.

The WTA for Quartz-Seq and Quartz- consists of five main steps (Figure 1). The first step is a reverse transcription with an RT primer to generate the first-strand cDNAs from the target RNAs. The second step is a primer digestion with exonuclease I; this is one of the key steps to prevent the synthesis of byproducts. The third step is the addition of a poly-A tail to the 3' ends of the first-strand cDNAs, and the fourth step is the second-strand synthesis using a tagging primer, which prepares the substrate for subsequent amplification. The fifth step is a PCR enrichment reaction with a suppression PCR primer to ensure that a sufficient quantity of DNA is obtained for the massively parallel sequencers or microarrays. All five steps are completed in a single PCR tube without any purification. The amplified cDNA contains WTA adaptor sequences from the RT primer and the tagging primer.

The amplified cDNA was then used in a massively parallel sequencer (Quartz-Seq) and a microarray system (Quartz-Chip). For the Quartz-Seq, the amplified cDNA was fragmented using the Covaris shearing system. The fragmented cDNA was ligated to adaptors, which enable the multiplex production of paired-end (PE) sequences. The DNA sequencing library was analyzed using an Illumina sequencer. For the Quartz-Chip method, we synthesized labeled cRNA from the amplified cDNA using *in vitro *transcription. The labeled cRNA was used for the microarray analysis.

### Performance improvements of whole-transcript amplification

In previous WTA methods based on the poly-A-tailing reaction, excessive amounts of byproducts are produced (see Additional file 1, Figure S1). These byproducts (usually DNA < 200 bp in length) are derived from the RT primer. The RT primer is modified by terminal deoxynucleotidyl transferase, similarly to the first-strand cDNA. The modified RT primer then causes synthesis of the byproducts [18], and the amplified byproducts need to be removed by gel purification [18,19] (see Additional file 2, Supplementary note). This gel-purification step for the removal of these byproducts increases the complexity of the method. The byproducts contain WTA adaptor sequences. We found that the byproducts cause early saturation of the PCR amplification, and reduce the molar ratio between the objective cDNA and the byproducts. The contamination rate from the WTA adaptor sequence was dramatically increased using the Illumina sequencing method (see Additional file 1, Figure S2e,f).

To overcome this byproduct contamination, the byproduct synthesis was completely eliminated using a combination of exonuclease I treatment, restricted poly-A tailing, and an optimized suppression PCR (see Additional file 1, Figure S3 and Figure S4). We successfully eliminated the synthesis of byproducts using the following three improvement points, thus eliminating the need for gel purification.

The first improvement point is the adjustment of the RT primer concentration after the RT procedure described above. We used the minimum primer concentration for the RT. Moreover, we removed the RT primer by treating with exonuclease I, which digests single-strand DNAs such as primers. This exonuclease I digestion suppressed the synthesis of byproducts (see Additional file 1, Figure S2a). However, the primer removal was not complete at this point, which is in agreement with the results of a previous study [18] (see Additional file 1, Figure S1). The molar ratio between the single-cell-level mRNA (0.1 pg; Ensembl Mouse Transcript 1817 bp average size) and the RT primer is greater than 190,000. Complete removal solely by exonuclease I digestion was difficult. The remaining primers were then modified by terminal deoxynucleotidyl transferase, similarly to the first-strand cDNA; these modified primers caused the production of byproducts.

In the second improvement point, to prevent amplification of the modified primers, we used suppression PCR technology. Suppression PCR is very effective in the suppressing amplification of small-size DNA that contains complementary sequences at both ends of the template DNA [20]. In suppression PCR, these complementary sequences can bind to each other, and the self-bound template DNA forms a 'pan-like' structure. In addition, the DNA is not amplified by PCR because the PCR primer cannot bind to the template DNA (see Additional file 1, Figure S4). The target DNA size of suppression PCR depends on the end of the complementary sequences (including the length and GC content). We also identified a good primer sequence for the suppression PCR, and showed that the B-primer effectively suppressed the synthesis of byproducts (see Additional file 1, Figure S2c and Figure S4b).

In the third improvement point, to shorten the length of the remaining primers modified by the terminal transferase and thus make them targets for suppression PCR, we restricted the reaction time of the terminal transferase. This restriction suppressed the synthesis of byproducts (see Additional file 1, Figure S2b).

It should be noted that in addition, we found that topoisomerase V could suppress byproduct synthesis (see Additional file 1, Figure S2d). However, the mechanism of byproduct suppression by topoisomerase V is not known. Using the combination of the three previously discussed improvement points, we successfully suppressed the synthesis of byproducts completely in a single-tube reaction (see Additional file 1, Figure S2e), without using topoisomerase V.

Furthermore, we selected a robust PCR enzyme that was optimal for the single-tube reaction. The use of this DNA polymerase (MightyAmp DNA Polymerase (Takara Bio, Inc., Tokyo, Japan), also marketed as Terra PCR Direct Polymerase (Clontech, Mountain View, CA, USA)) improved the yield of cDNA (see Additional file 1, Figure S2c and Figure S5) and the reproducibility of the WTA replication (see Additional file 1, Figure S2d and Figure S5). In addition, by using this DNA polymerase (MightyAmp), the number of PCR cycles in WTA could be reduced.

Moreover, we improved the efficiencies of the RT and second-strand synthesis steps to counter the lowered reproducibility of the WTA that occurred as a result of the variable efficiencies of these steps. We identified the optimal annealing temperature that reduced the variability in the efficiencies of these steps (see Additional file 1, Figure S2a,d b). Our simplified method enabled us to consistently obtain highly reproducible cDNA that was optimized for RNA-seq (see Additional file 1, Figure S6).

### Reproducibility and sensitivity of single-cell Quartz-Seq

We performed single-cell Quartz-Seq with 10 pg of diluted total ES-cell RNA to validate the reproducibility of the technical replicates. We prepared a multiplex, PE DNA sequencing library from the amplified cDNA produced from the 10 pg of total RNA. The DNA sequencing library was analyzed using a massively parallel sequencer (HiSeq 1000/2000; Illumina).

**Figure 2:**
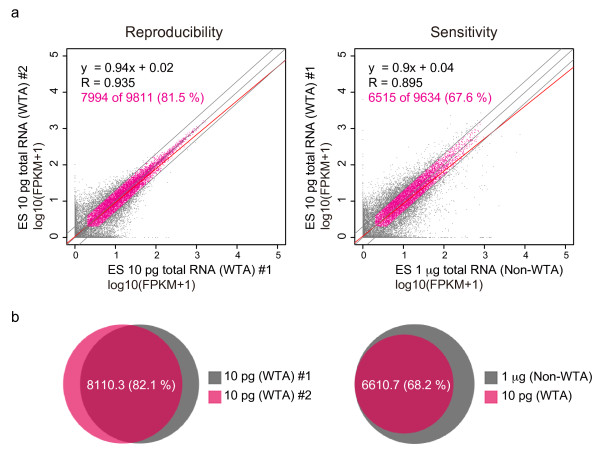
**Reproducibility and sensitivity of single-cell Quartz-Seq**. **(a) **Representative scatter plot of the gene-expression data from two replicate single-cell Quartz-Seq analyses of 10 pg of total embryonic stem (ES)-cell RNA (left panel). The blue line indicates a two-fold change, and the red line is a linear regression. Scatter plot of single-cell Quartz-Seq (whole-transcript amplification; WTA) and conventional RNA-seq (non-WTA) data using 1 µg of total ES-cell RNA (right panel). **(b) **Ratio of detected genes for three replicate Quartz-Seq analyses. Using two different independent Quartz-Seq experiments, 82.1% of the genes were detected by (left panel). The right panel shows the ratio of the genes detected by Quartz-Seq and conventional RNA-seq; more than 68.2% of the genes were detected by single-cell Quartz-Seq.

Pairwise comparisons of the products of triplicate amplifications were used to quantify the reproducibility of the protocol based on the PCCs (Figure 2a) in log_10_-transformed fragments per kilobase of transcript per million fragments sequenced (FKPM). The PCCs of these comparisons were approximately 0.93. We counted highly reproducible expressed transcripts with FPKM greater than 1.0 and that exhibited less than two-fold expression changes between technical replicates. The single-cell Quartz-Seq method was capable of reproducibly detecting a (mean ± SD) 8,110.3 ± 100.8 (82.1 ± 0.6%) of 9,872.6 ± 54.4 transcripts (Figure 2b; for pairwise plots, see Additional file 3, Figure S7, and for linear regression and correlation analysis, see Additional file 4, Table S1).

To evaluate the sensitivity of Quartz-Seq, we compared the results of conventional RNA-seq (non-WTA) and Quartz-Seq. The PCCs of these comparisons were approximately 0.89, Figure 2a). We also counted highly sensitive transcripts that had FPKM greater than 1 and exhibited less than two-fold expression changes between technical replicates. The single-cell Quartz-Seq method was capable of sensitively detecting 6,605 ± 139.9 (68.1 ± 0.5%) of 9,686 ± 63.7 transcripts (Figure 2b).

To evaluate the over-representation of the sequences derived from the WTA and the library preparation, we searched for the WTA adaptor sequence in all of the sequence reads using sequence similarity (see details in Materials and methods). Using Smart-Seq, 21.1 ± 3.05% of the sequences were identified as WTA adaptor (Smart-Seq, 10 pg ES-cell DNA, PE 30 million reads, *n *= 4; see Additional file 1, Figure S8) whereas 7.68 ± 0.66% of the sequences were identified as WTA adaptors by Quartz-Seq (Quartz-Seq, 10 pg ES-cell DNA, PE 60 million reads, *n *= 3; see Additional file 1, Figure S8).

We also evaluated the number of reads required for the method to detect mRNAs. From 0.01, 0.1, 0.5 1.0, 5.0, 10, 30, and 45 million reads (uniquely mapped reads, single-ended, 50 bp), we counted the number of detected genes and calculated the PCCs between different samples of the same origin (10 pg of total RNA). We found that a Quartz-Seq result from more than 1 million reads had a correlation of greater than 0.9 and detected more than 7,642 ± 40 transcripts (73 ± 0.19%) (see Additional file 1, Figure S9).

Furthermore, we compared the cDNA lengths resulting from the Quartz-Seq and conventional RNA-seq methods (see Additional file 1, Figure S10). The median of the read coverage across the expressed transcripts (FPKM ≥ 10) was 53.8% (705 bp) for Quartz-Seq compared with 84.8% (1,326 bp) using conventional RNA-seq.

### Comparison between Quartz-Seq and other methods

**Figure 3:**
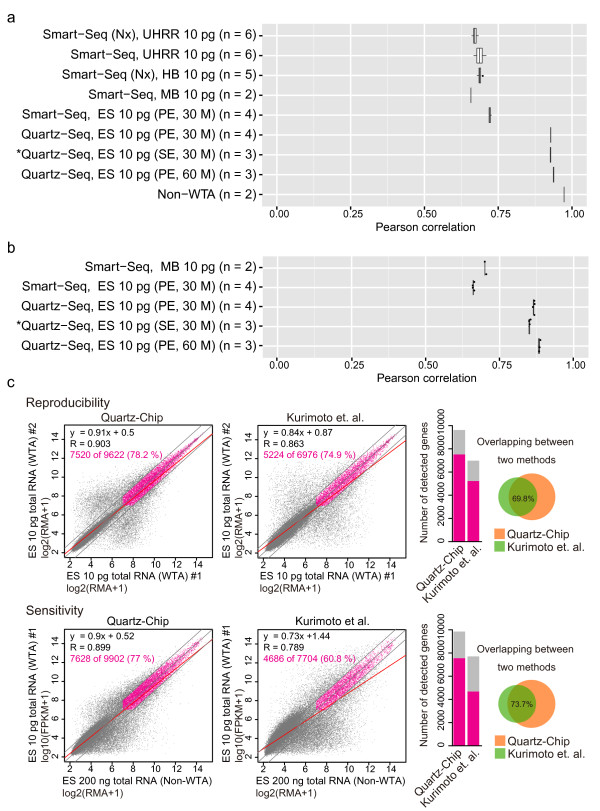
**Comparison of the performances of Quartz-Seq, Quartz-Chip and other methods**. **(a) **Box plot of Pearson correlation coefficients (PCCs) for the technical replication of Quartz-Seq and Smart-Seq with 10 pg diluted total RNA. We reanalyzed the following four original Smart-Seq datasets: mouse brain: MB; human brain (Nextera library preparation kit: HB (Nx); Universal human reference RNA: UHRR and UHRR with the Nextera library preparation kit: UHRR (Nx). The asterisk indicates the downsampling sequence reads from single-cell Quartz-Seq (paired-end (PE), 60 million reads, *n *= 3). **(b) **Box plot of PCCs between conventional RNA-seq and single-cell RNA-seq methods. **(c) **Comparison of our current and previous methods using 10 pg of total ES-cell RNA with GeneChip. Performance of (left) Quartz-Chip, and (right) the Kurimoto *et al*. method. The Kurimoto *et al*. data were reanalyzed using the original sets. The bar plots in the right panels show the numbers of genes that were detected with each method: the gray bars show the total number of genes, and the blue bars indicate the number of detected genes.

We carefully compared the quantitative performance of Quartz-Seq/Chip and three reported methods for single-cell transcriptome analysis. To compare the reproducibility of Quartz-Seq and Smart-Seq, four Smart-Seq data sets from the first Smart-Seq paper published by Ramsköld *et al*. were downloaded from the National Center for Biotechnology (NCBI) Gene Expression Omnibus (GEO) repository (GSE38495). We calculated the PCC between pairs of samples using 10 pg of total RNA (see Additional file 3, Figure S7; see Additional file 4, Table S1). The read numbers for the Quartz-Seq and Smart-Seq methods were adjusted to approximately 30 million reads of single-end sequences of 50 bp. The PCC values for Quartz-Seq and Smart-Seq were approximately 0.93 and 0.7, respectively (Figure 3a).

We counted highly reproducible transcripts that had FPKM or RPKM (reads per kilobase of exon model per million mapped) greater than 1 and exhibited less than two-fold expression changes between technical replicates. Quartz-Seq detected 8,110.3 ± 100 of 9,872.6 ± 54.4 transcripts (82.1 ± 0.6%), whereas Smart-Seq detected 2,745.5 ± 87.2 of 5,286.6 ± 92.0 transcripts (51.9 ± 0.8%) (human brain), 2,312 of 4,202 transcripts (55.0%) (mouse brain), and 3,706.0 ± 173.5 of 7,388.4 ± 256.2 transcripts (50.1 ± 1.5%) (universal human reference RNA). To confirm the experimental reproducibility, we performed additional Quartz-Seq and Smart-Seq procedures using 10 pg of diluted total RNA from an ES cell (*n *= 4). The read numbers for Quartz-Seq and Smart-Seq were adjusted to approximately 30 million reads of PE sequences of 50 bp. In this comparison, the PCC of Quartz-Seq was approximately 0.93, whereas the PCC of Smart-Seq was approximately 0.72 (Figure 3a). Quartz-Seq detected 7,739 ± 38.5 of 9,506.1 ± 22.7 transcripts (81.4 ± 0.5%), whereas Smart-Seq detected 2,320.6 ± 34.7 of 3675 ± 100.6 transcripts (63.1 ± 1.3%.

To evaluate the sensitivity of Quartz-Seq and Smart-Seq, we compared the results of conventional RNA-seq (non-WTA) and each method from same pooled total RNA mixture. The PCCs of these comparisons were approximately 0.88 for Quartz-Seq and 0.7 for Smart-Seq (Figure 3b). We also counted highly sensitive transcripts that had FPKM greater than 1 and exhibited less than two-fold expression changes between technical replicates. Using 10 pg ES total RNA, Smart-Seq was capable of detecting 2,906 ± 87.4 of 6,263 ± 149.1 transcripts (46.3 ± 0.5%) whereas Quartz-Seq detected 6,191.2 ± 58.0 of 9,342 ± 134.7 transcripts (66.2 ± 0.6%).

Next, we compared the quantitative performance between Quartz-Seq and CEL-Seq. CEL-Seq data sets from the original paper published by Hashimshony *et al*. were downloaded from the NCBI Sequence Read Archive (SRA) (SRP014672). We calculated the PCC between pairs of samples using 10 pg of *C. elegans *total RNA (see Additional file 3, Figure S7; see Additional file 4, Table S1). The PCCs of these comparisons were approximately 0.72. We counted highly reproducible expressed transcripts that had greater than 1.0 tags per million (tpm) and exhibited less than two-fold expression changes between technical replicates. CEL-Seq was capable of reproducibly detecting 2,564 ± 183.8 of 5,196.8 ± 364.9 transcripts (49.3 ± 1.7%). Moreover, we reanalyzed CEL-Seq data with mouse ES cell. We counted highly reproducible expressed transcripts that were greater than 1.0 tpm using the data. CEL-Seq detected 4,070.3 ± 332.4 transcripts in single mouse ES cells (*n *= 9), whereas Quartz-Seq detected 6,069.1 ± 854.9 transcripts in the same cell type (*n *= 35).

Subsequently, we compared the quantitative performances of our method and other methods based on the poly-A-tailing reaction. The detailed quantitative performance of the single-cell RNA-seq method of Tang *et al*. using 10 pg total RNA has not been analyzed. Therefore, we evaluated the performance (reproducibility and sensitivity) of the Quartz-Chip and Kurimoto *et al*. methods using a chip array (GeneChip; Affymetrix Inc., Santa Clara, CA, USA) with 10 pg of diluted total RNA. For this comparison, we reanalyzed the original data from Kurimoto *et al*., and compared the results with the Quartz-Chip data. We first compared the technical duplicates to quantify the reproducibility of both protocols (Figure 3c, upper panels). In this analysis, we counted a transcript that had robust multi-array averaging (RMA) expression greater than 7.0 and exhibited less than two-fold expression change between technical duplicates. The Quartz-Chip method was capable of reproducibly detecting 7,520 of 9,622 transcripts (78.2%) in the technical duplicates; however, the Kurimoto *et al*. method detected 5,224 of 6,976 transcripts (74.9%). In addition, 69.8% of the transcripts detected by the Kurimoto method were detected by Quartz-Chip.

We then compared the results of conventional non-WTA GeneChip with the Quartz-Chip and the Kurimoto *et al*. methods to quantify the sensitivity of both protocols (Figure 3c, lower panels). Similarly to the reproducibility analysis, we counted a transcript that had RMA expression greater than 7.0 and exhibited less than two-fold expression changes between the non-WTA GeneChip and the WTA samples (either the Quartz-Chip or the Kurimoto *et al*. methods). The Quartz-Chip method was capable of sensitively detecting 7,557 of 9,849 transcripts (77%), whereas the Kurimoto *et al*. method detected 4,686 of 7,704 transcripts (60.8%). In addition, 73.7% of the transcripts detected by the Kurimoto *et al*. method were also detected by Quartz-Chip.

### Limitations of Quartz-Seq

We investigated whether there are specific transcript structures that have greater noise or are under-represented. We calculated and compared the GC content and cDNA lengths of the amplified and unamplified isoforms obtained by each single-cell RNA-seq method (see Additional file 1, Figure S11). As expected, we found that the unamplified isoforms from Quartz-Seq had a higher GC content (mean 52.1%) than the amplified isoforms (mean 50.2%). In addition, the unamplified isoforms from Quartz-Seq had a higher GC content (mean 52.1%) than those from Smart-Seq (mean 51.5%). In the analysis of cDNA lengths, we found that the unamplified isoforms from Quartz-Seq had a shorter cDNA length (mean 1,684.0 bp) than the amplified (or detected) isoforms (mean 2558.6 bp).

We then represented the detailed comparison of technical noise between different polymerases. We performed Quartz-Seq with Ex Taq DNA polymerase (TaKaRa) instead of MightyAmp DNA polymerase because Ex Taq DNA polymerase has been used in previous methods (Kurimoto *et al*. and of Tang *et al*.) that were based on the poly-A-tailing reaction. We calculated the GC contents and cDNA lengths of the amplified and unamplified isoforms (see Additional file 1, Figure S11). Using Quartz-Seq, the unamplified isoforms produced by MightyAmp had a higher GC content (mean 52.1%) than those produced by Ex Taq (mean 51.7%), while the unamplified isoforms produced by MightyAmp had a shorter cDNA length (mean 1,684.0 bp) than those produced by Ex Taq (mean 2,481.1 bp).

### Single-cell Quartz-Seq detects different cell types

Heterogeneous cell populations, such as cultured cell lines and tissues, are composed of various types of single cells, which have different gene-expression patterns. We therefore tested whether single-cell Quartz-Seq can distinguish between different cell types and whether it can detect the differentially expressed genes that are characteristic of each cell type. We performed single-cell Quartz-Seq with 12 mouse ES cells and with 12 primitive endoderm (PrE) cells that are directly differentiated from ES cells [21]. We collected single cells directly into PCR tubes using fluorescence-activated cell sorting (FACS), as previously reported [22]. In this system, all sorted cells were readily discerned as single cells (see Additional file 1, Figure S13). We successfully obtained amplification products from almost all of these single cells (98%, *n *= 50, see Additional file 1, Figure S13). The ES and PrE cells were collected during the G1 phase of the cell cycle (see Additional file 1, Figure S12). Although the cell population that combined cells from all cell-cycle phases contained an average of approximately 10 pg of total RNA per cell, the cells in G1 phase contained only approximately 6 pg of total RNA per single cell (see Additional file 1, Figure S14).

**Figure 4:**
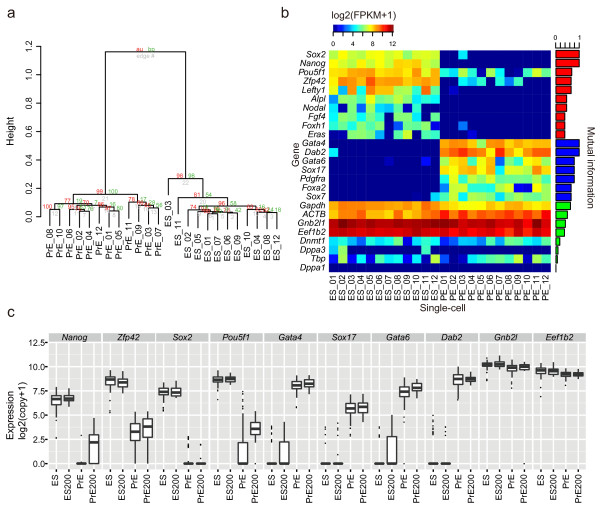
**Single-cell Quartz-Seq detects different cell types**. Results of single-cell Quartz-Seq with 12 embryonic stem (ES) cells and 12 primitive endoderm (PrE) cells. **(a) **Clustering of all samples. **(b) **Heat map of the marker genes for ES and PrE cells and the housekeeping genes. The bar plot in the right panel shows the mutual information (MI); a high degree of MI indicates high differential expression between two cell states. **(c) **Verification of the expression pattern between single cells using amplification-free single-cell quantitative (q)PCR. The gene-expression data for single cells correspond to the ES (*n *= 96 single ES cells in the G1 phase of the cell cycle), PrE (*n *= 96 single PrE cells in the G1 phase), ES200 (*n *= 40 single-cell-sized samples from pooled lysis of ES cells in the G1 phase), and PrE200 (*n *= 40 single-cell-sized samples from pooled lysis of PrE cells in the G1 phase) groups. The ES and PrE box plots represent the gene-expression variability, which includes the biological variability and the experimental error, while the ES200 and PrE200 box plots represent the gene-expression variability due to experimental error.

We first performed a cluster analysis of all the transcripts from all of the samples. The global expression patterns of the ES and PrE cells were clearly divided into two clusters (Figure 4a). A heat map of the ES and PrE marker genes and the non-differentially expressed genes is shown in Figure 4b. We detected 1,620 and 1,436 differentially expressed genes in the ES and PrE cells, respectively. These differentially expressed genes included the ES marker genes (for example, *Nanog*, *Pou5f1*, and *Fgf4*) and the PrE marker genes (for example, *Gata4*, *Gata6*, and *Dab2*). In addition, these marker genes had clear differential expression between the ES and PrE cells. By contrast, the non-differentially expressed genes, such as *Gnb1 l *and *Eef1b2*, did not exhibit a differential expression pattern (Figure 4b).

We then validated the expression patterns of the ES and PrE marker genes and the non-differentially expressed genes using an amplification-free single-cell qPCR method. To avoid any amplification bias, we directly detected the gene expression from single cells without amplification (see details in Materials and methods). The results show expression patterns for the ES and PrE cell markers and the non-differentially expressed genes that were highly correlated with the single-cell RNA-seq data. The gene-expression levels of *Pou5f1 *and *Zfp42 *were dramatically decreased in the majority of the single PrE cells. However, the gene-expression levels of *Pou5f1 *and *Zfp42 *remained high in a small number of single PrE cells (Figure 4c). This trend was observed in both the single-cell Quartz-Seq and the amplification-free single-cell qPCR methods.

### Single-cell Quartz-Seq detects the different cell-cycle phases of a single cell type

In addition to being a result of different cell types, gene-expression heterogeneity can also result from different cell-cycle phases. To investigate the performance limits of the single-cell Quartz-Seq method, we tested whether this method is able to distinguish cell-cycle-dependent heterogeneity among ES cells.

We performed single-cell Quartz-Seq with ES cells in different cell-cycle phases (G1, S, and G2/M) and then used principal components analysis (PCA) to analyze the results. The single PrE cells and the single ES cells formed two clearly divided clusters: the ES and PrE clusters (see Additional file 1, Figure S15a; see Additional file 5, Supplementary movie 1). When the single PrE cells were excluded, the single ES cells from each cell-cycle phase formed three different clusters, although a few single cells from the G1 and S phases were close to the G2/M cluster (see Additional file 1, Figure S15b; see Additional file 6, Supplementary movie 2). As expected, the differences between the cell-cycle phases were smaller than the difference between the stem cells and the more differentiated cells. Despite these smaller differences, the single-cell Quartz-Seq method was able to detect the different cell-cycle phases within a single cell type.

### Single-cell Quartz-Seq reveals that the gene expression fluctuations in a single cell type in the same cell-cycle phase

If the differences associated with the different cell types and cell-cycle phases are excluded, there still remains a small amount of heterogeneity due to the fluctuations in gene expression among single cells.

**Figure 5:**
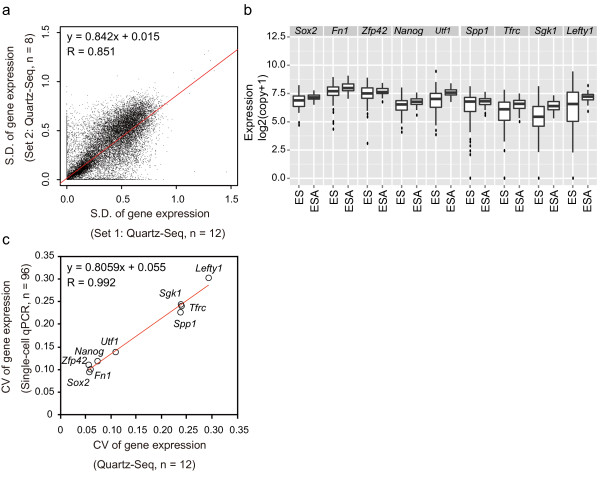
**Single-cell Quartz-Seq reveals fluctuations in global gene expression in a single cell type in the same cell-cycle phase**. **(a) **The × and Y axes represent the standard deviation (SD) of gene expression from the different datasets from single-cell Quartz-Seq with single embryonic stem (ES) cells that were all in the G1 phase of the cell cycle (*n *= 12, *n *= 8). **(b) **We detected expression of nine genes (*Fn1*, *Zfp42*, *Sgk1*, *Tfrc*, *Utf1*, *Lefty1*, *Nanog*, *Sox2*, and *Spp1*) by amplification-free single-cell quantitative (q)PCR with single ES cells in the G1 phase.The nine genes were selected from the ES cell differentiated genes (ES: fragment per kilobase of transcript per million fragments sequenced (FPKM) > 2; PrE: FPKM < 1). The plot shows the gene expression for the single-cell analysis of ES (*n *= 96 single ES cells in the G1 phase of the cell cycle) and ESA samples (*n *= 48 'averaged' single-cell samples from 300 pooled ES cells in the G1 phase). The ES single-cell box plots represent the gene-expression variability that contains the biological variability and the experimental error, while the ESA sample box plots represent the gene-expression variability due to experimental error. **(c) **The × axis represents the coefficient of variation (CV) of the gene expression from single-cell Quartz-Seq data with single ES cells in the G1 phase of the cell cycle (*n *= 12). The Y axis represents the CV of the gene expression from amplification-free single-cell qPCR with ES cells in the G1 phase of the cell cycle (*n *= 96). The CVs of the gene expression are plotted for the nine genes. The red lines represent regressions.

To test whether single-cell Quartz-Seq can detect these gene-expression fluctuations, we calculated and plotted the standard deviations from two independently amplified sets of single-cell Quartz-Seq data (*n *= 12 and *n *= 8) of ES cells in G1 phase (Figure 5a). The PCC of these standard deviations from two independent sets was approximately 0.85 (Figure 5a). We performed an F-test of the equality of the two Quartz-Seq data variances to identify reproducible gene-expression fluctuations (false-discovery rate (FDR) > 0.6). Variances in 17,064 gene expressions were reproducibly observed. These results suggest that the gene-expression fluctuations detected by single-cell Quartz-Seq are highly reproducible and thus not due to experimental errors.

To further validate the observed gene-expression fluctuations using an independent experimental method, we used amplification-free single-cell qPCR to assess the gene expression of nine genes (Figure 5b), which were selected according to their gene-expression levels. To assess both the gene-expression fluctuations and the experimental errors, we prepared samples from single cells (for the assessment of the gene-expression fluctuations) and single-cell-sized samples of pooled cells (for the assessment of the experimental errors). We expected that, if the gene-expression fluctuations detected by single-cell Quartz-Seq were not solely due to experimental errors, the gene-expression fluctuations detected by the single-cell qPCR of the single-cell samples would be greater than the experimental errors detected from the pooled samples. As expected, we found that the gene-expression fluctuations detected by single-cell qPCR for the single-cell samples were indeed significantly greater than the experimental errors detected from the pooled samples (F-test, *P*<0.001, Figure 5b). This result indicates that single-cell qPCR can clearly distinguish gene-expression fluctuations from experimental errors.

If the single-cell Quartz-Seq method quantitatively detects gene-expression fluctuations, we also expected that the gene-expression fluctuations would be highly correlated between the single-cell Quartz-Seq and the qPCR methods. To confirm this, we compared the single-cell Quartz-Seq data with the single-cell qPCR data. To compare these two different platforms (single-cell Quartz-Seq and qPCR), we chose to use a relative measure, such as coefficient of variation (CV), rather than an absolute measure, such as standard deviation. As expected, we found that the CVs of the single-cell Quartz-Seq approach were highly correlated with those obtained with single-cell qPCR (PCC for the CVs was 0.992) (Figure 5c), suggesting that the single-cell Quartz-Seq method quantitatively detects gene-expression fluctuations.

To assess the functional features of fluctuated genes, we performed over-representation analysis using the Gene Ontology and the REACTOME pathway database for single-cell Quartz-Seq with 20 mouse ES cells in G1 phase (see Additional file 1, Figure S16). First, we performed clustering using PCA to collect groups of similar fluctuated genes. Each principal component was calculated using a hypergeometric test with the Gene Ontology and REACTOME pathway database. We found that the chromosome maintenance, G1/S−specific transcription, and RNA polymerase II transcription pathways were significantly over-represented in PC 1.

## Discussion

In this study, we established a novel WTA method that is optimized for single-cell RNA-seq, and detects gene-expression heterogeneity between individual cells. This WTA method for single-cell Quartz-Seq is substantially easier to perform than other previously developed methods that are based on the poly-A-tailing reaction [18,19] (see Additional file 1, Figure S1). For example, the Kurimoto *et al*. method requires approximately 17 PCR tubes and 11 reaction steps for a single cell [18], whereas the single-cell Quartz-Seq amplification, requires only 1 PCR tube and 6 reaction steps per single cell; all of the steps are completed in a single PCR tube without any purification. These improvements, which drastically simplify the single-cell Quartz-Seq method, will be useful for high-throughput production of single-cell preparations.

In addition to its simplicity, the single-cell Quartz-Seq method is highly quantitative (Figure 2). We validated the performance of single-cell Quartz-Seq using 10 pg samples of purified total RNA prepared from pooled cell populations, and found that the quantitative performance of single-cell Quartz-Seq was better than that of previously developed single-cell methods (Figure 3). Moreover, Quartz-Seq was useful for the analysis of cell subpopulations (50 cells containing 300 to 350 pg of total RNA) with highly quantitative performance (*R *= 0.99; see Additional file 1, Figure S17).

Any method based on PCR amplification will have difficulty amplifying transcripts with an extremely high GC content, and thus we would expect these to be under-represented in the Quartz-Seq. We performed a detailed comparison of technical noise between different polymerases, and found that Quartz-Seq is more robust against high GC content when MightyAmp DNA polymerase is used for amplification of GC-rich sequences compared with Ex Taq DNA polymerase.

In the analysis of cDNA lengths in each method, we found that the unamplified isoforms from Quartz-Seq had a shorter cDNA length (mean 1,684.0 bp) than the amplified isoforms (mean 2558.6 bp). This seems counterintuitive but can be explained by the principle of massively parallel sequencing with WTA, in which a longer cDNA generates more reads and therefore can be detected more sensitively than a shorter cDNA (see Additional file 1, Figure S11d). The unamplified isoforms from Quartz-Seq also had a shorter cDNA length (mean 1,684.0 bp) compared with the mean length of all of the Ensembl Mouse Transcripts (mean 1,817 bp). The unamplified isoforms from Quartz-Seq had a significantly shorter cDNA length (mean 1,684.0 bp) than those from Smart-Seq (mean 2,382.0 bp), suggesting that Quartz-Seq is more robust against a shorter cDNA length. We also found that Smart-Seq was unable to amplify 3,924 ± 124.5 isoforms, whereas the number of isoforms that could not be amplified by Quartz-Seq was only 1,614 ± 88.9.

As a result of its higher reproducibility and sensitivity, single-cell Quartz-Seq can distinguish not only different cell types but also different cell-cycle phases of the same cell type. In addition, this method can also comprehensively detect gene-expression fluctuations within the same cell type and cell-cycle phase; these fluctuations were highly reproducible in two independent experiments (Figure 5a,c) and were distinguished from experimental errors measured from technical replicates of pooled samples (Figure 5b). Therefore, our method is capable of comprehensively and quantitatively revealing gene-expression fluctuations. Such fluctuations can be generated by both the intrinsic stochastic nature of gene expression and the extrinsic environmental differences between cells [5-8]. In fact, it has been reported that individual cells in a population of ES cells exhibit fluctuations in both mRNA and protein expression under the same culture conditions (for example, as reported for *Nanog*, *Zfp42 Whsc2*, *Rhox9*, *and Zscan4*) that might be associated with different cellular phenotypes [1,23-25]. Hence, the single-cell Quartz-Seq approach should be useful for the analysis of the roles and mechanisms of non-genetic cellular heterogeneity.

## Conclusions

Single-cell Quartz-Seq is a simplified protocol compared with previously established methods based on the poly-A-tailing reaction. All of the steps are completed in a single PCR tube without any purification. The reproducibility and sensitivity of Quartz-Seq were higher than those of other single-cell RNA-seq methods. Use of Quartz-Seq in technical replicates with 10 pg each of total RNA produced a PCC of approximately 0.93, whereas the reproducibility of previous methods is approximately 0.7. To evaluate the sensitivity of Quartz-Seq, we compared the performance of conventional RNA-seq and single-cell RNA-seq methods with 10 pg total RNA and found the PCC to be approximately 0.88 in Quartz-Seq compared with approximately 0.7 in other methods. When used in the global expression analysis of real single cells, the single-cell Quartz-Seq approach successfully detected gene-expression heterogeneity even between cells of the same cell type and/or between different cell-cycle phases. This observed gene-expression heterogeneity was found to be highly reproducible in two independent experiments, and could be distinguished from experimental errors, which were measured using technical replicates of pooled samples. Therefore, single-cell Quartz-Seq is a useful method for the comprehensive identification and quantitative assessment of cellular heterogeneity.

## Materials and methods

### Cell culture

We used EB5 ES cells for preparation of total RNA. This cell line is derived from E14tg2a ES cells, in which a blasticidin**-**resistance gene disrupts one endogenous *Pou5f1 *allele. We used 5G6GR ES cells for the single-cell Quartz-Seq. This cell line was generated by random integration of the linearized Gata6-GR-IRES-Puro vector into EB5 ES cells [21]. These cells were cultured on gelatin-coated dishes, in the absence of feeder cells and in Glasgow minimal essential medium (GMEM; Sigma-Aldrich, St Louis, MO, USA) supplemented with 10% fetal calf serum, 1000 U/ml leukemia inhibitory factor (ESGRO; Invitrogen Corp., Carlsbad, CA, USA), 100 µmol/l 2-mercaptoethanol (Nacalai Tesque Inc., Kyoto, Japan), 1× non-essential amino acids (Invitrogen), 1 mmol/l sodium pyruvate (Invitrogen), 2 mmol/l L-glutamine (Nacalai Tesque), 0.5× penicillin/streptomycin (Invitrogen), and 10 µg/ml blasticidin (Invitrogen). For culture of 5G6GR ES cells, 0.5 µg/ml puromycin (Sigma-Aldrich) was also added to the culture. For differentiation of 5G6GR ES cells into PrE cells, the cells were seeded into medium supplemented with 100 mmol/l dexamethasone instead of blasticidin, and cultured for 72 hours. After this 72 hours culture, the 5G6GR cells had completely differentiated into PrE cells.

### RNA preparation

The total RNA was purified from the ES cells (EB5 cell line) using reagent (TRIzol; Life Technologies Corp., Carlsbad, CA, USA) and a commercial kit (RNeasy Mini Kit; Qiagen Inc., Valencia, CA, USA). The amount of total RNA from an ES cell was quantified using an absorptiometer (ND-1000; LMS, Tokyo, Japan). The length distribution of the total RNA was measured using a RNA 6000 Nano Kit (Agilent Biotechnology, Santa Clara, CA, USA), which produced an RNA integrity number of 10 for the total RNA. The spike RNAs were synthesized using the pGIBS-LYS, pGIBS-DAP, pGIBS-PHE, and pGIBS-THR plasmids (American Type Culture Collection (ATCC), Manassas, VA, USA) and the MEGAscript T3 kit (Ambion Inc., Austin, TX, USA), as previously reported [12]. The spike RNAs were added to the total RNA from the ES cells as follows (per 10 pg of total RNA): *Lys*, 1000 copies; *Dap*, 100 copies; *Phe*, 20 copies; and *Thr*, 5 copies. The total RNA containing the spike RNAs was diluted to 25 pg/µl using single-cell lysis buffer (0.5% NP40, Thermo) immediately before amplification.

### Single-cell collection using FACS

The cultured cells were dissociated using trypsin-EDTA at 37°C for 3 minutes. The resulting cells were subsequently washed with PBS buffer. A total of 0.5 × 10^6 ^cells were stained with 1 ml of PBS containing 10 µg/ml Hoechst 33342 at 37°C for 15 minutes. The stained cells were sorted as previously reported, based on the Hoechst 33342-stained cell area of the FACS distribution [22]. To increase the amplification success rate, we added several bubbles to the single-cell lysis buffer using a micropipette (see Additional file 1, Figure S13). We sorted each single cell into a 0.4 µl aliquot of lysis buffer with a bubble; the buffer was pre-chilled to 0°C using a PCR chill rack (IsoFreeze; Labgene Scientific, Châtel-St-Denis, Switzerland). Subsequently, we performed WTA with each single-cell lysis sample. For details of all of the samples used, see Additional file 7, Table S2.

### Whole-transcript amplification for single-cell Quartz-Seq

To reduce the risk of RNase contamination, the workbench environment and all experimental equipment were cleaned using an RNase removal reagent (RNase Out; Molecular BioProducts, San Diego, CA, USA). We used low-retention single PCR tubes or sets of 8-linked PCR tubes for single-cell amplifications (TaKaRa Bio Inc., Otsu, Japan). The cells and 10 pg total RNA samples were dissolved in 0.4 µl of single-cell lysis buffer (0.5% NP40) in an aluminum PCR rack at 0°C and transferred to ice. These solutions were mixed using a bench-top mixer (MixMate; Eppendorf, Westbury, NY, USA) at 2,500 rpm and 4°C for 15 seconds and then at 3000 × *g *and 4°C for 10 seconds. Immediately after the second centrifugation, 0.8 µl of priming buffer (1.5× PCR buffer with MgCl_2 _(TaKaRa Bio), 41.67 pmol/l of the RT primer (HPLC-purified; Table 1), 4 U/µl of RNase inhibitor (RNasin Plus; Promega Corp., Madison, WI, USA), and 50 µmol/l dNTPs were added to each tube. The solutions were mixed at 2,500 rpm and 4°C for 15 seconds. The denaturation and priming were performed at 70°C for 90 seconds and 35°C for 15 seconds using a thermal cycler (C1000 and S1000; Bio-Rad Laboratories, Inc., Hercules, CA, USA), and the reaction tubes were placed into an aluminum PCR rack at 0°C. Subsequently, 0.8 µl of RT buffer (1× PCR buffer, 25 U/µl reverse transcriptase (SuperScript III; Life Technologies), and 12.5 mmol/l DTT) was added to each tube. The reverse transcription was performed at 35°C for 5 minutes and 45°C for 20 minutes, and the reactions were heat-inactivated at 70°C for 10 minutes. The reaction tubes were then placed into an aluminum PCR rack at 0°C. We consistently used the latest available lots of the reverse transcriptase (SuperScript III) for the single-cell amplification. After centrifugation at 3000 × *g *and 4°C for 10 seconds, 1 µl of the exonuclease solution (1× Exonuclease buffer and 1.5 U/µl exonuclease I; both TaKaRa Bio) was added to each tube. The primer digestion was performed at 37°C for 30 minutes, and the reactions were heat-inactivated at 80°C for 10 minutes. The reaction tubes were placed into an aluminum PCR rack at 0°C. After centrifugation at 3,000 × *g *and 4°C for 30 seconds, 2.5 µl of poly-A-tailing buffer (1× PCR buffer, 3 mmol/l dATP, 33.6 U/µl terminal transferase (Roche Applied Science, Indianapolis, IN, USA), and 0.048 U/µl RNase H (Invitrogen) was added to each tube in the aluminum PCR rack at 0°C. The reaction tubes were mixed at 2,500 rpm and 4°C for 15 seconds. Immediately after centrifugation at 3000 × *g *and 0°C for 10 seconds, the reaction tubes were placed into a thermal cycler block, which was pre-chilled to 0°C. Subsequently, the poly-A-tailing reaction was performed at 37°C for 50 seconds and heat-inactivated at 65°C for 10 minutes. The reaction tubes were then placed into an aluminum PCR rack at 0°C. After centrifugation at 3000 × *g *and 4°C for 30 seconds, the reaction tubes were placed into an aluminum PCR rack at 0°C. We then added 23 µl of the second-strand buffer (1.09× MightyAmp Buffer v2 (TaKaRa), 70 pmol/l tagging primer (HPLC-purified; Table 1), and 0.054 U/µl MightyAmp DNA polymerase (TaKaRa)) to each tube. The reaction tubes were mixed at 2500 rpm and 4°C for 15 seconds. After centrifugation at 3000 × *g *and 4°C for 10 seconds, the second-strand synthesis was performed at 98°C for 130 seconds, 40°C for 1 minute, and 68°C for 5 minutes. The reaction tubes were then immediately placed into an aluminum PCR rack at 0°C, and 25 µl of PCR buffer (1× MightyAmp Buffer version 2 and 1.9 µmol/l suppression PCR primer (HPLC-purified; Table 1)) was added. The reaction tubes were mixed at 2,500 rpm and 4°C for 15 seconds. After centrifugation at 3000 × *g *and 4°C for 10 seconds, the PCR enrichment was performed using the following conditions per cycle for a total of 21 PCR cycles: 98°C for 10 seconds, 65°C for 15 seconds, and 68°C for 5 minutes. After the PCR step, the reaction tubes were incubated at 68°C for 5 minutes. The reaction tubes were then placed into an aluminum PCR rack at 25°C. The amplified cDNA was purified using a PCR purification column (MinElute; Qiagen) or a PCR purification bead system (Agencourt AMPure XP; Beckman Coulter Inc., Brea, CA, USA). The obtained amplified cDNA was used for subsequent detection by each platform.

**Table 1:** Primers used in the various reactions

**Primer**	**Sequence 5'→'**
RT primer (WTA)	TATAGAATTCGCGGCCGCTCGCGATAATACGACTCACTATAGGGCGTTTTTTTTTTTTTTTTTTTTTTTT
Tagging primer	TATAGAATTCGCGGCCGCTCGCGATTTTTTTTTTTTTTTTTTTTTTTT
Suppression primer	(NH**_2_**)-GTATAGAATTCGCGGCCGCTCGCGAT
TRSU	AATGATACGGCGACCACCGAGATCTACACTCTTTCCCTACACGACGCTCTTCCGATC*T
TRSI-2	(5'-phosphate)GATCGGAAGAGCACACGTCTGAACTCCAGTCACCGATGTATCTCGTATGCCGTCTTCTGCTT*G
TRSI-4	(5'-phosphate)GATCGGAAGAGCACACGTCTGAACTCCAGTCACTGACCAATCTCGTATGCCGTCTTCTGCTT*G
TRSI-5	(5'-phosphate)GATCGGAAGAGCACACGTCTGAACTCCAGTCACACAGTGATCTCGTATGCCGTCTTCTGCTT*G
TRSI-6	(5'-phosphate)GATCGGAAGAGCACACGTCTGAACTCCAGTCACGCCAATATCTCGTATGCCGTCTTCTGCTT*G
TRSI-7	(5'-phosphate)GATCGGAAGAGCACACGTCTGAACTCCAGTCACCAGATCATCTCGTATGCCGTCTTCTGCTT*G
TRSI-12	(5'-phosphate)GATCGGAAGAGCACACGTCTGAACTCCAGTCACCTTGTAATCTCGTATGCCGTCTTCTGCTT*G
TPC1	AATGATACGGCGACCACCGA*G
TPC2	CAAGCAGAAGACGGCATACGA*G
RT primer (qPCR)	TATAGAATTCGCGGCCGCTCGCGATAATACGACTCACTATAGGGCGTTTTTTTTTTTTTTTTTTTTTTTT

qPCR, quantiative PCR, WTA, whole-transcript amplification.

*Indicates phosphorothioate bonds

For the Smart-Seq analysis, we amplified cDNA from 10 pg of the total RNA from ES cells using a commercial kit (SMARTer Ultra Low RNA Kit for Illumina sequencing; Clontech, Mountain View, CA, USA). After 19 cycles of PCR enrichment from the 10 pg of total ES-cell RNA, 2 to 3 ng of amplified cDNA were obtained.

### Library preparation for single-cell Quartz-Seq

We prepared a library for conventional RNA-seq (with non-WTA) using a commercial kit (TruSeq RNA Sample Kit; Illumina Inc., San Diego, CA, USA) in accordance with the manufacturer's protocol with the exception of the PCR enrichment, for which we used a different polymerase (HiFi DNA polymerase; Kapa Biosystems Inc., Woburn, MA, USA).

To prepare the library for Quartz-Seq (with WTA) and Smart-Seq (with WTA), we prepared a DNA sequencing library using our optimized library preparation method, which we call ligation-based Illumina multiplex library preparation (LIMprep). For LIMprep, we used the same library preparation kit as before (Kapa Biosystems) and self-produced the TruSeq (Illumina) adaptors and PCR primers. For Quartz-Seq, 20 ng of amplified cDNA was diluted in 130 µl of Tris-EDTA (TE) buffer. The solutions were transferred into snap-cap microtubes (Covaris Inc., Woburn, MA, USA). The amplified cDNAs in the microtubes were fragmented using a focused ultrasonicator (model S220; Covaris). The ultrasonication process was configured as follows: duty factor 10%; peak incident power 175 W; cycles per burst 100; and time 600 seconds. The fragmented cDNA was purified into 10 µl of nuclease-free water using a concentrating column (DNA Clean and Concentrator-5; Zymo Research, Irvine, CA. USA). Subsequently, 40 µl of the reaction mix (1.25× End Repair Buffer and 1.25× End Repair Enzyme Mix) (Kapa Biosystems) was added to 10 µl of the fragmented cDNA solution. The end-repair reaction was performed at 20°C for 30 minutes. The end-repaired DNA was purified into 12.5 µl of EB1/10 buffer (1 mmol/l Tris-HCl pH 8.0) using a concentrating column as before (DNA Clean and Concentrator-5; Zymo Research). Subsequently, 12.5 µl of the A-tailing mix (2× A-tailing Buffer and 2× A-tailing Enzyme) was added to 12.5 µl of the end-repaired DNA solution. The A-tailing reaction was performed at 30°C for 30 minutes. The A-tailed DNA was purified into 12.5 µl of EB1/10 buffer using a concentrating column as before (DNA Clean and Concentrator-5; Zymo Research). Subsequently, 12.5 µl of the adaptor ligation mix (2× ligation buffer, 2× DNA ligase, and 10 pmol of each self-produced adaptor) was added to 12.5 µl of the A-tailed DNA solution at 4°C. The adaptor ligation was performed at 20°C for 15 minutes. For the adaptor ligation, we used 10 pmol of the self-produced TruSeq adaptor per sample. Each self-produced TruSeq adaptor was prepared using the following HPLC-purified primers (Hokkaido System Science Co. Ltd., Sapporo, Japan): TRSU, TRSI-2, TRSI-4, TRSI-5, TRSI-6, TRSI-7, and TRSI-12 (Table 1).

Each primer was dissolved in the adaptor buffer (10 mmol/l Tris-HCl pH 7.8, 0.1 mmol/l EDTA pH 8.0, and 50 mmol/l NaCl) to a concentration of 100 µmol/l. Equal amounts of 100 µmol/l TRSU and 100 µmol/l of each TRSI primer were added to the PCR tubes. After mixing, these primers were incubated at 95°C for 2 minutes. The primer annealing was then performed (95°C for 2 minutes, followed by a temperature decrease of -0.5°C per cycle for 170 cycles). Subsequently, the reaction tubes were incubated at 4°C for 5 minutes. The resulting adaptors were diluted with adaptor buffer to a concentration of 10 µmol/l. We prepared 1 µl aliquots with 10 µ° of each adaptor, which were stored at -80°C until use. The removal of the adaptor dimer was performed as follows: 25 µl of binding support buffer (1 mol/l NaCl, 20 mmol/l MgCl_2_, and 20 mmol/l Tris-HCl pH 7.8) and 60 µl of bead solution (Agencourt AMPure XP; Beckman Coulter) was added to 25 µl of the adaptor-ligated DNA solution. After 15 minutes of incubation at 25°C, the beads were separated using a magnetic stand for at least 10 minutes. Then washed twice with 80% ethanol for 1 minute. The adaptor-ligated DNA was eluted with 25 µl of EB1/10. The purification step for the removal of the adaptor dimer was repeated. Finally, the adaptor-ligated DNA was eluted with 20 µl of EB1/10. A volume of 30 µl of the PCR solution (1.666× HiFi DNA polymerase ready mix (Kapa Biosystems), 17.5 pmol TPC1 primer, and 17.5 pmol TPC2 primer) was added to 20 µl of the adaptor-ligated DNA. The primer sequences are shown in Table 1. Each primer was dissolved in the adaptor buffer (10 mmol/l Tris-HCl pH 7.8, 0.1 mmol/l EDTA pH 8.0, and 50 mmol/l NaCl) to a concentration of 100 µmol/l. Prior to PCR enrichment, the reaction tubes were incubated at 98°C for 45 seconds, and the PCR enrichment was then performed (98°C for 15 seconds, 60°C for 30 seconds and 72°C for 30 seconds per cycle). Typically, 10 to 12 PCR cycles were used. After the PCR enrichment, the DNA sequencing library was purified (Agencourt Ampure XP beads; Beckman Coulter). The concentrations of the DNA sequencing library, including the TruSeq Index sequence, were estimated using a library quantification kit (Kapa Biosystems). The PCC for three technical replicates of the LIMprep protocol was 0.9976 ± 0.0005.

### GeneChip

The cDNA was synthesized from 0.25 µg of total RNA using random six-mers (Promega) and reverse transcriptase (SuperScript II; Invitrogen) in accordance with the Illumina standard protocol. The cDNA synthesis, cRNA labeling reactions, and hybridization to the high-density oligonucleotide arrays for *Mus musculus *(Mouse Genome 430 Array; Affymetrix Inc., Santa Clara, CA, USA) were performed in accordance with the instructions detailed in the manufacturer's instructions (Expression Analysis Technical Manual; Affymetrix). For single-cell Quartz-Chip, 10 ng of amplified cDNA was used in the cRNA labeling reactions. The expression values of the Kurimoto *et al*. and the Quartz-Chip methods were quantified using the RMA method. All of the data were normalized using the quantile normalization method to compare the expression values between the different microarrays [26].

### Bioinformatics analysis

All raw sequencing reads were trimmed using Trimmomatic software to remove the sequencing and WTA primers. All of the trimmed sequence reads were mapped to the mouse reference genome (mm9) using TopHat (version 2.0.3 [27]) with the default parameters. FPKMs were calculated using Cufflinks (version2.0.1 [28]) with a transcriptome reference (Ensembl Mouse Transcript). The sample clustering of the single-cell RNA-seq data was performed using R software and the pvclust package [29] with 1000 bootstrap resamplings and Ward distance functions. All of the data were visualized using R, the ggplot2 package, and the cummeRbund software.

To identify the significant differential expression between two differentiated cell states from single-cell RNA-seq data, we performed the Wilcoxon rank test, and calculated the mutual information between the gene-expression distributions of the ES cells and the PrE cells using an empirical Bayes estimator.

Although our novel single-cell RNA-seq method is highly sensitive, cost-effective, and easy to perform, it is not completely without amplification bias. To identify the differentially expressed genes from the single-cell RNA-seq method with small sample datasets, we inferred mutual information between the gene-expression distributions of the two types of cells using an empirical Bayes estimator: the mutual information MI(X, Y) for pairs of cell states × and Y, where × and Y may, for example, represent expression levels of the two cell states. The MI is considered the Kullback-Leibler distance from the joint probability density to the product of the marginal probability densities as follows:

The MI is always non-negative, symmetric, and equal to 0 only if × and Y are independent. The MI can be represented as a summation of entropies:

To infer the entropy from gene-expression data with a small sample size, we applied an empirical Bayes approach, namely, the so-called James-Stein estimator [30]. First, the gene-expression data were made discrete using the Freedman and Diaconis algorithm to determine the number of bins and the width of the histograms. We then estimated the K2 cell frequencies of the K × K contingency table for each cell-state pair × and Y using the James-Stein estimator. Finally, we calculated H(X), H(Y), H(X, Y) and MI(X, Y).

To define the reproducibility of the variation in the measured expression levels between the single cells analyzed using Quartz-Seq, we used FDR to apply the F-test to two independent Quartz-Seq datasets with multi-testing adjustments.

### Accession codes

All data are available on GEO [GSE42268].

### qPCR validation of reverse-transcribed cDNA and quantification of amplified cDNA

To validate the quantification of the reverse-transcribed cDNA and the amplified cDNA, we detected the gene expression using a SYBR Green system (Power SYBR Green PCR Master Mix; Applied Biosystems, Foster City, CA, USA) and qPCR primers as described previously [12]. For the qPCR primer sets for each gene, see Additional file 8, Table S3.

The non-WTA sample was prepared by reverse transcription using 200 ng of total ES-cell (EB5 cell line) RNA containing the spike RNAs (*Lys*, *Dap*, *Phe*, and *Thr*). A sample of 200 ng total RNA was dissolved in 10 µl of single-cell lysis buffer (0.5% NP40) on an aluminum PCR rack at 0°C and placed on ice. Immediately after, 20 µl of priming buffer (1.5× PCR buffer with MgCl_2 _(TaKaRa), 41.67 pmol/l of RT primer (HPLC-purified; Table 1), 4 U/µl RNase (RNasin Plus; Promega), and 50 µmol/l dNTPs) was added to each tube. The solution was mixed at 2,500 rpm and 4°C for 15 seconds. The denaturation and priming were performed at 70°C for 90 seconds and 35°C for 15 seconds using a thermal cycler (C1000 and S1000; Bio-Rad Laboratories), and the reaction tubes were placed in an aluminum PCR rack at 0°C, then 20 µl of RT buffer (1× PCR buffer, 25 U/µl SuperScript III (Life Technologies), and 12.5 mmol/l DTT) was added to each tube. The RT was performed at 35°C for 5 minutes and 45°C for 18 minutes and then heat-inactivated at 70°C for 10 minutes. Subsequently, the reaction tubes were placed in an aluminum PCR rack at 0°C.

### Amplification-free single-cell qPCR

We used a semi-skirted 96-well PCR plate for the amplification-free single-cell qPCR. Single cells in the G1 cell-cycle phase were individually collected into 1 µl of lysis buffer (0.25% NP40 and 1 U/µl RNasin (Promega) plus RNase inhibitor) at 4°C in a PCR chill rack (IsoFreeze; Labgene Scientifid). After centrifugation at 2000 × *g *and 4°C for 2 minutes, these solutions were mixed at 2,000 rpm for 15 seconds. To detect the variability of the reverse transcription of each gene, we prepared an 'averaged' single-cell pooled sample. For the pooled sample, 200 single cells in the G1 phase were collected into 200 µl of lysis buffer. After mixing, 1 µl of the pooled lysis solution was dispensed into each well of a 96-well PCR plate. Subsequently, 2 µl of RT buffer (1.5× VILO Reaction Buffer (contains random primers) and 1.5× SuperScript Enzyme Mix; both Invitrogen) was added to each well. These plates were incubated as follows: 25°C for 10 minutes, 42°C for 60 minutes, and 85°C for 10 minutes. Then 15 µl of the qPCR dilution solution (0.0001% NP40) was added and mixed well. Mouse genomic DNA was used to normalize the qPCR quantification. To a 384-well qPCR plate, we added 7 µl of the qPCR solution (1.4× QuantiTect SYBR Green PCR Master Mix (Qiagen), 5 pmol forward primer, and 5 pmol reverse primer) and 3 µl of diluted solution were added The qPCR plate was incubated at 95°C for 15 minutes. Subsequently, qPCR was performed for 45 cycles, which consisted of 95°C for 15 seconds, and 60°C for 1 minute. The data were collected at 60°C. For the primer sets for each gene, see Additional file 8, Table S3.

## Abbreviations

cDNA: complementary DNA; cRNA: complementary RNA; CV: Coefficient of variation; DTT: dithiothreitol; EDTA: ethylenediamene tetraacetic acid; ES cell: Embryonic stem cell; FACS: fluorescence-activated cell sorting; FDR: False-discovery rate; FPKM: Fragment per kilobase of transcript per million fragments sequenced; GEO: Gene Expression Omnibus; GMEM: Glasgow minimal essential medium; HPLC: high-performance liquid chromatography; IVT: *In vitro *transcription; LIMprep: ligation-based Illumina multiplex library preparation; PBS: phosphate-buffered saline; PCA: Principal component analysis; PCC: Pearson correlation coefficient; qPCR: Quantitative polymerase chain reaction; PE: Paired-end; PrE: Primitive endoderm; RT: Reverse transcription; RMA: Robust multi-array averaging; RNA-seq: RNA sequencing; RPKM: Reads per kilobase of exon model per million mapped reads; rRNA: ribosomal RNA; RT: Reverse transcription; SE: Single-end; Seq: sequencing; SRA: Sequence Read Archive; STRT: Single-cell tagged RT sequencing; tpm: Tag per million; TE: Tris-EDTA; WTA: Whole-transcript amplification

## Competing interests

The authors declare that they have no competing interests.

## Authors' contributions

YS, IN, and HRU designed and configured the various approaches used in this study. YS performed the majority of the experiments. IN analyzed the data and developed the bioinformatics methods and tools. TH performed the cell sorting and assisted with the amplification-free single-cell qPCR. HD assisted with the cellular experiments. KDU assisted with the GeneChip experiments. TI assisted with the discussion on the amplification of small amounts of RNA. YS, IN, and HRU prepared the figures and wrote the manuscript. All authors read and approved the final manuscript.

## Supplementary Material

Additional file 1 **Figure S1: **Schematic of the whole-transcript amplification methods based on the poly-A-tailing reaction. **Figure S2: **Improvement parameters of whole-transcript amplification for Quartz-Seq. **Figure S3: **Key steps for robust suppression of byproducts. **Figure S4: **Optimization of suppression PCR for Quartz-Seq. **Figure S5: **Optimal DNA polymerase for whole-transcript amplification. **Figure S6: **Quality check of the library preparation for single-cell Quartz-Seq. **Figure S8: **Percentage of sequence reads of the suppression PCR primer or rRNA. **Figure S9: **Relationship between the read number and the reproducibility. **Figure S10: **Optimization of cDNA length in technical development for single-cell Quartz-Seq. **Figure S11: **Trend of unamplified isoforms in each single-cell RNA-seq method. **Figure S12: **Amplified cDNA lengths resulting from single-cell RNA-seq methods. **Figure S13: **Success rate of whole-transcript amplification from single cells sorted by fluorescence-activated cell sorting (FACS). **Figure S14: **Amount of total RNA from a single cell at each cell-cycle phase. **Figure S15: **Principal component analysis (PCA) of single cells from different cell types at different cell-cycle phases. **Figure S16: **Over-representation analyses for principal component (PC) of single cells from same cell types in the same cell-cycle phase (G1). **Figure S17: **Scatter plots of conventional RNA-seq and Quartz-Seq using 50 ES cells in the G1 phase of the cell cycle and Quartz-Seq using 10 pg of total ES RNA. **Figure S18: **Effect of carried-over buffer for PCR efficiency. Click here for file

Additional file 2 **Supplementary note**. Click here for file

Additional file 3 **Figure S7: **All scatter plots Click here for file

Additional file 4 **Table S1**. All results of linear regression and correlation analyses. Click here for file

Additional file 5 **Supplementary movie 1**. Principal component analysis (PCA) with single-cell Quartz-Seq data of embryonic stem (ES) and primitive endoderm (PrE) single-cell preparations. Click here for file

Additional file 6 **Supplementary movie 2**. Principal component analysis (PCA) with single-cell Quartz-Seq data of embryonic stem (ES) cells in different cell-cycle phases. Click here for file

Additional file 7 **Table S2**. Sequencing information. Click here for file

Additional file 8 **Table S3**. Primer information. Click here for file
