# Erratum to: Quartz-Seq: a highly reproducible and sensitive single-cell RNA sequencing method, reveals non-genetic gene-expression heterogeneity

**DOI:** 10.1186/s13059-017-1154-x

**Published:** 2017-01-18

**Authors:** Yohei Sasagawa, Itoshi Nikaido, Tetsutaro Hayashi, Hiroki Danno, Kenichiro D Uno, Takeshi Imai, Hiroki R. Ueda

**Affiliations:** 1 Functional Genomics Unit, RIKEN Center for Developmental Biology, 2-2-3 Minatojima-minamimachi, Chuo-ku, 650-0047 Kobe, Hyogo Japan; 2 Genome Resource and Analysis Unit, RIKEN Center for Developmental Biology, 2-2-3 Minatojima-minamimachi, Chuo-ku, 650-0047 Kobe, Hyogo Japan; 3 Laboratory for Systems biology, RIKEN Center for Developmental Biology, 2- 2-3 Minatojima-minamimachi, Chuo-ku, 650-0047 Kobe, Hyogo Japan; 4 Laboratory for Sensory Circuit Formation, RIKEN Center for Developmental Biology, 2-2-3 Minatojima-minamimachi, Chuo-ku, 650-0047 Kobe, Hyogo Japan; 5 JST, PRESTO, 2-2-3 Minatojima-minamimachi, Chuo-ku, 650-0047 Kobe, Hyogo Japan; 6 Laboratory for Synthetic Biology, Quantitative Biology Center, RIKEN, 2-2-3 Minatojima-minamimachi, Chuo-ku, 650-0047 Kobe, Hyogo Japan; 7 Bioinformatics Research Unit, Advanced Center for Computing and Communication, RIKEN, 2-1 Hirosawa, 351-0198 Wako, Saitama Japan

## Erratum

After publication of our article [1], we noticed some errors. In this manuscript, we amplified cDNA with 41.67 nmol/l RT primer and 70 nmol/l tagging primer for single-cell Quartz-Seq, and not pmol/l as originally stated. In addition, two mathematical expressions were inadvertently omitted.

Thus, in the section *Whole-transcript amplification for single-cell Quartz-Seq*, the following are correct:

Immediately after the second centrifugation, 0.8 μl of priming buffer (1.5× PCR buffer with MgCl2 (TaKaRa Bio), 41.67 nmol/l of the RT primer (HPLC-purified; Table 1), 4 U/μl of RNase inhibitor (RNasin Plus; Promega Corp., Madison, WI, USA), and 50 μmol/l dNTPs were added to each tube.

And

We then added 23 μl of the second-strand buffer (1.09× MightyAmp Buffer v2 (TaKaRa), 70 nmol/l tagging primer (HPLC-purified; Table 1), and 0.054 U/μl MightyAmp DNA polymerase (TaKaRa)) to each tube.

In the section *Bioinformatics analysis*, the equations should appear as follows:

The MI is considered the Kullback–Leibler distance from the joint probability density to the product of the marginal probability densities as follows:

1 $$ MI\left(X,Y\right)={E}_{f\left(x,y\right)}\left\{ \log \frac{f\left(x,y\right)}{f(x)f(y)}\right\} $$

The MI is always non-negative, symmetric, and equal to 0 only if × and Y are independent. The MI can be represented as a summation of entropies:

2 $$ MI\left(X,Y\right)=H(X)+H(Y)-H\left(X,Y\right) $$
