# Supplementary material for: Quartz-Seq: a highly reproducible and sensitive single-cell RNA sequencing method, reveals non-genetic gene-expression heterogeneity
Source: Genome Biol. 2013 Apr 17;14(4):R31. doi: 10.1186/gb-2013-14-4-r31 (PMC4054835; doi:10.1186/gb-2013-14-4-r31)
Supplement: Additional file 1 — Figure S1: Schematic of the whole-transcript amplification methods based on the poly-A-tailing reaction. Figure S2: Improvement parameters of whole-transcript amplification for Quartz-Seq. Figure S3: Key steps for robust suppression of byproducts. Figure S4: Optimization of suppression PCR for Quartz-Seq. Figure S5: Optimal DNA polymerase for whole-transcript amplification. Figure S6: Quality check of the library preparation for single-cell Quartz-Seq. Figure S8: Percentage of sequence reads of the suppression PCR primer or rRNA. Figure S9: Relationship between the read number and the reproducibility. Figure S10: Optimization of cDNA length in technical development for single-cell Quartz-Seq. Figure S11: Trend of unamplified isoforms in each single-cell RNA-seq method. Figure S12: Amplified cDNA lengths resulting from single-cell RNA-seq methods. Figure S13: Success rate of whole-transcript amplification from single cells sorted by fluorescence-activated cell sorting (FACS). Figure S14: Amount of total RNA from a single cell at each cell-cycle phase. Figure S15: Principal component analysis (PCA) of single cells from different cell types at different cell-cycle phases. Figure S16: Over-representation analyses for principal component (PC) of single cells from same cell types in the same cell-cycle phase (G1). Figure S17: Scatter plots of conventional RNA-seq and Quartz-Seq using 50 ES cells in the G1 phase of the cell cycle and Quartz-Seq using 10 pg of total ES RNA. Figure S18: Effect of carried-over buffer for PCR efficiency. [file gb-2013-14-4-r31-S1.PDF]

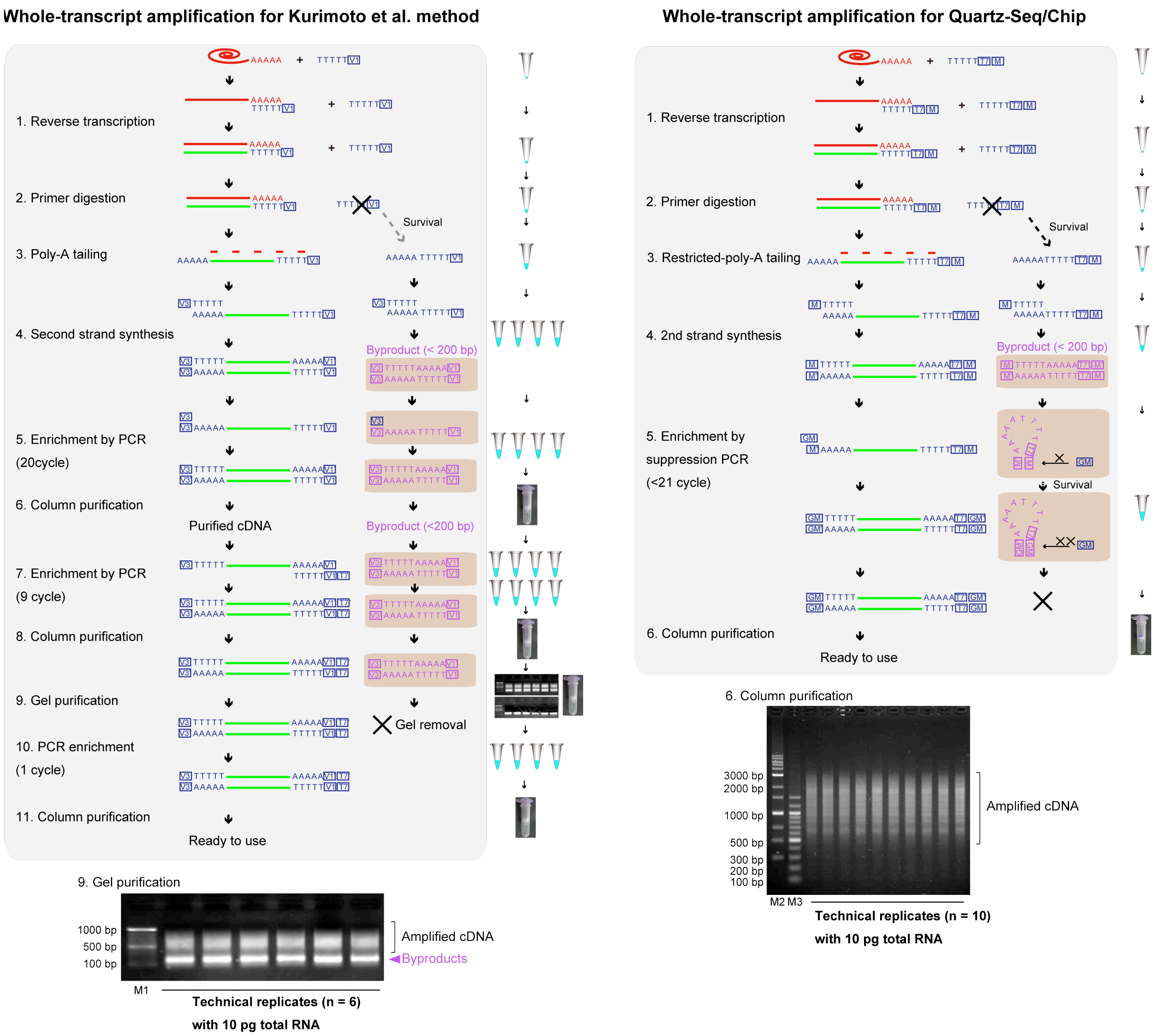

Figure S1 Schematic of the whole-transcript amplification methods based on the poly-A-tailing reaction.

The synthesis of byproducts was completely suppressed in WTA for the Quartz-Seq method. After a one-step purification with a Qiagen MinElute column, we obtained cDNA without any byproducts. In contrast, large amount of byproducts were synthesized in the PCR enrichment step of a previous method (Kurimoto et al.); these should then be removed by gel-purification prior to analysis. The amplified cDNA is purified using the Qiagen Gel Extraction Kit. The left gel image shows the amplified cDNA just before the gel-purification in the Kurimoto et al. method. These cDNAs were amplified according to the Kurimoto et al. method with 10 pg of total RNA and then separated using 3 % gel-electrophoresis. The amplified cDNA from the upper broad band was collected and purified. The right gel image shows the amplified cDNA in our whole-transcript amplification method with 10 pg of total RNA. M1: 100-bp ladder marker (Invitrogen). M2: 1-kbp ladder marker (Nacalai Tesque). M3: 100-bp ladder marker (Nacalai Tesque).

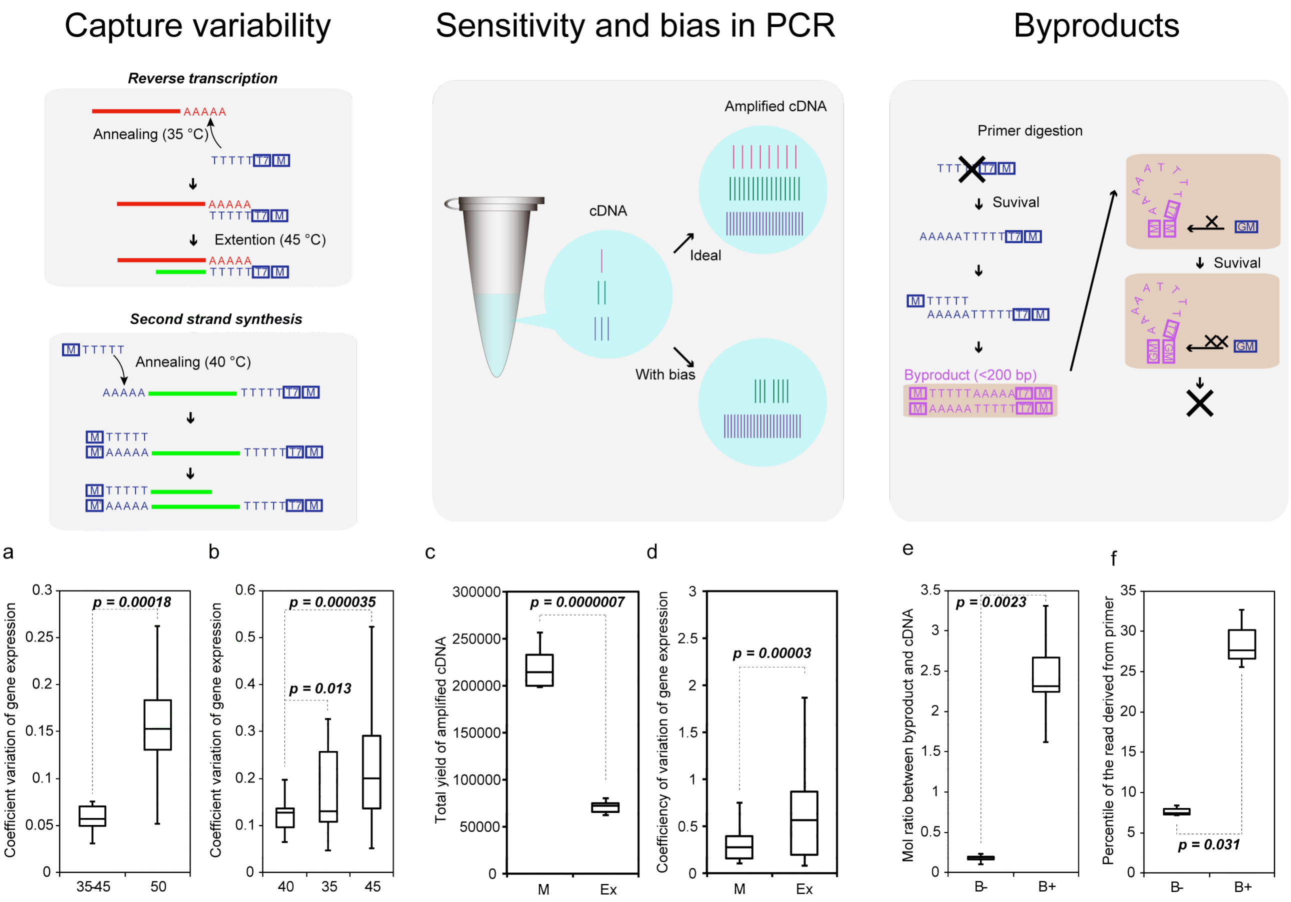

Figure S2 Improvement parameters of whole-transcript amplification for Quartz-Seq.
(a) The optimal reaction temperature steadily reduced the variability of the reverse-transcription. The box plots represent the coefficient of variation (CV) of the gene expressions from technical replicates. A total of 50 pg of total RNA was reverse transcribed with 10 μl of RT buffer using Quartz-Seq at different temperature conditions (n = 5 for each condition). The reverse transcription was performed at the following conditions: 35 °C for 5 min, 45 °C for 20 min (indicated as 35-45; used in Quartz-Seq) or 50 °C for 20 min (indicated as 50; used in previous method). After the reverse transcription, the RT solution was diluted with nuclease-free water. We then directly detected the endogenous expression of 8 genes using the prism7900 qPCR machine (ABI). The detected genes were the following: *Atp5a*, *Ddb1*, *Cox8a*, *Nanog*, *Sox2*, *Prdx1*, *Ywhae* and *Trim28*. **(b)** The optimal annealing temperature reduced the variability of the second-strand synthesis. The box plots represent the coefficient of variation (CV) of the genes expression obtained from technical replicates. We amplified the cDNA from 10 pg of total RNA (n = 4 per different condition). The annealing conditions in the second strand synthesis were as follows: 40 °C for 1 min (indicated as 40; used in Quartz-Seq), 35 °C for 1 min (indicated as 35), and 45 °C for 1 min (indicated as 45). We detected the expression of 10 endogenous genes by qPCR: *Tbp*, *Pou5f1*, *Actb*, *Lefty1*, *Cox8a*, *Nanog*, *Sox2*, *Prdx1*, *Ywhae* and *Trim28*. (c, d) The MightyAmp DNA polymerase improved the yield of amplified cDNA and the amplification reproducibility. The box plots represent the total yield of amplified cDNA or the CV of the gene expression from 8 technical replicates using MightyAmp (M, used in Quartz-Seq) and Ex Taq DNA polymerase (Ex, used in the previous method). The details are shown in Figure S5. **(e)** Molar ratio between the byproducts (50-300 bp) and the cDNA (300-8000 bp) in each condition (“B-”, with complete byproduct removal treatment; “B+”, with partial byproduct removal treatment). **(f)** Percentage of read number derived from the WTA adaptor sequences in each condition.

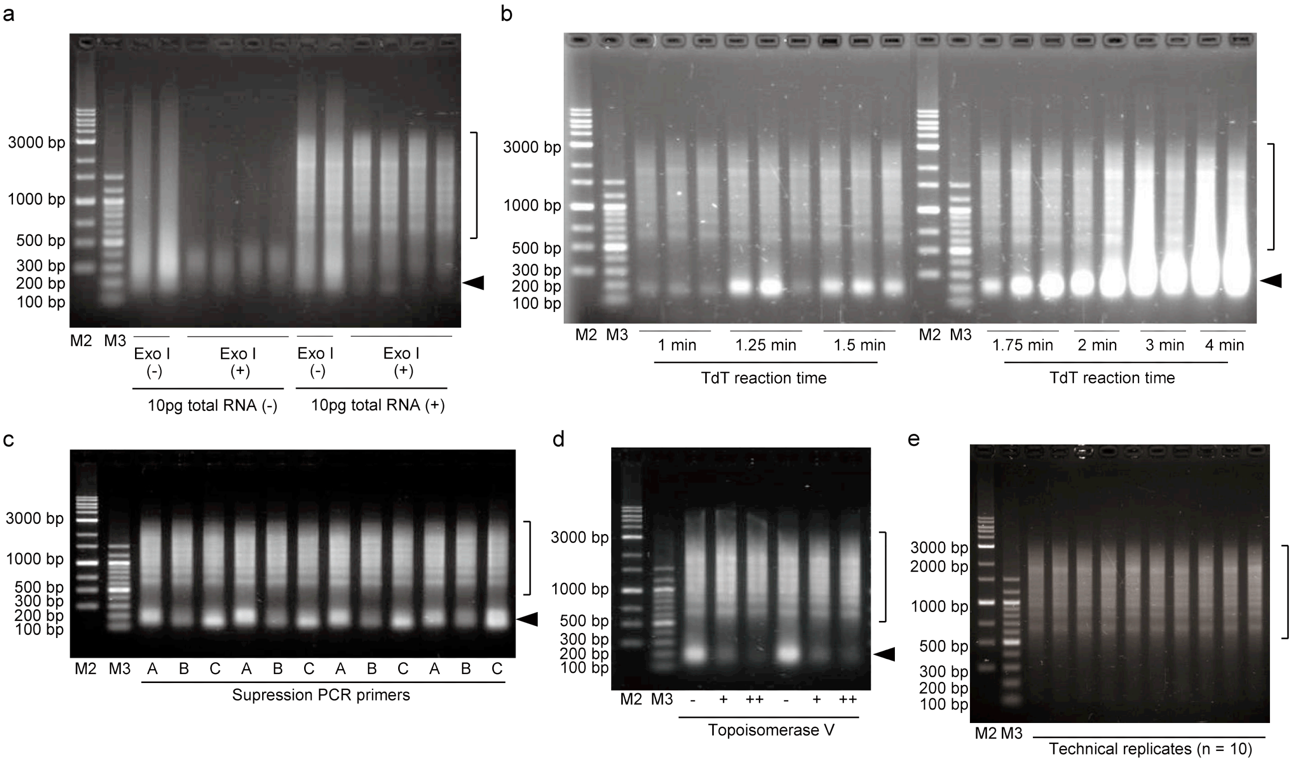

Figure S3 Key steps for robust suppression of byproducts

(a) Digestion of RT primer through the use of exonuclease I: “Exo 1 (+)”, with exonuclease I treatment; “Exo 1 (-)”, without exonuclease I treatment. **(b)** Reaction time of poly-A-tailing by terminal transferase (TdT) affected the synthesis of byproducts. **(c)** The effect of the suppression PCR primers: A primer (TATAGAATTCGCGGCCGCTCGCGAT), B primer (GTATAGAATTCGCGGCCGCTCGCGAT), and C primer (CTATAGAATTCGCGGCCGCTCGCGAT). **(d)** Optional parameter for the suppression of the synthesis of byproducts. Topoisomerase V suppressed the production of byproducts. We added topoisomerase V (ThermoFidelase I) to each reaction as follows: “-”, none; “+”, 1 unit; and “++”, 2 units. The black arrowheads represent the byproducts. **(e)** Three parameters completely suppressed the synthesis of byproducts: exonuclease I treatment, restricted TdT reaction time (50 sec) and suppression PCR primer (B primer). The arrowheads represent the byproducts. The square bracket represents the amplified cDNA.

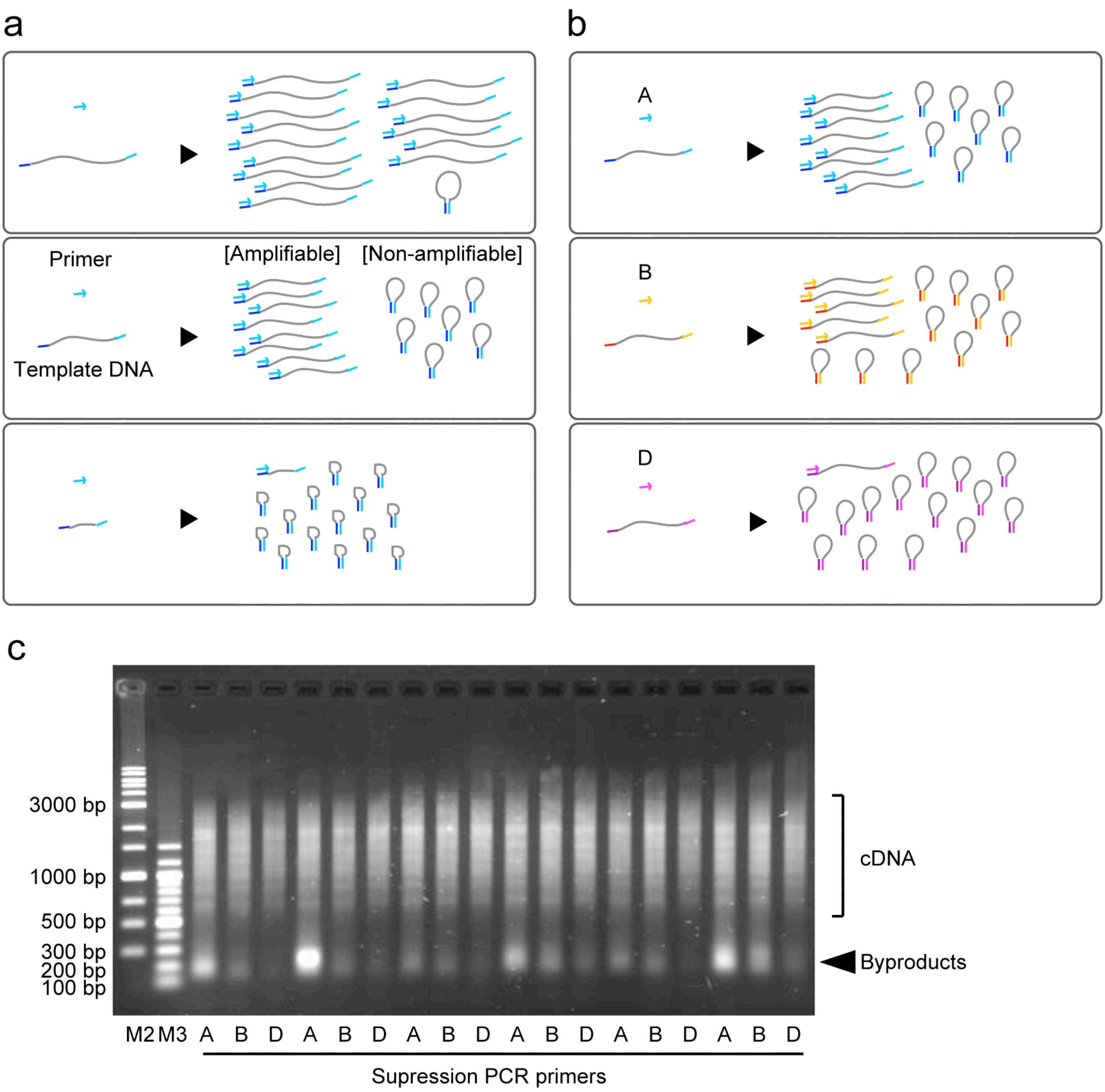

Figure S4 Optimization of suppression PCR for Quartz-Seq.
(a, b) Schematic representation of the effects of suppression PCR. **(a)** The template DNA has complementary sequences at both ends (blue lines and sky-blue lines). These complementary sequences can bind each other. The PCR primer (sky-blue arrows) also binds one end of the template DNA (blue line). The self-bound template DNA forms a “pan-like” structure, and the DNA is not amplified by PCR. The self-binding between the ends of the template DNA competes with the binding between the PCR primer and one end of the template DNA (sky-blue line). If the template DNA is short, one end of the template DNA (blue line) tends to quickly bind the other end of the template DNA (sky-blue line) rather than the PCR primer. If the template DNA is long, one end of the template DNA (blue line) tends to quickly bind the PCR primer rather than the other end of the template DNA (sky-blue line). **(b)** The suppression PCR effect depends on the features of the complementary sequence ends (e.g., cDNA length and GC content). **(c)** We tested three types of suppression PCR primers for the amplification of 10 pg of total RNA. We synthesized byproducts on purpose to evaluate the suppression PCR effect of each primer. The suppression PCR primers were as follows: A primer (TATAGAATTCGCGGCCGCTCGCGAT), B primer

(GTATAGAATTCGCGGCCGCTCGCGAT), and D primer (TGTATAGAATTCGCGGCCGCTCGCGAT). The B and D primers efficiently suppressed byproduct synthesis compared with the A primer. However, the production of cDNA with a size of approximately 500 bp was slightly decreased under the D primer condition compared with the A and B primer conditions. We used the B primer for Quartz-Seq.

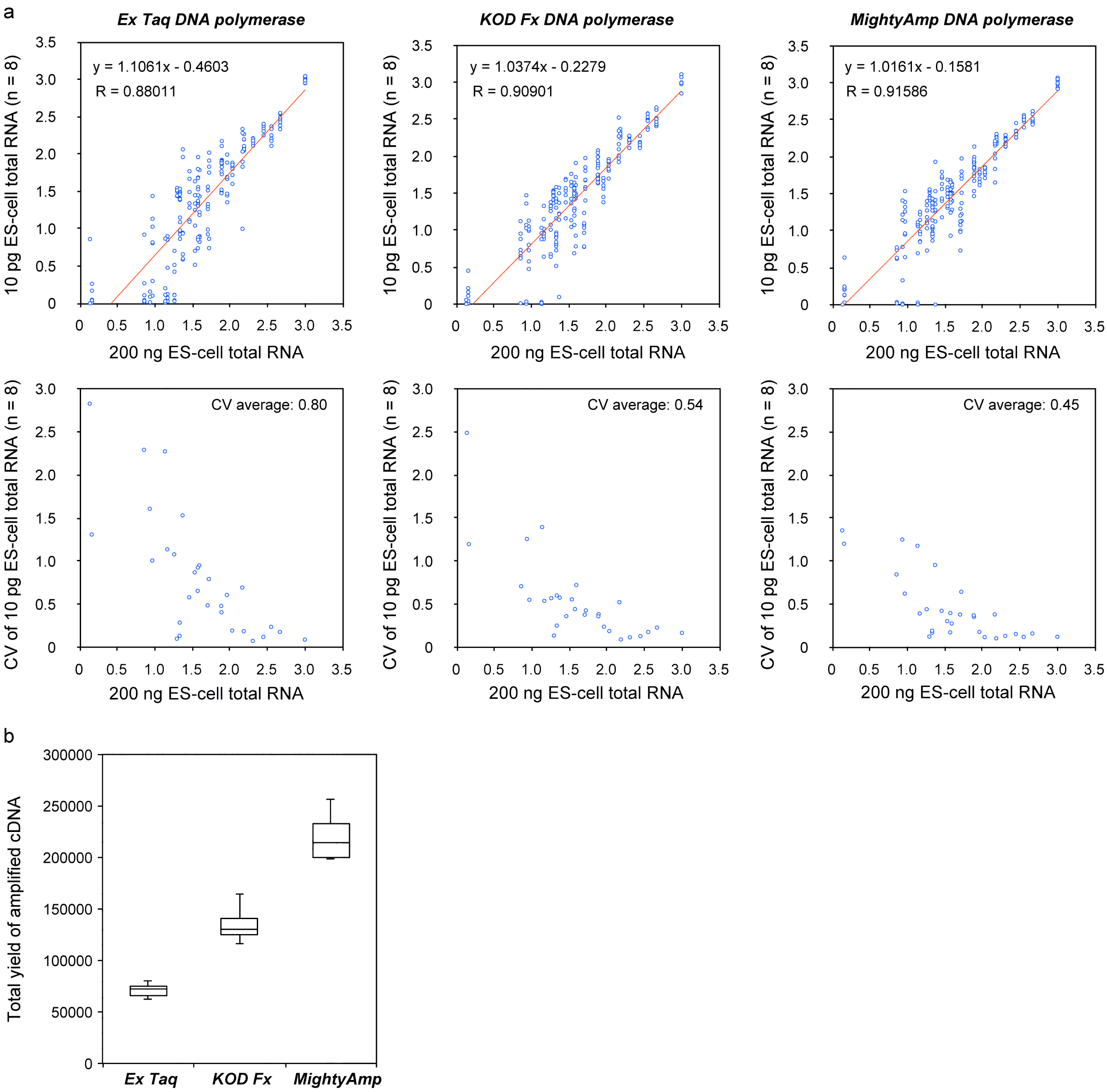

Figure S5 Optimal DNA polymerase for whole-transcript amplification.
The amplified cDNA from 10 pg of total ES-cell (EB5) RNA with spike RNAs (1000, 100, 20 and 5 copies of *Lys*, *Dap*, *Phe,* and *Thr*, respectively, per 10 pg of total RNA) was prepared using the Ex Taq DNA polymerase, the KOD Fx DNA polymerase and the MightyAmp DNA polymerase. The PCR cycle number was 16. After the PCR enrichment, the amplified cDNA was purified with 50 μl of EB buffer. **(a)** The non-WTA sample from 200 ng of total ES-cell (EB5) RNA with spike RNAs was prepared by reverse-transcription using SuperScript III and Oligo-dT primers. We obtained eight technical replicates in different DNA polymerase conditions. We detected the endogenous expression of 26 genes and the 4 spike RNAs using the amplified cDNA and the non-WTA samples. The expression level of each gene was normalized with the qPCR quantity of the spike RNAs in the non-WTA sample. All of the expression levels were translated into copy numbers. The X- and Y-axes represent the log10(copy+1). The detected genes were the following (in order of decreasing expression level): *Lys*, *Trim28*, *Ywhae*, *Sox2*, *Dppa5*, *Prdx1*, *Lefty1*, *Actb*, *Nanog*, *Dap*, *Dnmt1*, *Eras*, *Pou5f1*, *Dppa3*, *Alpl*, *Tial1*, *Ezh2*, *Fgf4*, *Ifitm3*, *Gapdh*, *Phe*, *Ehmt2*, *Nodal*, *Tbp*, *Foxh1*, *Dnmt3b*, *Thr*, *Tyk2*, *Jak1,* and *Gata6*. The results of eight independent amplifications (from

10 pg of total RNA) are plotted against the non-WTA control (200 ng). **(b)** The box plots show the total yield of the amplified cDNA using the sum of the qPCR quantity of 30 genes.

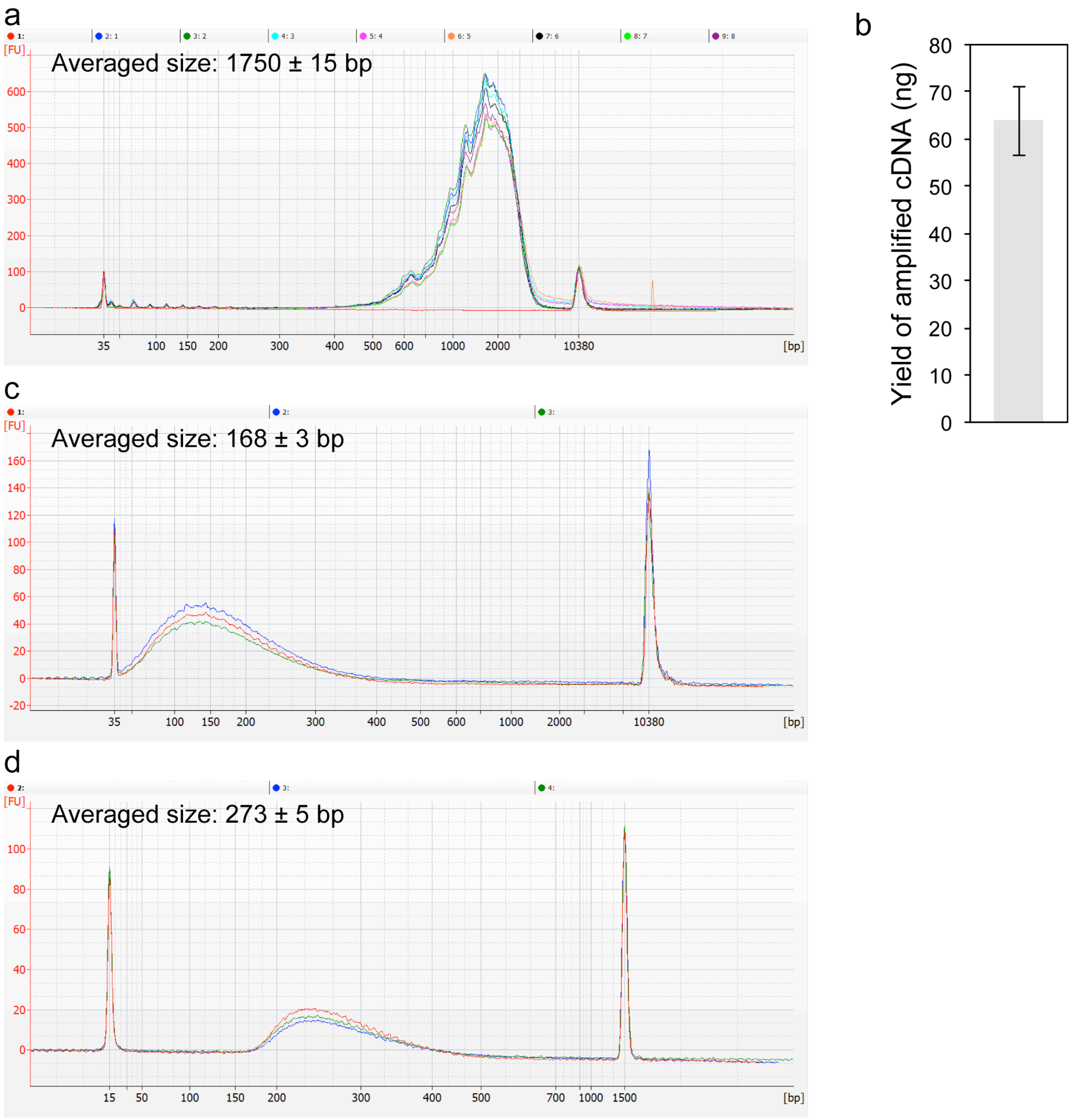

Figure S6 Quality check of the library preparation for single-cell Quartz-Seq.
(a) The amplified cDNAs were obtained from diluted 10 pg of total RNA. The PCR cycle number was 21. The distribution of the amplified cDNA was analyzed using the Bioanalyzer High Sensitivity DNA Kit (amplified sample, n = 8; blank, n = 1). The amplified cDNA size ranged from approximately 400 bp to 4000 bp. **(b)** The total yield of amplified cDNA was quantified using PicoGreen (n = 8, 63.8 ± 7.2 ng). **(c)** The shearing DNA from the amplified DNA (n = 3) was analyzed using the Bioanalyzer High Sensitivity DNA Kit. **(d)** The DNA sequencing library from the amplified DNA (n = 3) was analyzed using the Bioanalyzer DNA 1000 Kit.

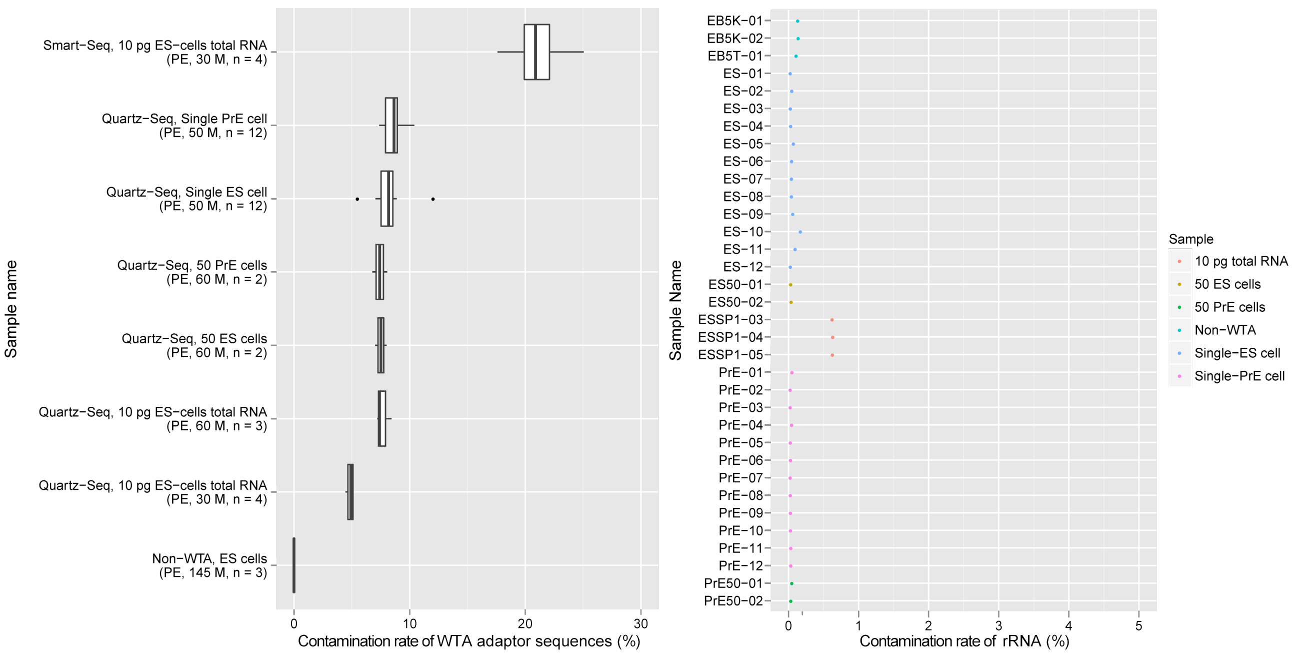

Figure S8 Percentage of sequence reads of the suppression PCR primer or rRNA.
The left panel shows the contamination rate of the sequence read from the WTA adaptor sequences. The X-axis indicates the contamination percentage of all sequence reads. The right panel shows the contamination rate of rRNA. The X-axis indicates the percentage of the rRNA contamination in all sequence reads.

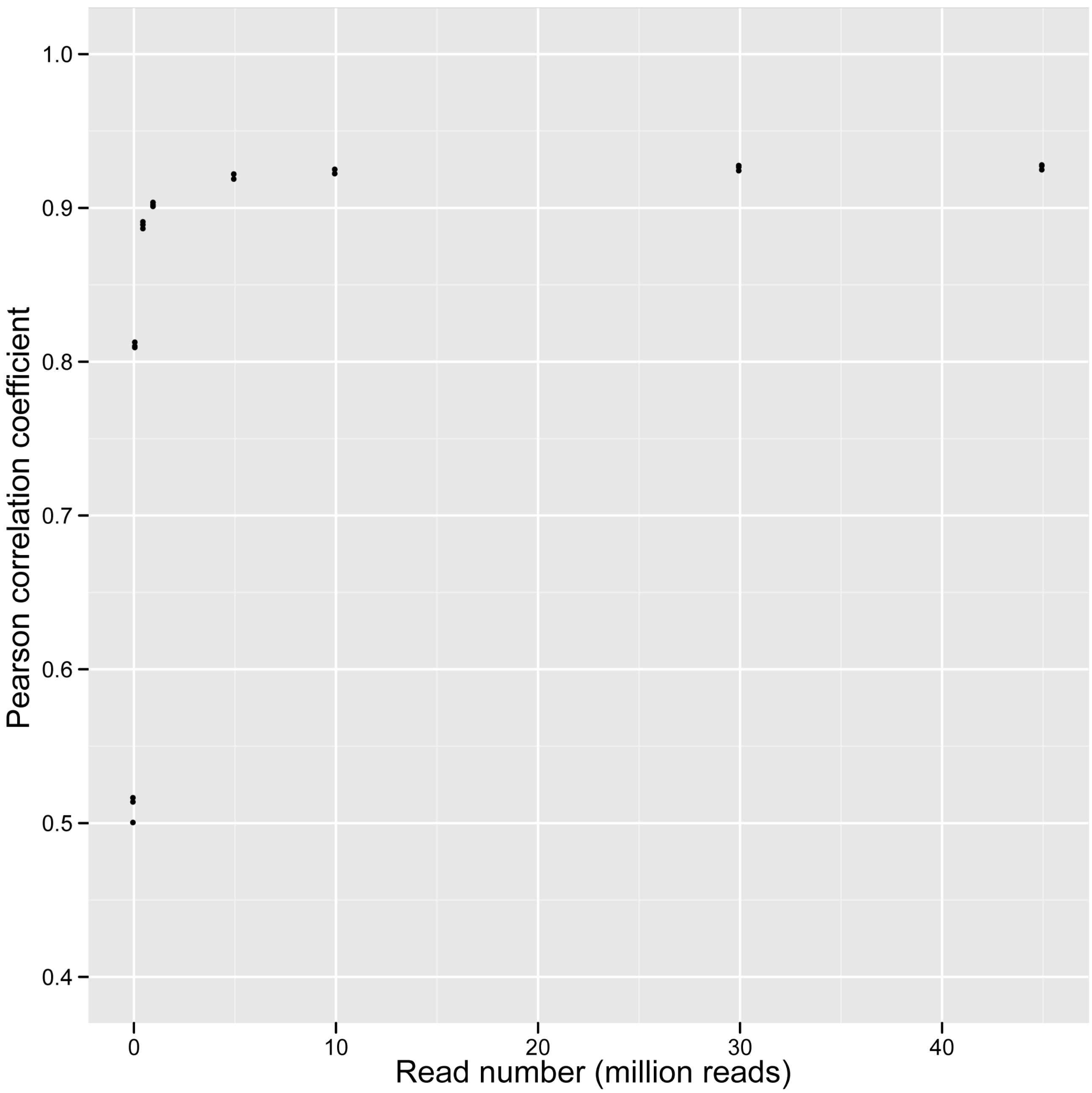

Figure S9 Relationship between the read number and the reproducibility.
The down sampling of the sequence reads of single-cell Quartz-Seq. The X-axis indicates the read number (in million reads) of Quartz-Seq. The Y-axis is the Pearson correlation between the technical replications of independent amplifications (n = 3).

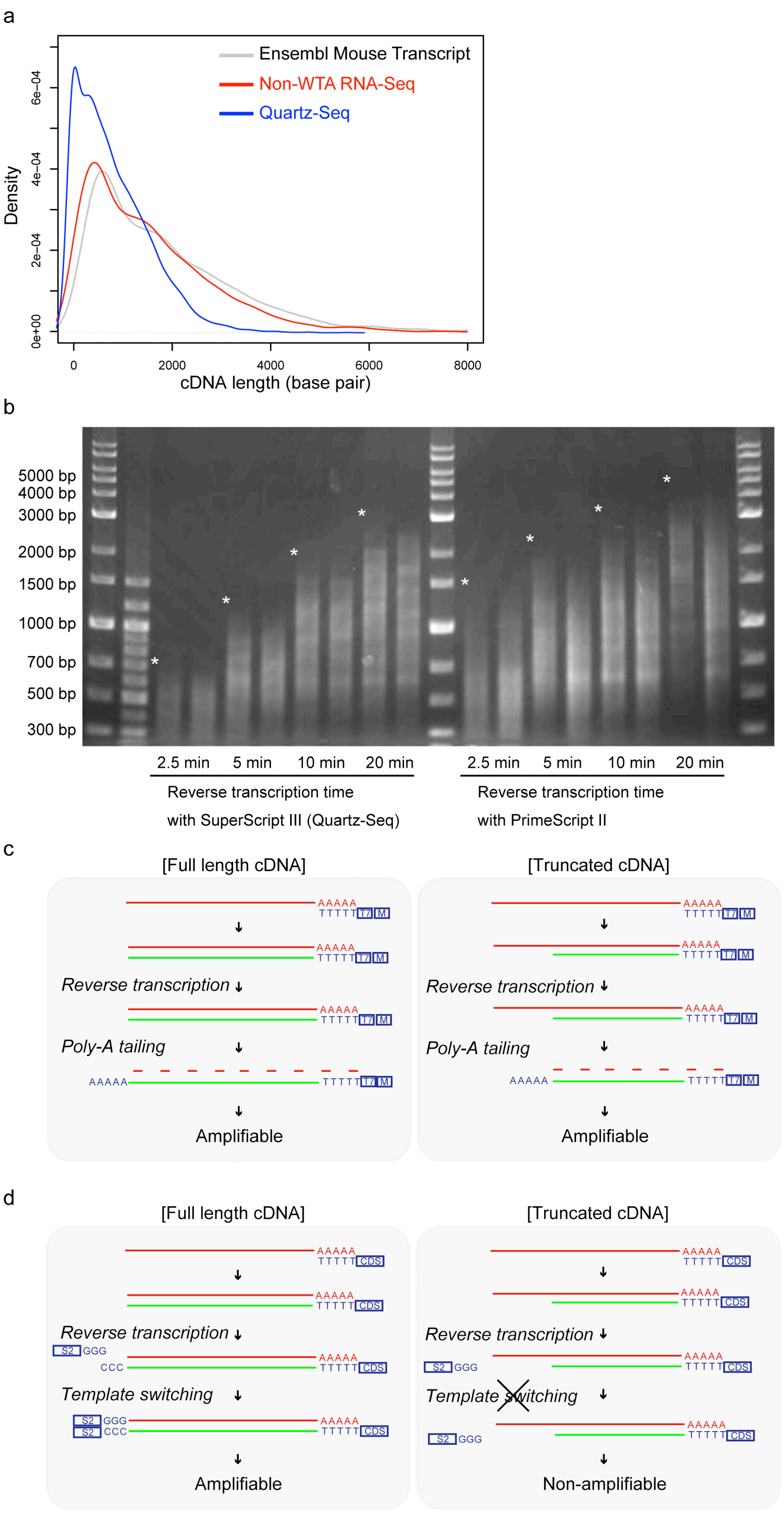
a
Ensembl Mouse Transcript
Non-WTA RNA-Seq
Quartz-Seq
Density
0e+00
2e−04
4e−04
6e−04
0
2000
4000
6000
8000
cDNA length (base pair)
b
5000 bp
4000 bp
3000 bp
2000 bp
1500 bp
1000 bp
700 bp
500 bp
300 bp
2.5 min
5 min
10 min
20 min
Reverse transcription time
with SuperScript III (Quartz-Seq)
2.5 min
5 min
10 min
20 min
Reverse transcription time
with PrimeScript II
c
[Full length cDNA]
[Truncated cDNA]
AAAAA
TTTTT
T7
M
Reverse transcription
Poly-A tailing
Amplifiable
Amplifiable
d
[Full length cDNA]
[Truncated cDNA]
AAAAA
TTTTT
CDS
S2
GGG
CCC
Reverse transcription
Template switching
Amplifiable
Non-amplifiable

Figure S10 Optimization of cDNA length in the technical development of single-cell Quartz-Seq.

(a) We detected 5,929 transcripts that were reproducibly expressed in single-cell Quartz-Seq with 10 pg of total RNA from an ES cell (FPKM ≥ 10). The Full-length Ensembl Mouse Transcript was expressed (FPKM ≥ 10) in the ES cell (gray line). The red line shows the cDNA length produced by conventional RNA-Seq. The blue line shows the cDNA length produced by single-cell Quartz-Seq using 10 pg of total RNA. The X-axis is the cDNA length (base pairs), and the Y-axis shows the density of the number of genes. **(b)** We amplified cDNA using 10 pg of mouse ES total RNA as the starting material. In the reverse-transcription step of Quartz-Seq, we used SuperScript III (Life Technologies) or PrimeScript II (TaKaRa-Clontech). The asterisks represent the maximum size of almost all the amplified cDNAs. The size of the cDNA amplified with SuperScript III (20 min) was less than 3,000 bp, but that amplified with PrimeScript II (20 min) was greater than 3,000-4,000 bp. To avoid PCR-bias for long cDNA, we used SuperScript III and intentionally restricted the reverse-transcription time (20 min). **(c)** Schematic representation of the poly-A tailing reaction for reverse-transcribed cDNA. The poly-A tailing reaction targets both full-length and truncated cDNAs. Quartz-Seq is based on the poly-A tailing reaction. **(d)** The schematic representation of the template switching reaction for reverse-transcribed cDNA. The 5’ end of the completed transcribed cDNA was modified by the terminal transferase activity of reverse transcriptase. The template switching reaction is enriched in the 5’ ends of the modified cDNA. However, the template switching reaction is suppressed in the truncated cDNA. Smart-Seq is based on a template-switching reaction.

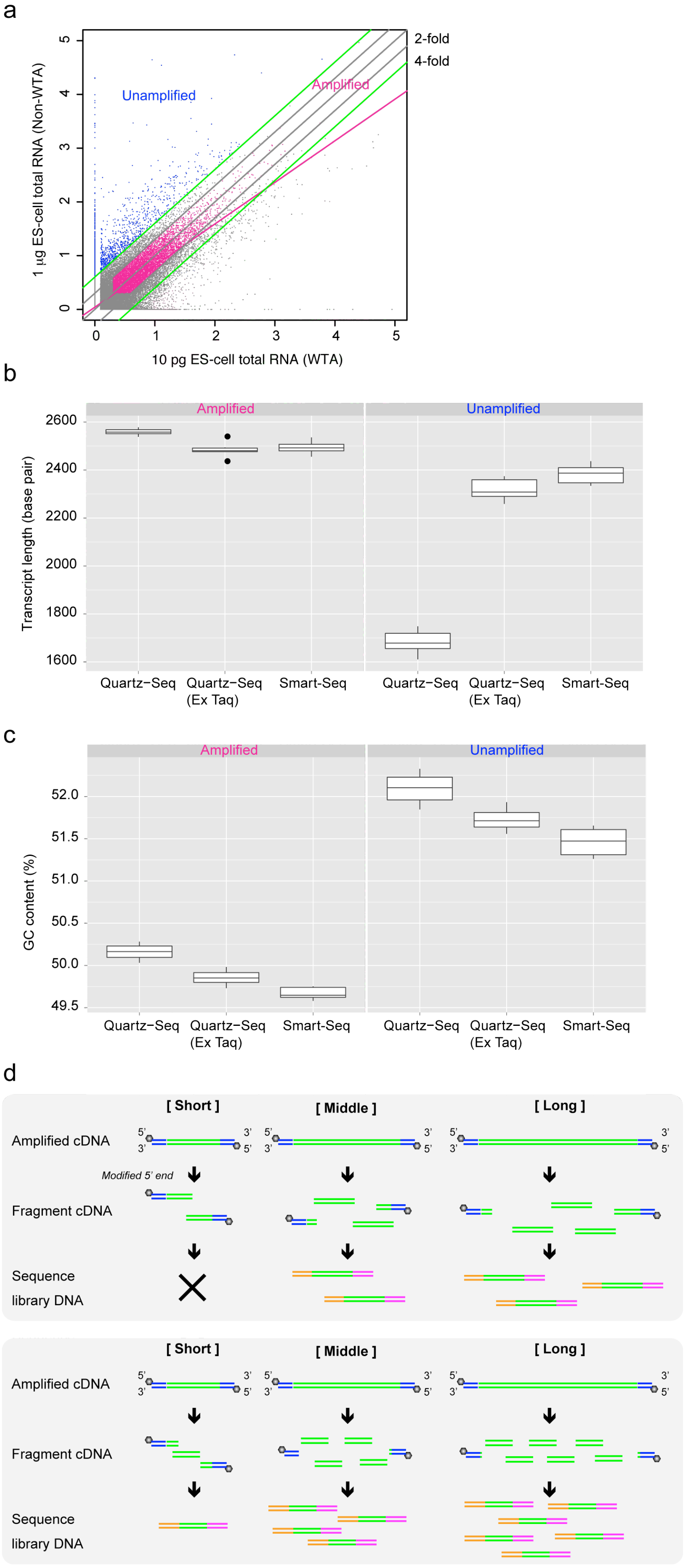
a
Unamplified
Amplified
2-fold
4-fold
1 µg ES-cell total RNA (Non-WTA)
10 pg ES-cell total RNA (WTA)
b
Amplified
Unamplified
Transcript length (base pair)
Quartz−Seq
Quartz−Seq (Ex Taq)
Smart-Seq
c
Amplified
Unamplified
GC content (%)
Quartz−Seq
Quartz−Seq (Ex Taq)
Smart-Seq
d
[Short]
[Middle]
[Long]
Amplified cDNA
Modified 5' end
Fragment cDNA
Sequence library DNA
[Short]
[Middle]
[Long]
Amplified cDNA
Fragment cDNA
Sequence library DNA

Figure S11 Trend of unamplified isoforms for each single-cell RNA-Seq method.
We compared the conventional RNA-Seq method with each single-cell RNA-Seq method. We prepared the Quartz-Seq data (n=4), Smart-Seq data (n=4), and modified Quartz-Seq data (n=3, Quartz-Seq [Ex Taq]) using 10 pg of the total RNA from the ES cells. We used Ex Taq DNA polymerase (TaKaRa) instead of MightyAmp DNA polymerase in the modified Quartz-Seq. Ex Taq DNA polymerase was used in previous methods such as those of Kurimoto et al. and Tang et al. **(a)** Scatter plot comparing conventional RNA-Seq with single-cell RNA-Seq. The X-axis represents the isoform expression from each single-cell RNA-Seq method, and the Y-axis represents the isoform expression from a conventional RNA-Seq. The red points (amplified) indicate isoforms with an FPKM expression larger than 1.0 and exhibiting a less than two-fold expression change between technical duplicates. The blue points (unamplified) indicate isoforms with an FPKM larger than 1.0 and exhibiting a greater than four-fold expression change. **(b)** Using the Ensembl Mouse Transcript database, we calculated the full-length sizes of the amplified and unamplified isoforms for each method. **(c)** We calculated the average GC content of the amplified and unamplified isoforms for each method. **(d)** Schematic representation of the relationship between cDNA length and sequence library DNA. Amplified cDNA has a WTA adaptor sequence at both ends (Blue lines). The WTA adaptor was modified by an amination or another modification at the 5' end (hexagon). These 5' modifications block the ligation between the cDNA fragments using a WTA adaptor and sequence adaptors (orange lines and purple lines). The upper panel indicates the fragmentation effect in weak fragmentation conditions. The lower panel indicates the fragmentation effect in strong fragmentation conditions. In general, it is more difficult to convert short cDNA to sequence library DNA than long cDNA.

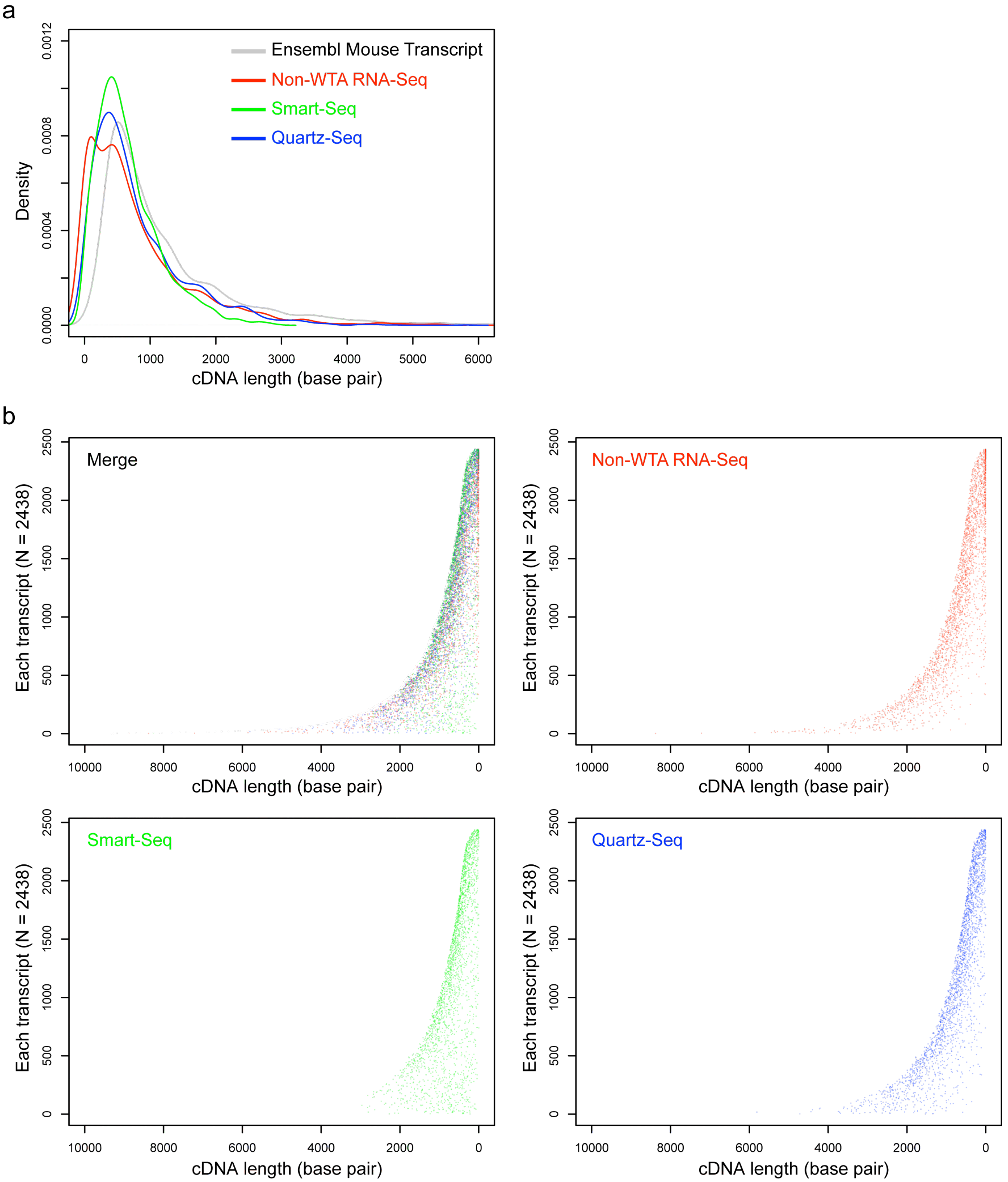

Figure S12 Amplified cDNA lengths resulting from single-cell RNA-Seq methods. We compared the cDNA length between Quartz-Seq and Smart-Seq using 10 pg of total RNA. **(a)** We detected 2,438 transcripts that were reproducibly expressed in single-cell Smart-Seq with 10 pg of total RNA from ES cells (FPKM ≥ 10). We used the 2,438 transcripts for the subsequent evaluation. We calculated the cDNA length for each method using the 2,438 transcripts. The gray line represents the full-length cDNAs from the Ensembl Mouse Transcript database. The red line shows the cDNA lengths from non-WTA RNA-Seq with 1 μg of ES total RNA. The green line shows the cDNA lengths from Smart-Seq with 10 pg of ES total RNA. The blue line shows the cDNA lengths from Quartz-Seq with 10 pg of ES total RNA. The X-axis is the cDNA length (base pairs), and the Y-axis shows the density of the number of transcripts. The cDNA length from Quartz-Seq was not different from that of Smart-Seq and may be slightly better than that of Smart-Seq for all ranges studied. However, the cDNA lengths from Smart-Seq were slightly better than those from

Quartz-Seq in the 500 to 1,000 bp range. The cDNA lengths from Quartz-Seq were better than those from Smart-Seq in the >1,500 bp range. **(b)** We plotted each transcript (from the 2,438 transcripts) with respect to cDNA length. The X-axis is the cDNA length (base pairs). Each dot represents a transcript, which is aligned along the Y-axis according to the full length of its corresponding Ensembl Mouse Transcript.

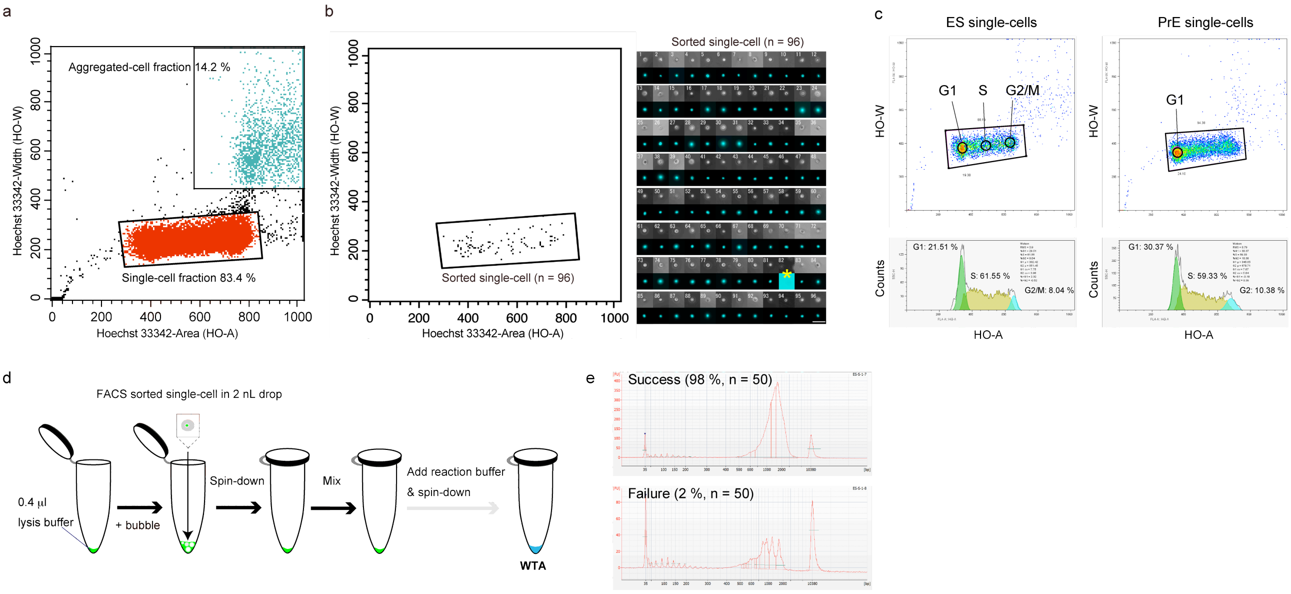

Figure S13 Success rate of whole-transcript amplification from single cells sorted by fluorescence-activated cell sorting (FACS).

(a) The cultured cells were fractionated into single-cell fractions (red dots, 83.4 %) and aggregated-cell fractions (green dots, 14.2 %) by FACS flow. **(b)** Fidelity of single-cell sorting by FACS. We sorted a single cell to a 10-μl PBS drop in 96-well microplate from the single-cell fraction; this separation was confirmed by microscopic observation. The yellow asterisk represents a dried-out well. We confirmed that the sorted cells from the single-cell fraction were in fact single cells (100 %, n = 95, the dried-out well was not included in the analysis). **(c)** Single-cell sorting using ES cells or PrE cells. Single cells were sorted from each phase of the cell-cycle according to the Hoechst 33342 area. The data analysis was performed using the FlowJo software. The percentages of cells in the different cell-cycle phases were estimated using the Watson Pragmatic model. **(d)** Schematic representation of the single-cell collection using FACS-sorting. The volume of the droplet with a single-cell was approximately 2-3 nl. For expansion of drop area, just before single-cell collection, we added air-bubbles to the single-cell lysis buffer using a single-channel micropipette or a multi-channel micropipette. After that we collected single-cell to lysis buffer. **(e)** Success rate of the amplification: 98 % of the single-cell amplification (n = 49) was successful. Typical distribution of the resulting cDNA, which was analyzed using a Bioanalyzer High-Sensitivity Kit.

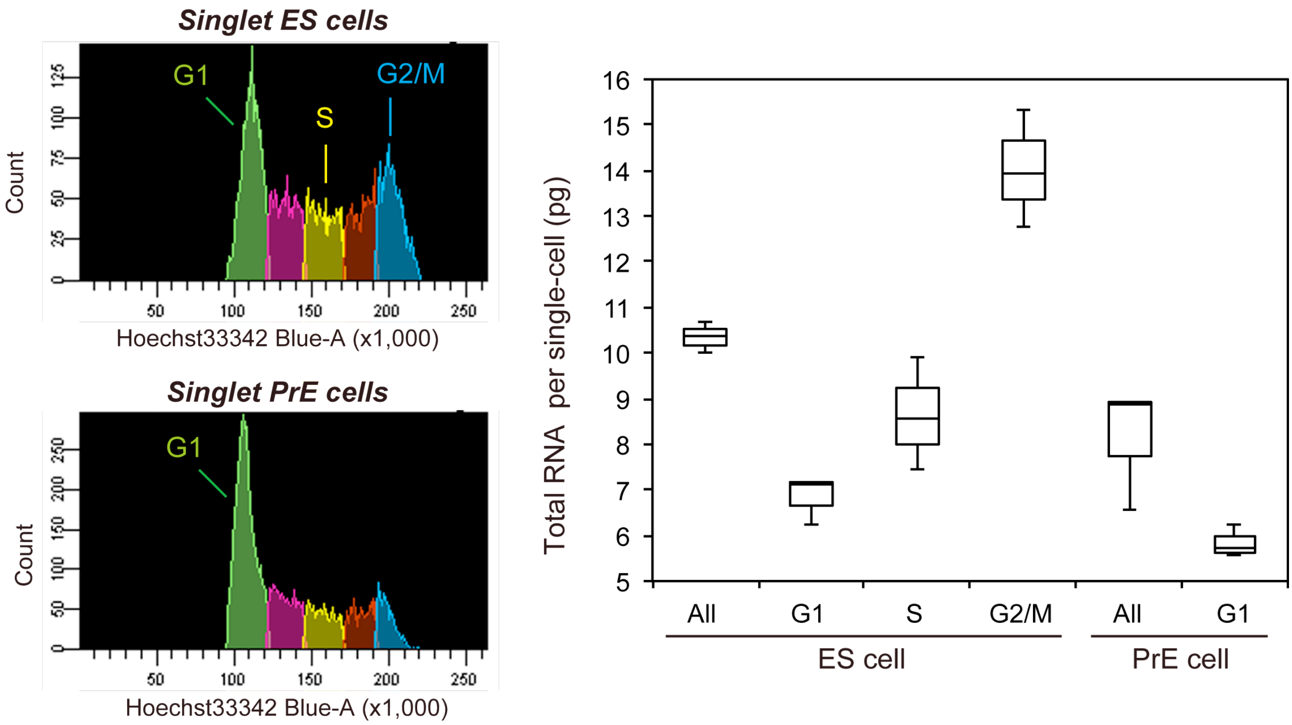

Figure S14 Amount of total RNA from a single-cell at each cell-cycle phase.
The single-cell fraction was split into five cell-cycle phases (G1, eS: early S, S, lS: late S, and G2/M) according to the Hoechst 33342-Area. The total RNA was purified from 50,000 cells in each phase of the cell cycle (n = 3). The amount of total RNA from a single-cell was estimated by RiboGreen (Invitrogen). ES: All, 10.3 ± 0.4 pg/cell; G1, 6.8 ± 0.5 pg/cell; S, 8.6 ± 1.2 pg/cell; G2/M, 14.0 ± 1.2 pg/cell. PrE: All, 8.1 ± 1.3 pg/cell; G1, 5.8 ± 0.3 pg/cell.

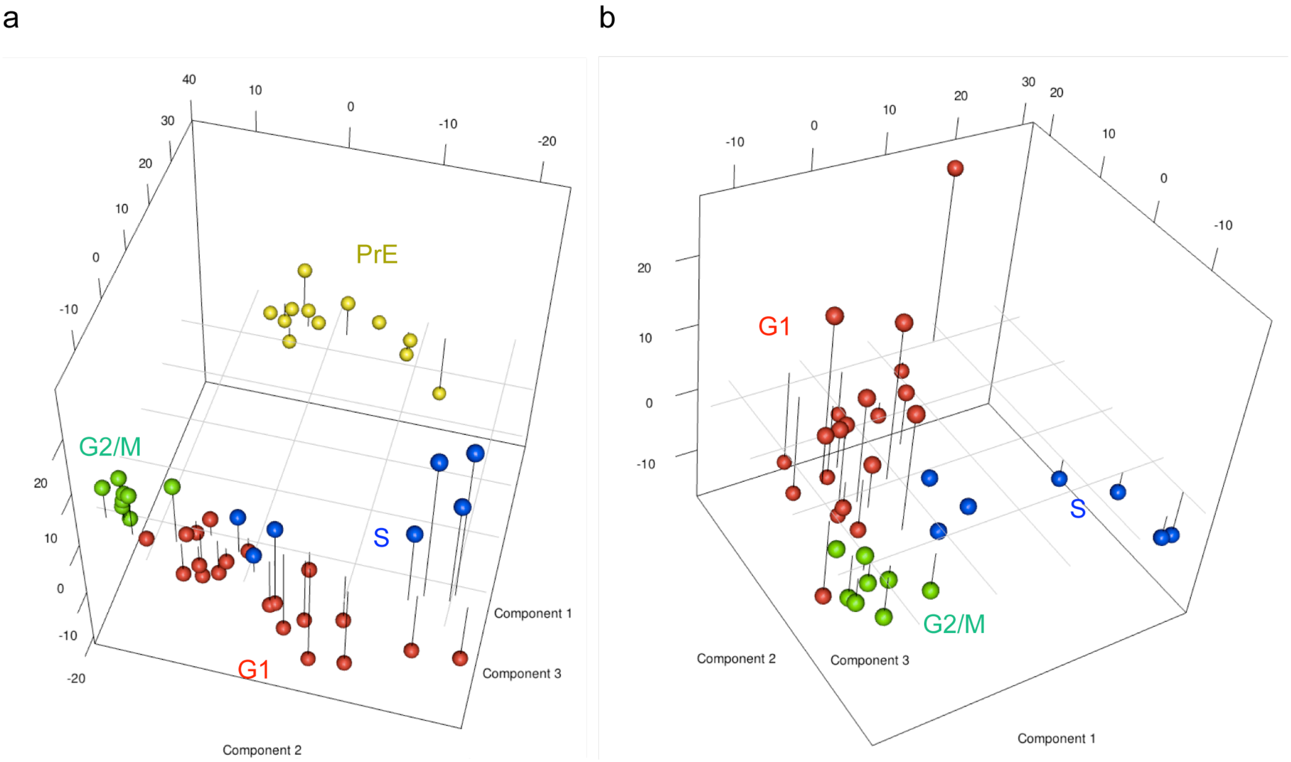

Figure S15 Principal component analysis (PCA) of single-cells from different cell types at different cell-cycle phases.

(a) PCA with single ES (G1, S, and G2/M phases) and PrE (G1 phase) cells. **(b)** PCA with single ES cells at differently cell-cycle phases. The differently colored dots represent the following: ES G1, red dots; ES S, blue dots; ES G2/M, green dots; PrE single cells, yellow dots. The X-, Y- and Z-axes represent the principal component 1, the principal component 2, and the principal component 3, respectively.

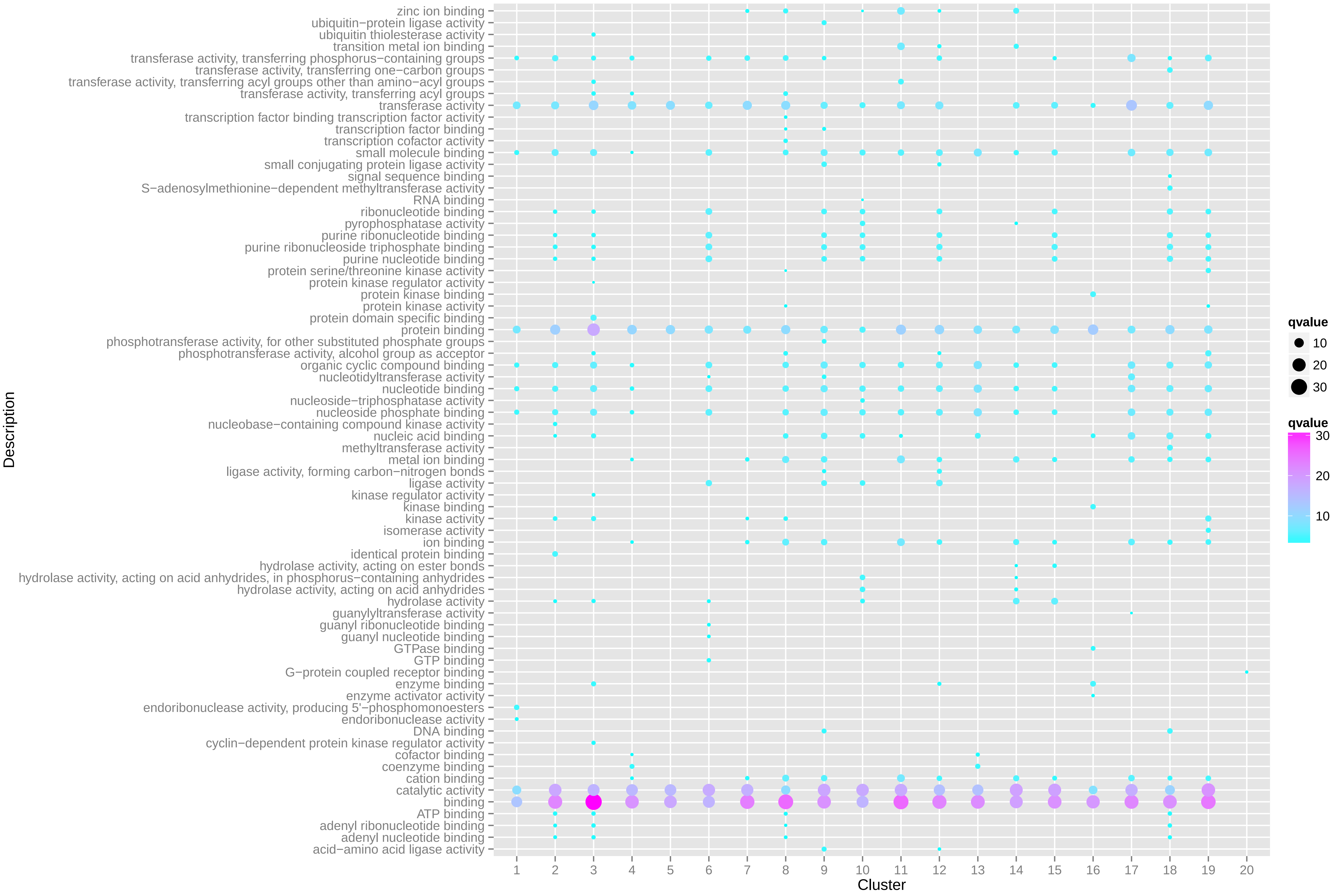

zinc ion binding
ubiquitin−protein ligase activity
ubiquitin thiolesterase activity
transition metal ion binding
transferase activity, transferring phosphorus−containing groups
transferase activity, transferring one−carbon groups
transferase activity, transferring acyl groups other than amino−acyl groups
transferase activity, transferring acyl groups
transferase activity
transcription factor binding transcription factor activity
transcription factor binding
transcription cofactor activity
small molecule binding
small conjugating protein ligase activity
signal sequence binding
S−adenosylmethionine−dependent methyltransferase activity
RNA binding
ribonucleotide binding
pyrophosphatase activity
purine ribonucleotide binding
purine ribonucleoside triphosphate binding
purine nucleotide binding
protein serine/threonine kinase activity
protein kinase regulator activity
protein kinase binding
protein kinase activity
protein domain specific binding
protein binding
phosphotransferase activity, for other substituted phosphate groups
phosphotransferase activity, alcohol group as acceptor
organic cyclic compound binding
nucleotidyltransferase activity
nucleotide binding
nucleoside−triphosphatase activity
nucleoside phosphate binding
nucleobase−containing compound kinase activity
nucleic acid binding
methyltransferase activity
metal ion binding
ligase activity, forming carbon−nitrogen bonds
ligase activity
kinase regulator activity
kinase binding
kinase activity
isomerase activity
ion binding
identical protein binding
hydrolase activity, acting on ester bonds
hydrolase activity, acting on acid anhydrides, in phosphorus−containing anhydrides
hydrolase activity, acting on acid anhydrides
hydrolase activity
guanylyltransferase activity
guanyl ribonucleotide binding
guanyl nucleotide binding
GTPase binding
GTP binding
G−protein coupled receptor binding
enzyme binding
enzyme activator activity
endoribonuclease activity, producing 5'−phosphomonoesters
endoribonuclease activity
DNA binding
cyclin−dependent protein kinase regulator activity
cofactor binding
coenzyme binding
cation binding
catalytic activity
binding
ATP binding
adenyl ribonucleotide binding
adenyl nucleotide binding
acid−amino acid ligase activity
Description
1 2 3 4 5 6 7 8 9 10 11 12 13 14 15 16 17 18 19 20
Cluster
qvalue
10
20
30
qvalue
30
20
10

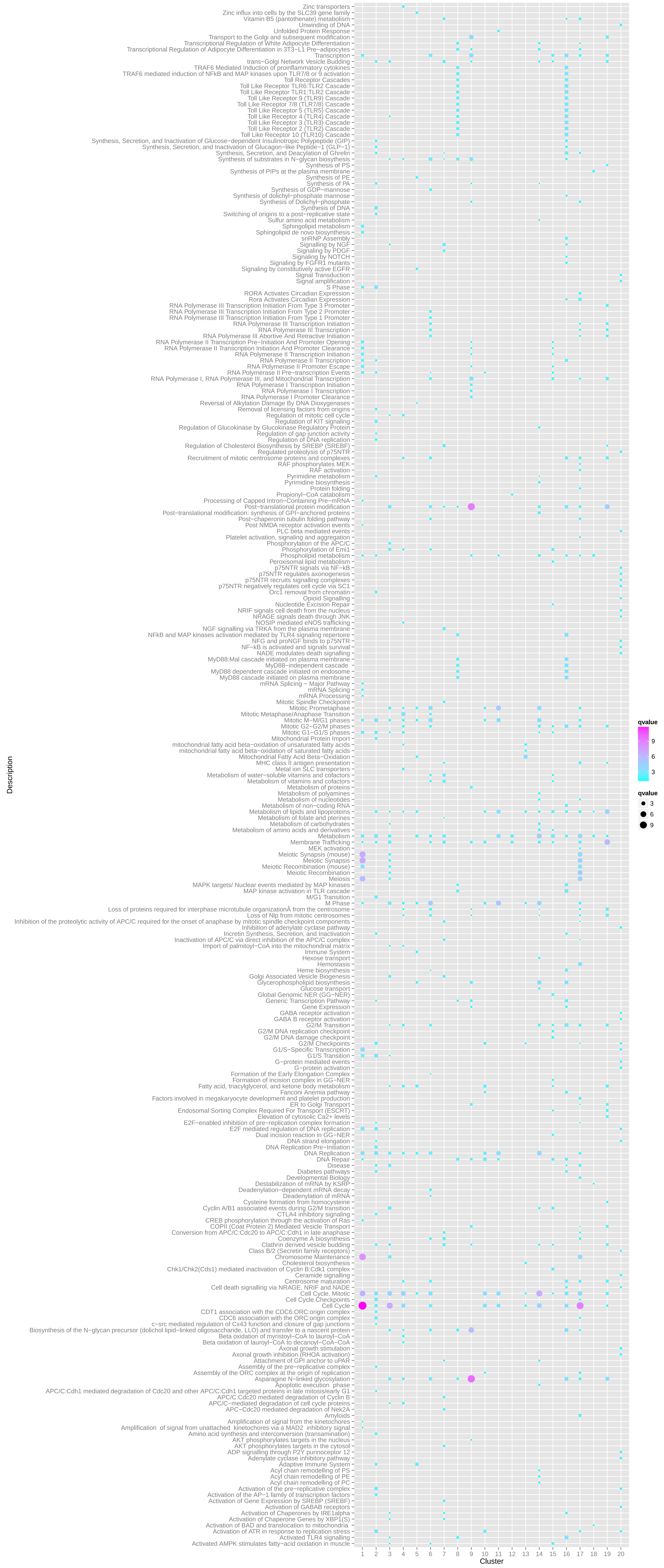

Zinc transporters
Zinc influx into cells by the SLC39 gene family
Vitamin B5 (pantothenate) metabolism
Unwinding of DNA
Unfolded Protein Response
Transport to the Golgi and subsequent modification
Transcriptional Regulation of White Adipocyte Differentiation
Transcriptional Regulation of Adipocyte Differentiation in 3T3−L1 Pre−adipocytes
Transcription
trans−Golgi Network Vesicle Budding
TRAF6 Mediated Induction of proinflammatory cytokines
TRAF6 mediated induction of NFkB and MAP kinases upon TLR7/8 or 9 activation
Toll Receptor Cascades
Toll Like Receptor TLR6:TLR2 Cascade
Toll Like Receptor TLR1:TLR2 Cascade
Toll Like Receptor 9 (TLR9) Cascade
Toll Like Receptor 7/8 (TLR7/8) Cascade
Toll Like Receptor 5 (TLR5) Cascade
Toll Like Receptor 4 (TLR4) Cascade
Toll Like Receptor 3 (TLR3) Cascade
Toll Like Receptor 2 (TLR2) Cascade
Toll Like Receptor 10 (TLR10) Cascade
Synthesis, Secretion, and Inactivation of Glucose−dependent Insulinotropic Polypeptide (GIP)
Synthesis, Secretion, and Inactivation of Glucagon−like Peptide−1 (GLP−1)
Synthesis, Secretion, and Deacylation of Ghrelin
Synthesis of substrates in N−glycan biosythesis
Synthesis of PS
Synthesis of PIPs at the plasma membrane
Synthesis of PE
Synthesis of PA
Synthesis of GDP−mannose
Synthesis of dolichyl−phosphate mannose
Synthesis of Dolichyl−phosphate
Synthesis of DNA
Switching of origins to a post−replicative state
Sulfur amino acid metabolism
Sphingolipid metabolism
Sphingolipid de novo biosynthesis
snRNP Assembly
Signalling by NGF
Signaling by PDGF
Signaling by NOTCH
Signaling by FGFR1 mutants
Signaling by constitutively active EGFR
Signal Transduction
Signal amplification
S Phase
RORA Activates Circadian Expression
Rora Activates Circadian Expression
RNA Polymerase III Transcription Initiation From Type 3 Promoter
RNA Polymerase III Transcription Initiation From Type 2 Promoter
RNA Polymerase III Transcription Initiation From Type 1 Promoter
RNA Polymerase III Transcription Initiation
RNA Polymerase III Transcription
RNA Polymerase III Abortive And Retractive Initiation
RNA Polymerase II Transcription Pre−Initiation And Promoter Opening
RNA Polymerase II Transcription Initiation And Promoter Clearance
RNA Polymerase II Transcription Initiation
RNA Polymerase II Transcription
RNA Polymerase II Promoter Escape
RNA Polymerase II Pre−transcription Events
RNA Polymerase I, RNA Polymerase III, and Mitochondrial Transcription
RNA Polymerase I Transcription Initiation
RNA Polymerase I Transcription
RNA Polymerase I Promoter Clearance
Reversal of Alkylation Damage By DNA Dioxygenases
Removal of licensing factors from origins
Regulation of mitotic cell cycle
Regulation of KIT signaling
Regulation of Glucokinase by Glucokinase Regulatory Protein
Regulation of gap junction activity
Regulation of DNA replication
Regulation of Cholesterol Biosynthesis by SREBP (SREBF)
Regulated proteolysis of p75NTR
Recruitment of mitotic centrosome proteins and complexes
RAF phosphorylates MEK
RAF activation
Pyrimidine metabolism
Pyrimidine biosynthesis
Protein folding
Propionyl−CoA catabolism
Processing of Capped Intron−Containing Pre−mRNA
Post−translational protein modification
Post−translational modification: synthesis of GPI−anchored proteins
Post−chaperonin tubulin folding pathway
Post NMDA receptor activation events
PLC beta mediated events
Platelet activation, signaling and aggregation
Phosphorylation of the APC/C
Phosphorylation of Emi1
Phospholipid metabolism
Peroxisomal lipid metabolism
p75NTR signals via NF−kB
p75NTR regulates axonogenesis
p75NTR recruits signalling complexes
p75NTR negatively regulates cell cycle via SC1
Orc1 removal from chromatin
Opioid Signalling
Nucleotide Excision Repair
NRIF signals cell death from the nucleus
NRAGE signals death through JNK
NOSIP mediated eNOS trafficking
NGF signalling via TRKA from the plasma membrane
NFkB and MAP kinases activation mediated by TLR4 signaling repertoire
NFG and proNGF binds to p75NTR
NF−kB is activated and signals survival
NADE modulates death signalling
MyD88:Mal cascade initiated on plasma membrane
MyD88−independent cascade
MyD88 dependent cascade initiated on endosome
MyD88 cascade initiated on plasma membrane
mRNA Splicing − Major Pathway
mRNA Splicing
mRNA Processing
Mitotic Spindle Checkpoint
Mitotic Prometaphase
Mitotic Metaphase/Anaphase Transition
Mitotic M−M/G1 phases
Mitotic G2−G2/M phases
Mitotic G1−G1/S phases
Mitochondrial Protein Import
mitochondrial fatty acid beta−oxidation of unsaturated fatty acids
mitochondrial fatty acid beta−oxidation of saturated fatty acids
Mitochondrial Fatty Acid Beta−Oxidation
MHC class II antigen presentation
Metal ion SLC transporters
Metabolism of water−soluble vitamins and cofactors
Metabolism of vitamins and cofactors
Metabolism of proteins
Metabolism of polyamines
Metabolism of nucleotides
Metabolism of non−coding RNA
Metabolism of lipids and lipoproteins
Metabolism of folate and pterines
Metabolism of carbohydrates
Metabolism of amino acids and derivatives
Metabolism
Membrane Trafficking
MEK activation
Meiotic Synapsis (mouse)
Meiotic Synapsis
Meiotic Recombination (mouse)
Meiotic Recombination
Meiosis
MAPK targets/ Nuclear events mediated by MAP kinases
MAP kinase activation in TLR cascade
M/G1 Transition
M Phase
Loss of proteins required for interphase microtubule organizationÂ from the centrosome
Loss of Nlp from mitotic centrosomes
Inhibition of the proteolytic activity of APC/C required for the onset of anaphase by mitotic spindle checkpoint components
Inhibition of adenylate cyclase pathway
Incretin Synthesis, Secretion, and Inactivation
Inactivation of APC/C via direct inhibition of the APC/C complex
Import of palmitoyl−CoA into the mitochondrial matrix
Immune System
Hexose transport
Hemostasis
Heme biosynthesis
Golgi Associated Vesicle Biogenesis
Glycerophospholipid biosynthesis
Glucose transport
Global Genomic NER (GG−NER)
Generic Transcription Pathway
Gene Expression
GABA receptor activation
GABA B receptor activation
G2/M Transition
G2/M DNA replication checkpoint
G2/M DNA damage checkpoint
G2/M Checkpoints
G1/S−Specific Transcription
G1/S Transition
G−protein mediated events
G−protein activation
Formation of the Early Elongation Complex
Formation of incision complex in GG−NER
Fatty acid, triacylglycerol, and ketone body metabolism
Fanconi Anemia pathway
Factors involved in megakaryocyte development and platelet production
ER to Golgi Transport
Endosomal Sorting Complex Required For Transport (ESCRT)
Elevation of cytosolic Ca2+ levels
E2F−enabled inhibition of pre−replication complex formation
E2F mediated regulation of DNA replication
Dual incision reaction in GG−NER
DNA strand elongation
DNA Replication Pre−Initiation
DNA Replication
DNA Repair
Disease
Diabetes pathways
Developmental Biology
Destabilization of mRNA by KSRP
Deadenylation−dependent mRNA decay
Deadenylation of mRNA
Cysteine formation from homocysteine
Cyclin A/B1 associated events during G2/M transition
CTLA4 inhibitory signaling
CREB phosphorylation through the activation of Ras
COPII (Coat Protein 2) Mediated Vesicle Transport
Conversion from APC/C:Cdc20 to APC/C:Cdh1 in late anaphase
Coenzyme A biosynthesis
Clathrin derived vesicle budding
Class B/2 (Secretin family receptors)
Chromosome Maintenance
Cholesterol biosynthesis
Chk1/Chk2(Cds1) mediated inactivation of Cyclin B:Cdk1 complex
Ceramide signalling
Centrosome maturation
Cell death signalling via NRAGE, NRIF and NADE
Cell Cycle, Mitotic
Cell Cycle Checkpoints
Cell Cycle
CDT1 association with the CDC6:ORC:origin complex
CDC6 association with the ORC:origin complex
c−src mediated regulation of Cx43 function and closure of gap junctions
Biosynthesis of the N−glycan precursor (dolichol lipid−linked oligosaccharide, LLO) and transfer to a nascent protein
Beta oxidation of myristoyl−CoA to lauroyl−CoA
Beta oxidation of lauroyl−CoA to decanoyl−CoA−CoA
Axonal growth stimulation
Axonal growth inhibition (RHOA activation)
Attachment of GPI anchor to uPAR
Assembly of the pre−replicative complex
Assembly of the ORC complex at the origin of replication
Asparagine N−linked glycosylation
Apoptotic execution phase
APC/C:Cdh1 mediated degradation of Cdc20 and other APC/C:Cdh1 targeted proteins in late mitosis/early G1
APC/C:Cdc20 mediated degradation of Cyclin B
APC/C−mediated degradation of cell cycle proteins
APC−Cdc20 mediated degradation of Nek2A
Amyloids
Amplification of signal from the kinetochores
Amplification of signal from unattached kinetochores via a MAD2 inhibitory signal
Amino acid synthesis and interconversion (transamination)
AKT phosphorylates targets in the nucleus
AKT phosphorylates targets in the cytosol
ADP signalling through P2Y purinoceptor 12
Adenylate cyclase inhibitory pathway
Adaptive Immune System
Acyl chain remodelling of PS
Acyl chain remodelling of PE
Acyl chain remodelling of PC
Activation of the pre−replicative complex
Activation of the AP−1 family of transcription factors
Activation of Gene Expression by SREBP (SREBF)
Activation of GABAB receptors
Activation of Chaperones by IRE1alpha
Activation of Chaperone Genes by XBP1(S)
Activation of BAD and translocation to mitochondria
Activation of ATR in response to replication stress
Activated TLR4 signalling
Activated AMPK stimulates fatty−acid oxidation in muscle
Description
1 2 3 4 5 6 7 8 9 10 11 12 13 14 15 16 17 18 19 20
Cluster
qvalue
9
6
3
qvalue
3
6
9

Figure S16 Over-representation analyses for the principal component (PC) of single cells from same cell types in the same cell cycle phases (G1).
Overrepresentation of Gene ontology terms (upper panel) or Reactome pathway database (lower panel) in each PC with 20 single ES (G1 phase). The differently colored and differently sized dots represent –log10(q value) of the hypergeometric test. The X- and Y-axes represent the principal components and the Gene Ontology term or Reactome pathway, respectively.

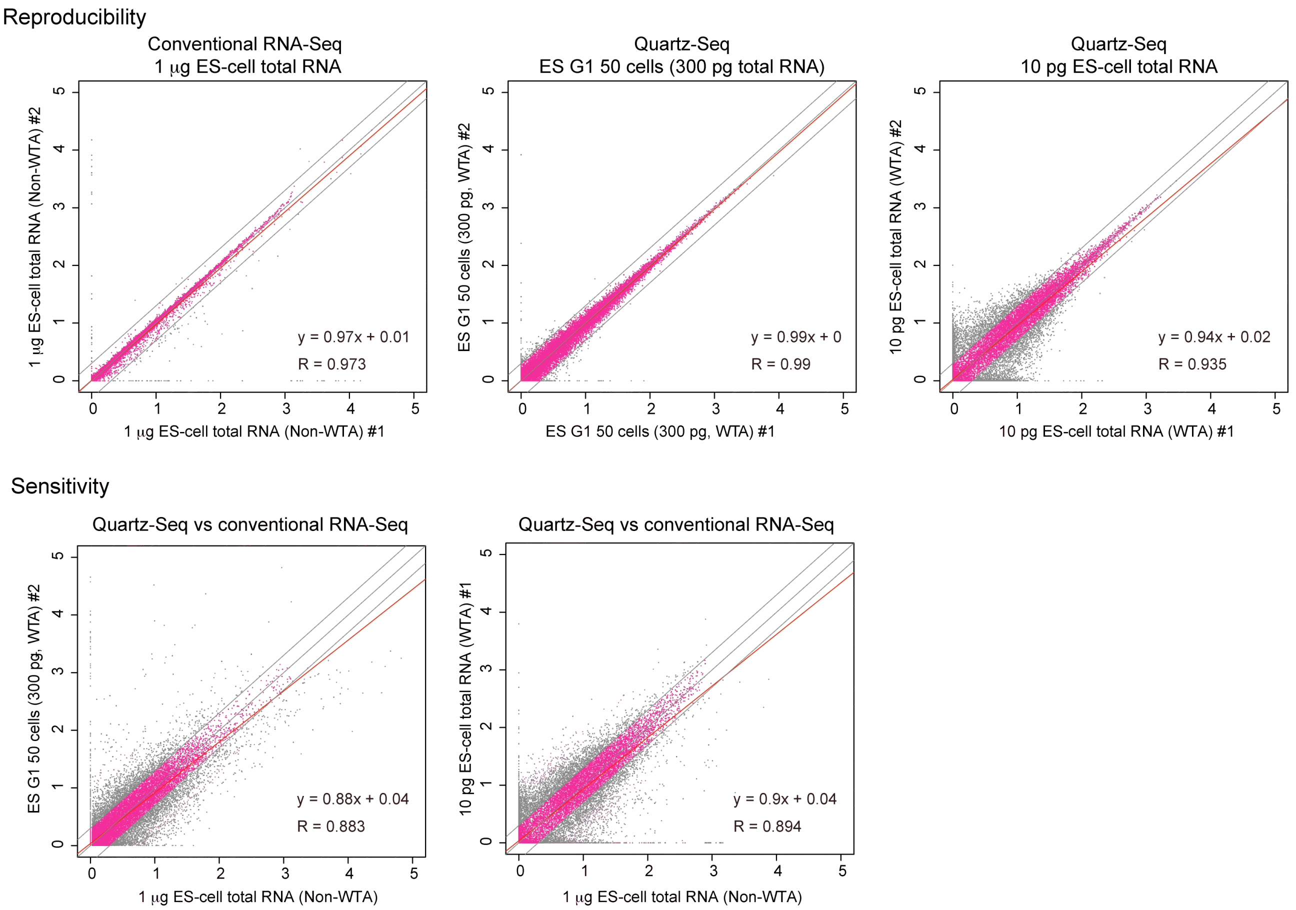

Figure S17 Scatter plots of conventional RNA-Seq and Quartz-Seq using 50 ES cells in the G1 phase of the cell cycle and Quartz-Seq using 10 pg of total ES RNA.

The upper panel shows the reproducibility of technical replicates of conventional RNA-Seq, Quartz-Seq (50 cells) and Quartz-Seq (10 pg of total ES RNA). The lower panel shows the sensitivity of Quartz-Seq with 50 single cells and 10 pg of total ES RNA. The left scatterplot is same in Figure 2. The respective Pearson correlation (R) and regression equation are shown in each plot. The gray lines indicate a two-fold change and y = x. The red line is a linear regression. The red points indicate a transcript with an FPKM expression larger than 1.0 and exhibited less than two-fold expression changes between technical duplicates.

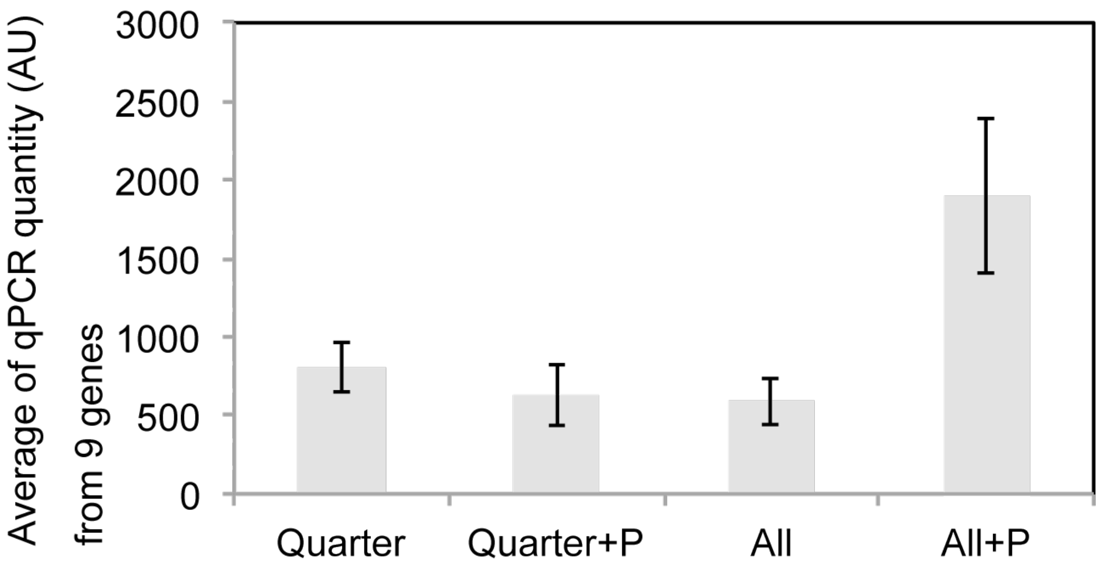
Average of qPCR quantity (AU)
from 9 genes
3000
2500
2000
1500
1000
500
0
Quarter
Quarter+P
All
All+P

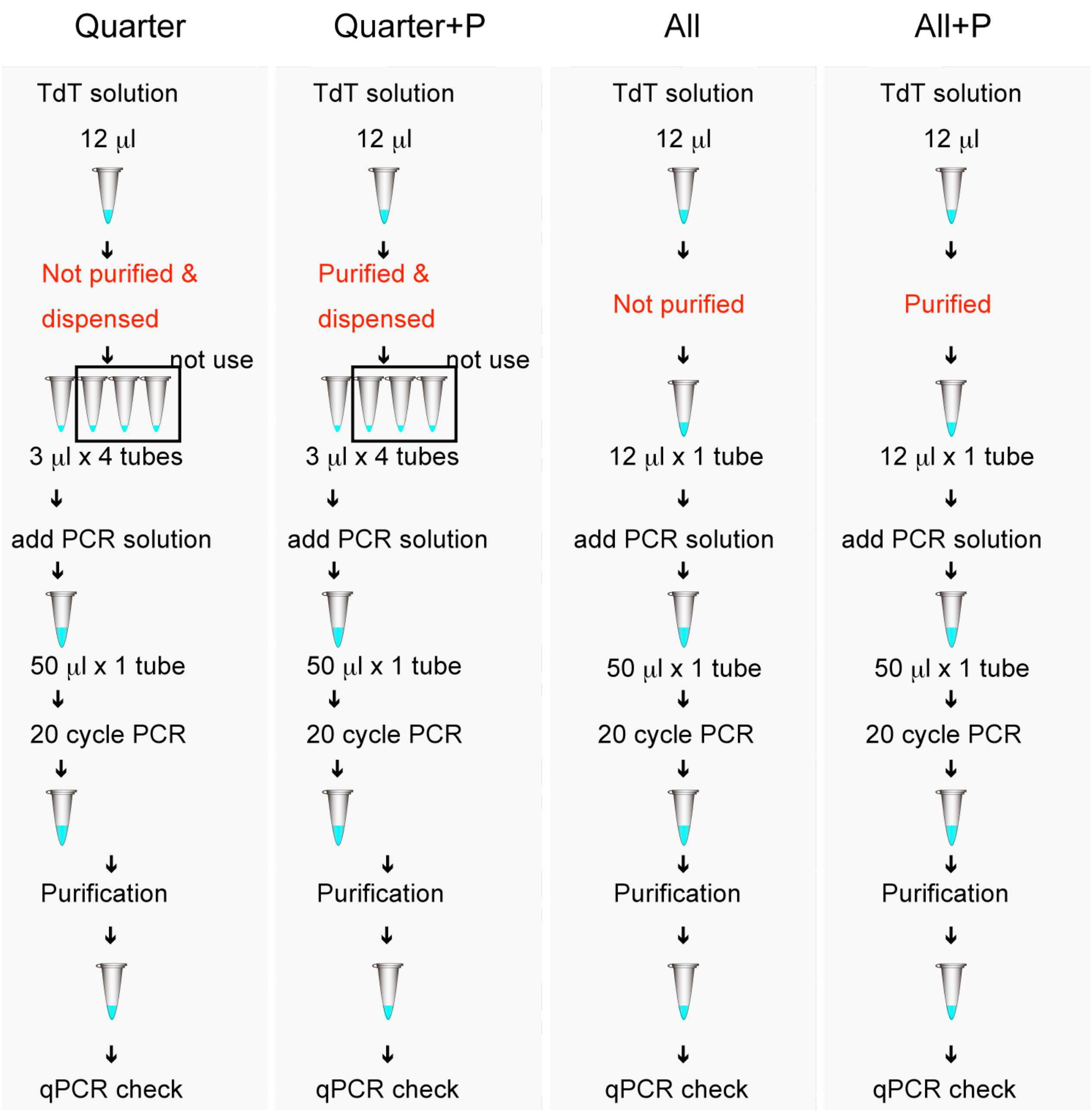
Quarter
Quarter+P
All
All+P
TdT solution
12 μl
Not purified &
dispensed
not use
3 μl x 4 tubes
add PCR solution
50 μl x 1 tube
20 cycle PCR
Purification
qPCR check
TdT solution
12 μl
Purified &
dispensed
not use
3 μl x 4 tubes
add PCR solution
50 μl x 1 tube
20 cycle PCR
Purification
qPCR check
TdT solution
12 μl
Not purified
12 μl x 1 tube
add PCR solution
50 μl x 1 tube
20 cycle PCR
Purification
qPCR check
TdT solution
12 μl
Purified
12 μl x 1 tube
add PCR solution
50 μl x 1 tube
20 cycle PCR
Purification
qPCR check

Figure S18 Effect of carry over buffer on PCR efficiency.
We performed the Kurimoto et al. method with 10 pg of total RNA from ES cells. After the terminal transferase (TdT) reaction, the TdT solution was dispensed into 4 tubes according to the original method. In this experiment, we used one-quarter or all of the TdT solution for the subsequent PCR amplification step with Ex Taq DNA polymerase (TaKaRa). After 20 cycles of PCR amplification, we purified the amplified cDNA with Agencourt Ampure XP beads. Subsequently, we evaluated the gene expression of 10 transcripts by qPCR. The bar plots show the yield of the amplified cDNA using the average of the qPCR quantity of the 10 genes for each condition (n=5). The detected genes were the following: *Trim28*, *Ywhae*, *Sox2*, *Prdx1, Nanog, Dppa5, Pou5f1, Lefty1, Dnmt1* and *Ifitm3*. One-quarter or all of the TdT solution was purified using Agencourt AmpureXP beads in the “Quarter+P” and “All+P” samples, respectively. When all of the TdT solution was used for the PCR amplification in single PCR tube, the PCR efficiency was disrupted by the presence of carry-over buffer.
