# Supplementary material for: Quartz-Seq: a highly reproducible and sensitive single-cell RNA sequencing method, reveals non-genetic gene-expression heterogeneity
Source: Genome Biol. 2013 Apr 17;14(4):R31. doi: 10.1186/gb-2013-14-4-r31 (PMC4054835; doi:10.1186/gb-2013-14-4-r31)
Supplement: Additional file 2 — Supplementary note. [file gb-2013-14-4-r31-S2.DOCX]

# Supplementary Notes

**Detailed technical development of whole-transcript amplification for Quartz-Seq**

**PCR enzyme selection**

The use of a purification-free single-tube reaction improved the sensitivity of the ChIP amplification method [[1](#_ENREF_1)]. We also adopted this approach in our method. In this approach, the buffer used in the previous steps is carried over into the following steps. In general, carry-over (buffer, proteins, and so on) prevents PCR amplification. In fact, we confirmed that approximately 24 % of the carry-over for PCR amplification obviously reduces the cDNA yield (see Additional file 1, Figure S18). To avoid the carry-over effect, we designed our method to reduce the total volume of carry-over for the subsequent PCR reaction. Moreover, we tested two PCR enzymes (KOD Fx [Toyobo] and MightyAmp [TaKaRa-Clontech]) that are robust for crude sample and GC-rich sequences. MightyAmp DNA polymerase was able to achieve high sensitivity and reproducibility in a single-tube PCR amplification (see Additional file 1, Figure S5 and Figure S11). We designed the Quartz-Seq method to ensure that the length of almost all the resulting cDNA was less than approximately 3,000 base pairs (see Additional file 1, Figure S10). After a 21-cycle PCR enrichment of 10 pg of total RNA, we obtained approximately 63 ng of amplified cDNA. This cDNA amount is sufficient to perform all of the following applications: sequencing; microarray analysis; and quality checks, such as the BioAnalyzer, PicoGreen, and qPCR assays.

**Library preparation for Quartz-Seq**

We used aminated PCR suppression primers for WTA, and the contamination rate from the WTA adaptor regions increased with the use of non-aminated primers (13.27 ± 0.13 %).

**Evaluation of Quartz-Seq with an unpurified single-cell-sized sample**

We demonstrated the results of single-cell Quartz-Seq with 10 pg of purified total RNA, which does not contain crude cell lysate. We should also evaluate single-cell Quartz-Seq using a single-cell-sized sample containing crude cell lysate. We performed single-cell Quartz-Seq with unpurified single-cell-sized samples of pooled cells. We present all of the evaluation data (e.g., Pearson correlation coefficient and the number of detected transcripts) in Supplementary table 1 (with an isolated single-cell sheet and a single-cell-level sample). We prepared an unpurified single-cell-sized sample for Quartz-Seq. We used ES and PrE cells in G1 phase, which contained approximately 6 pg of total RNA per cell (see Additional file 1, Figure S14). Twelve single cells in G1 phase were dissolved in a 12-fold volume of lysis buffer. Subsequently, a single-cell-sized lysis sample was dispensed into single PCR tube. Quartz-Seq was capable of reproducibly detecting 6,977 ± 122.9 transcripts in a mouse ES single-cell-sized sample in G1 phase (n = 3) and 6,996.4 ± 40.8 transcripts in a mouse PrE single-cell-sized sample in G1 phase (n = 6). The Pearson correlation coefficient (PCC) between pairs of samples with a single-cell-sized sample was as follows: ES vs. ES, 0.85 ± 0.004; PrE vs. PrE, 0.852 ± 0.003; and ES vs. PrE, 0.782 ± 0.003. The PCC between pairs of samples using 10 pg of purified total RNA was 0.93 ± 0.002. The PCC of Quartz-Seq with only 6 pg of a single-cell-sized sample was sufficiently high to reveal non-genetic gene expression heterogeneity.

**The PCCs and numbers of detected transcripts of single-cell RNA-seq data with isolated single cells**

We also presented the variability of isolated single cells. Quartz-Seq was capable of reproducibly detecting 5,758.8 ± 524.2 transcripts in mouse ES single cells in G1 phase (n = 12) and 6,298.8 ± 489.6 transcripts in mouse PrE single cells in G1 phase (n = 12). The PCC between pairs of real single-cells was as follows: ES vs. ES, 0.79 ± 0.01; PrE vs. PrE, 0.807 ± 0.02; and ES vs. PrE, 0.729 ± 0.01. The PCC between pairs of single-cell Smart-Seq data with T24 cells has been reported to be approximately 0.74 [[2](#_ENREF_2)]. T24 cells contained approximately 20 pg of total RNA per cell. Our single-cell Quartz-Seq worked well for real single cells.

We reanalyzed the CEL-Seq data with a mouse ES cell. We counted the number of highly reproducible expressed transcripts that were larger than 1.0 in tpm using those data. CEL-Seq detected 4,070.3 ± 332.4 transcripts in mouse ES single-cells (n = 9). However, Quartz-Seq detected 6,069.1 ± 854.9 transcripts in mouse ES single-cells in all cell-cycle phases (n = 35). The PCC of Quartz-Seq was 0.804 ± 0.02, whereas the PCC of CEL-Seq was 0.681 ± 0.03. These results also suggested that single-cell Quartz-Seq has a better quantitative performance (reproducibility and sensitivity) for real single cells.

1. Shankaranarayanan P, Mendoza-Parra MA, Walia M, Wang L, Li N, Trindade LM, Gronemeyer H: **Single-tube linear DNA amplification (LinDA) for robust ChIP-seq.** *Nat Methods* 2011, **8:**565-567.

2. Ramskold D, Luo S, Wang YC, Li R, Deng Q, Faridani OR, Daniels GA, Khrebtukova I, Loring JF, Laurent LC, et al: **Full-length mRNA-Seq from single-cell levels of RNA and individual circulating tumor cells.** *Nat Biotechnol* 2012.
