# Supplementary figures and images for: Quartz-Seq: a highly reproducible and sensitive single-cell RNA sequencing method, reveals non-genetic gene-expression heterogeneity
Source: Genome Biol. 2013 Apr 17;14(4):R31. doi: 10.1186/gb-2013-14-4-r31 (PMC4054835; doi:10.1186/gb-2013-14-4-r31)

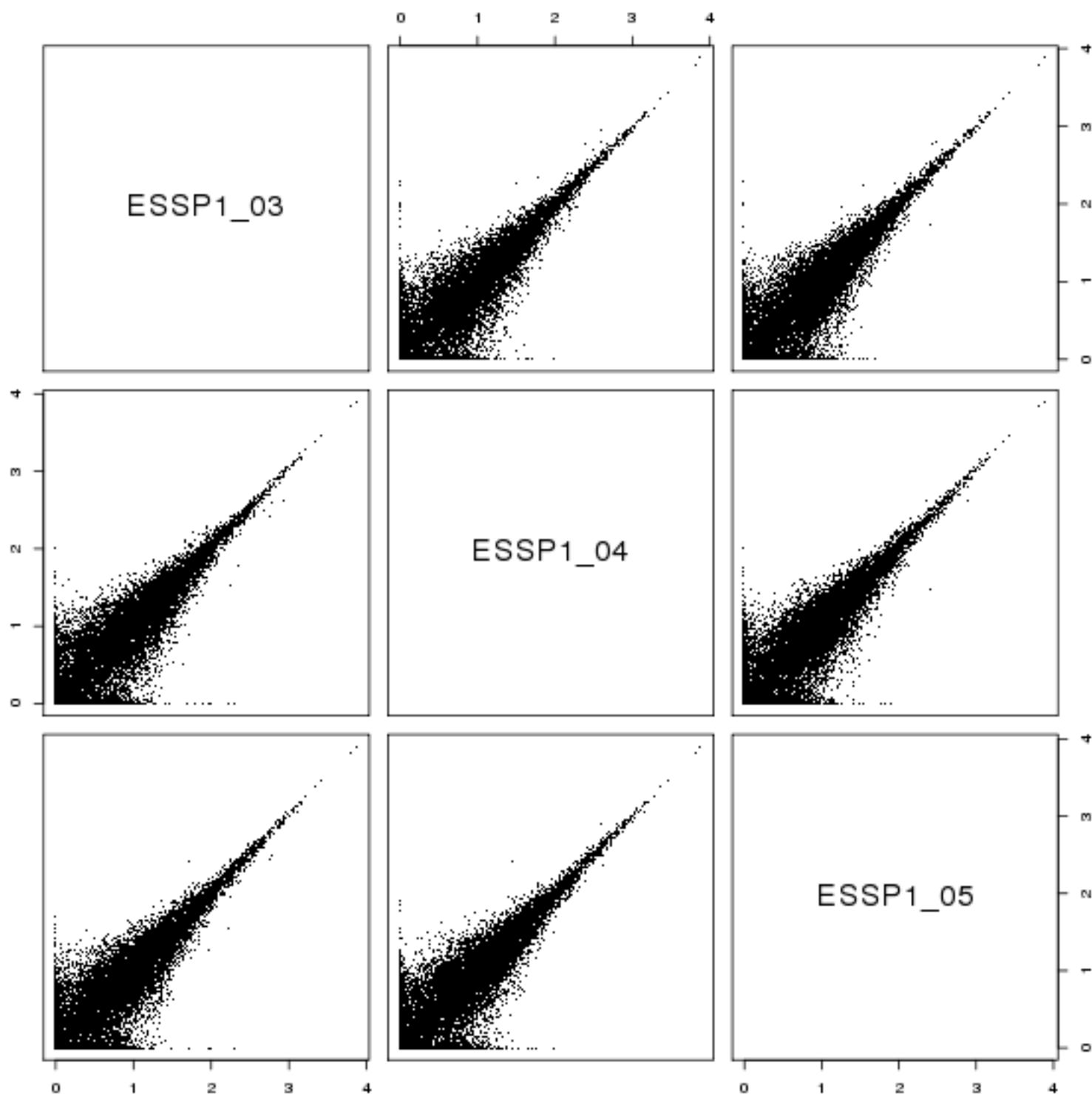

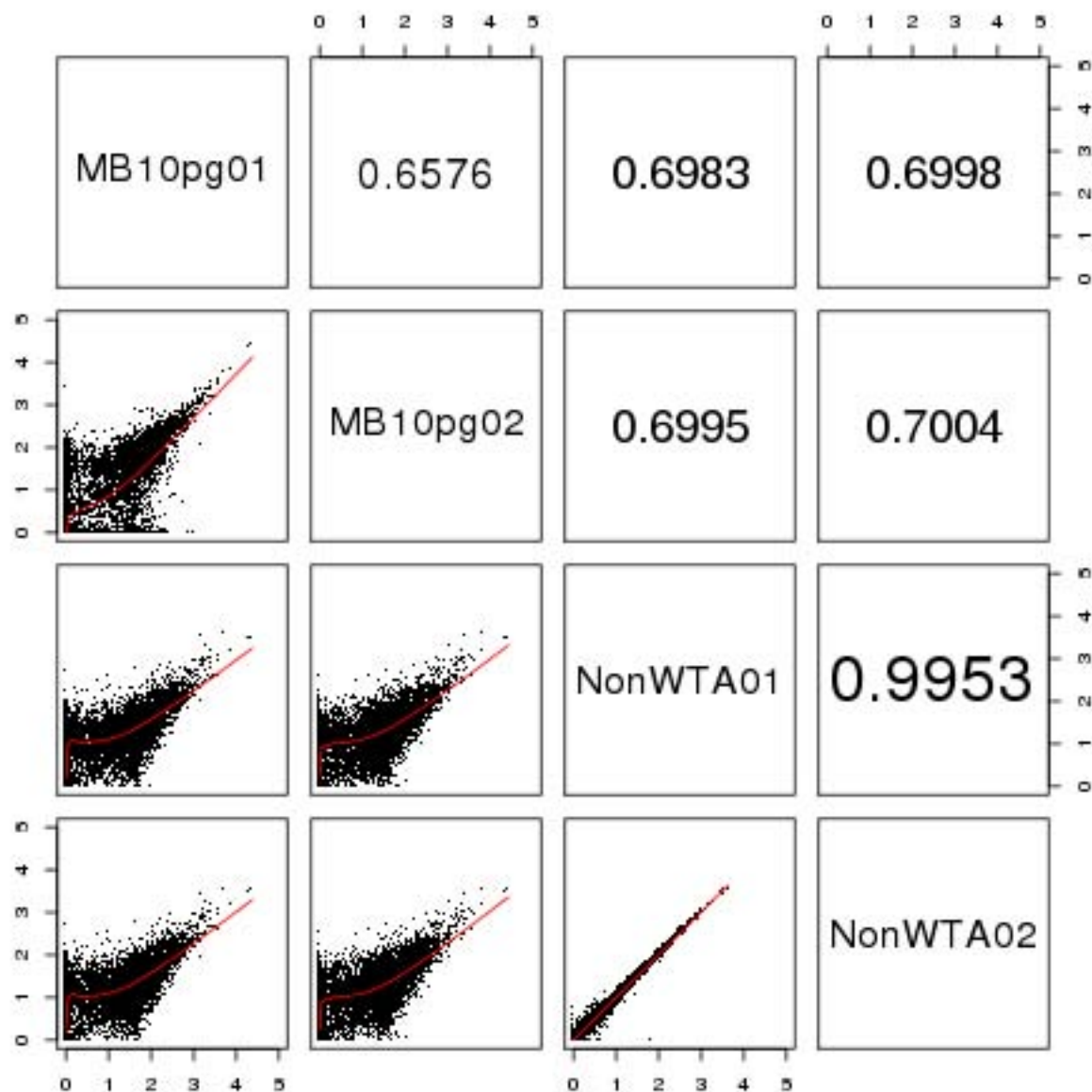

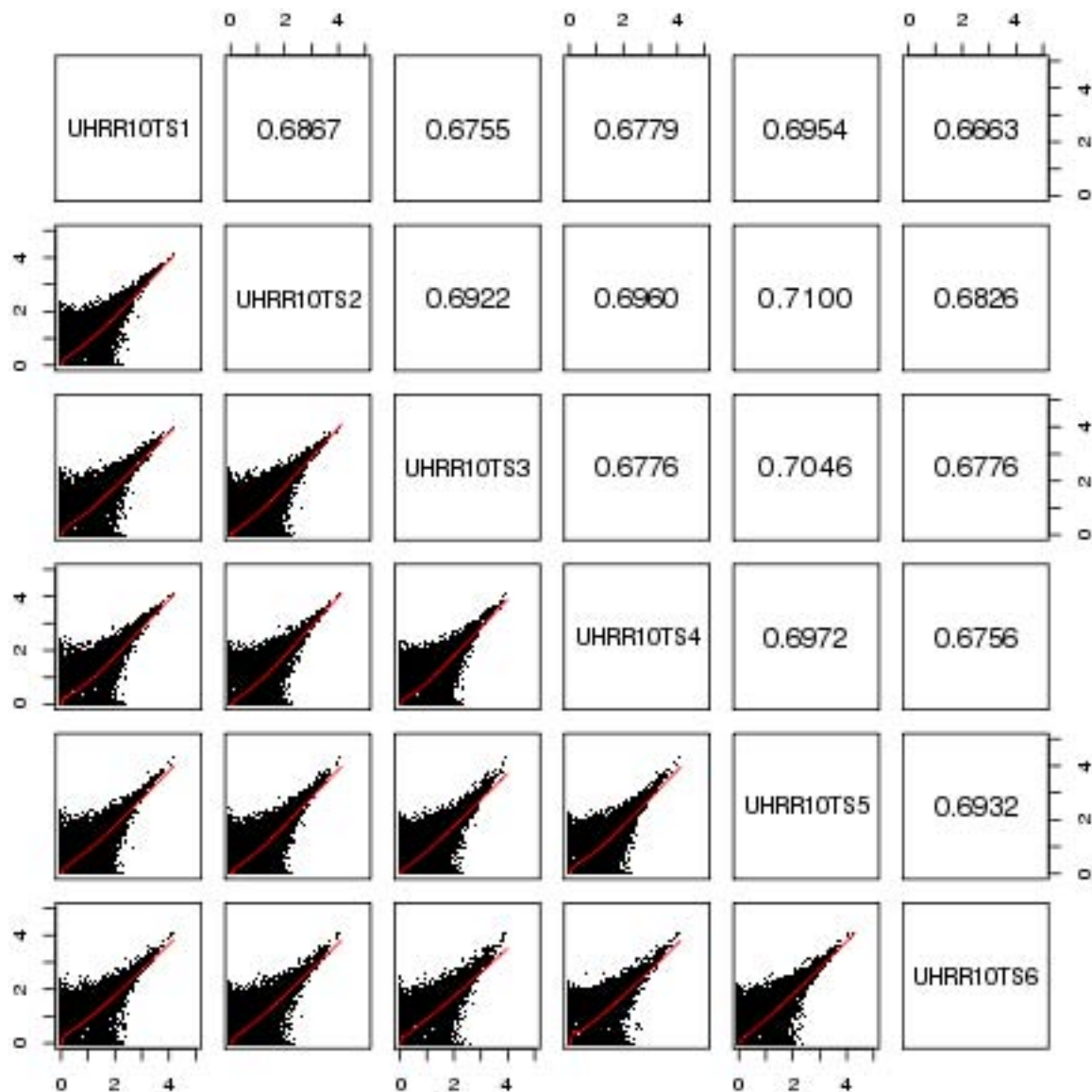

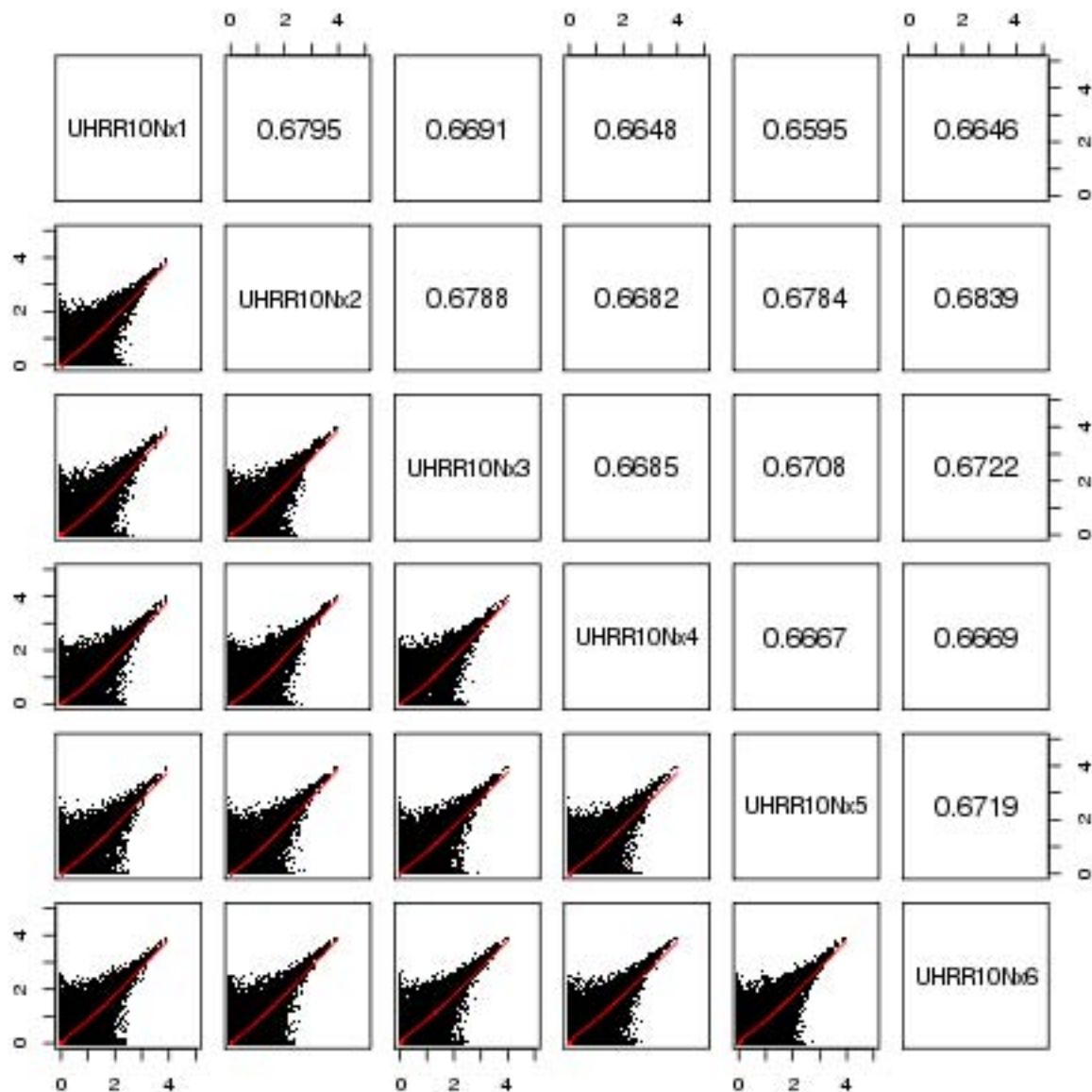

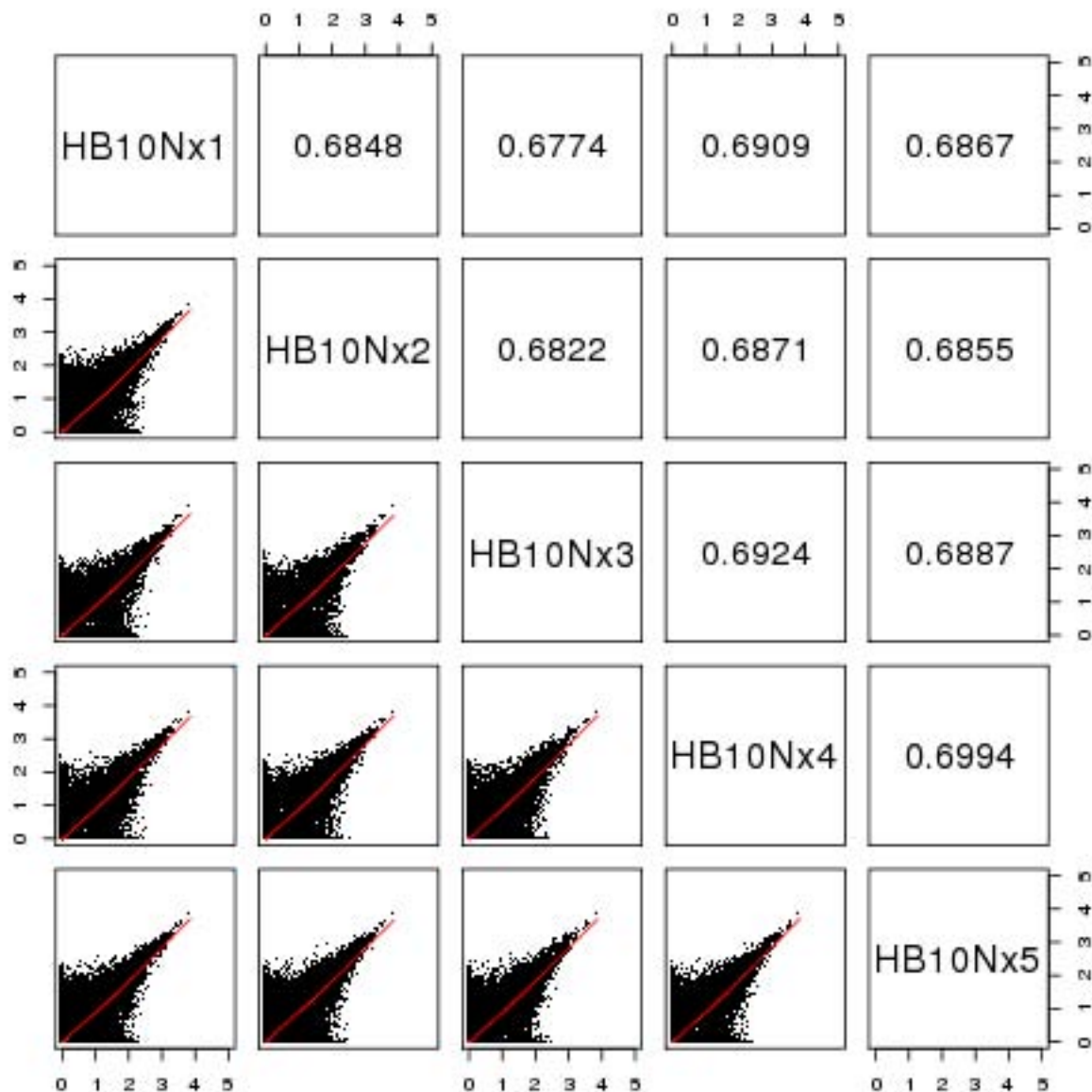

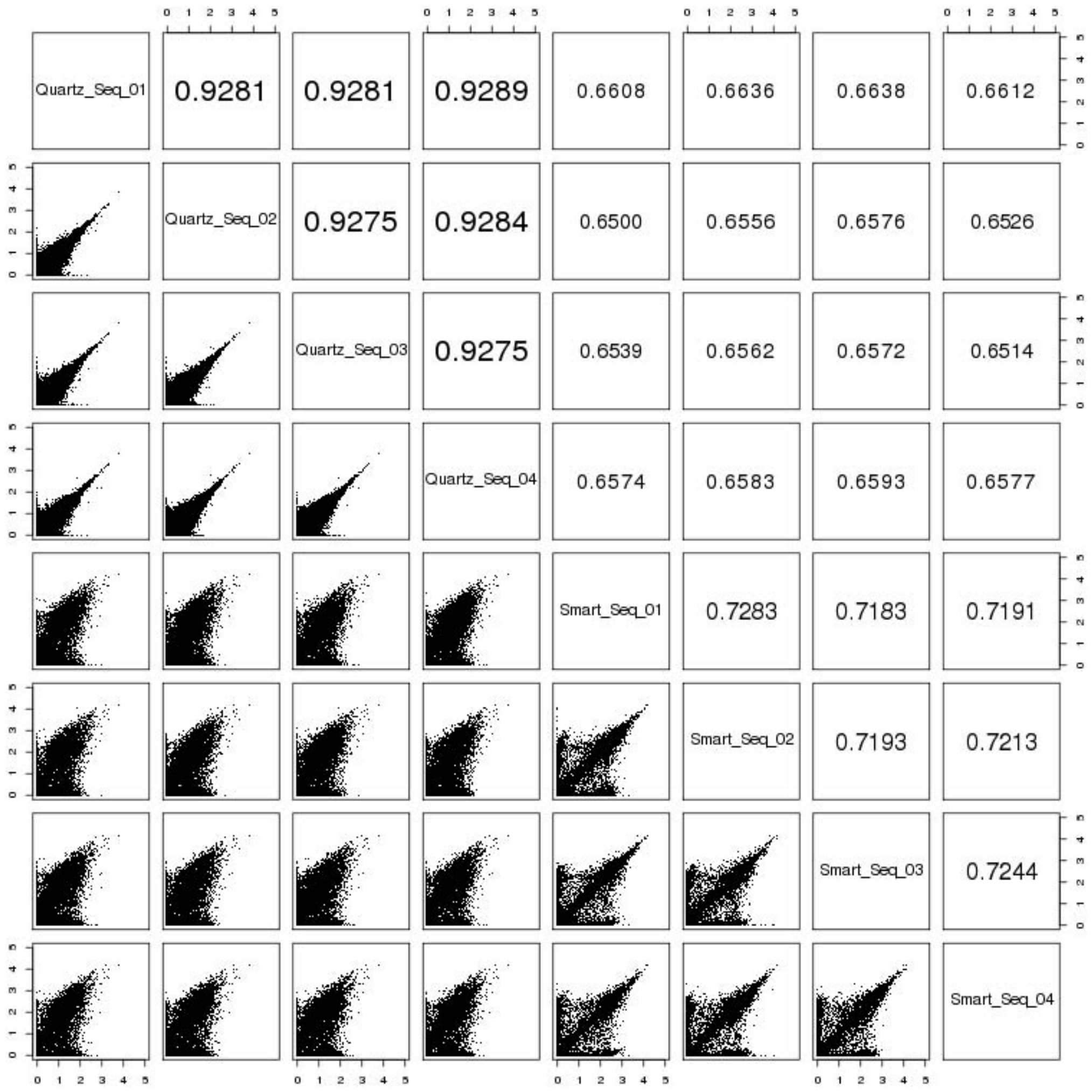

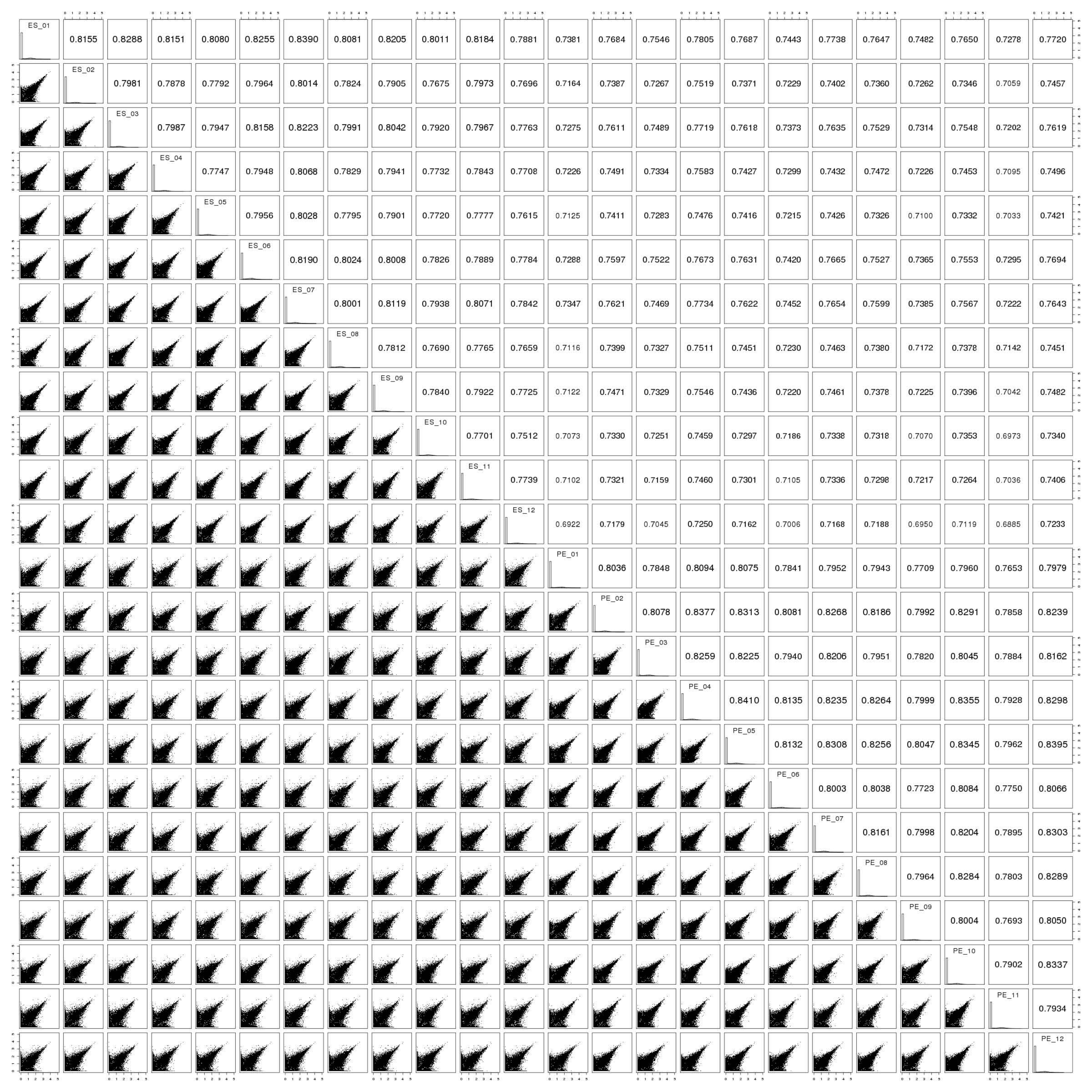

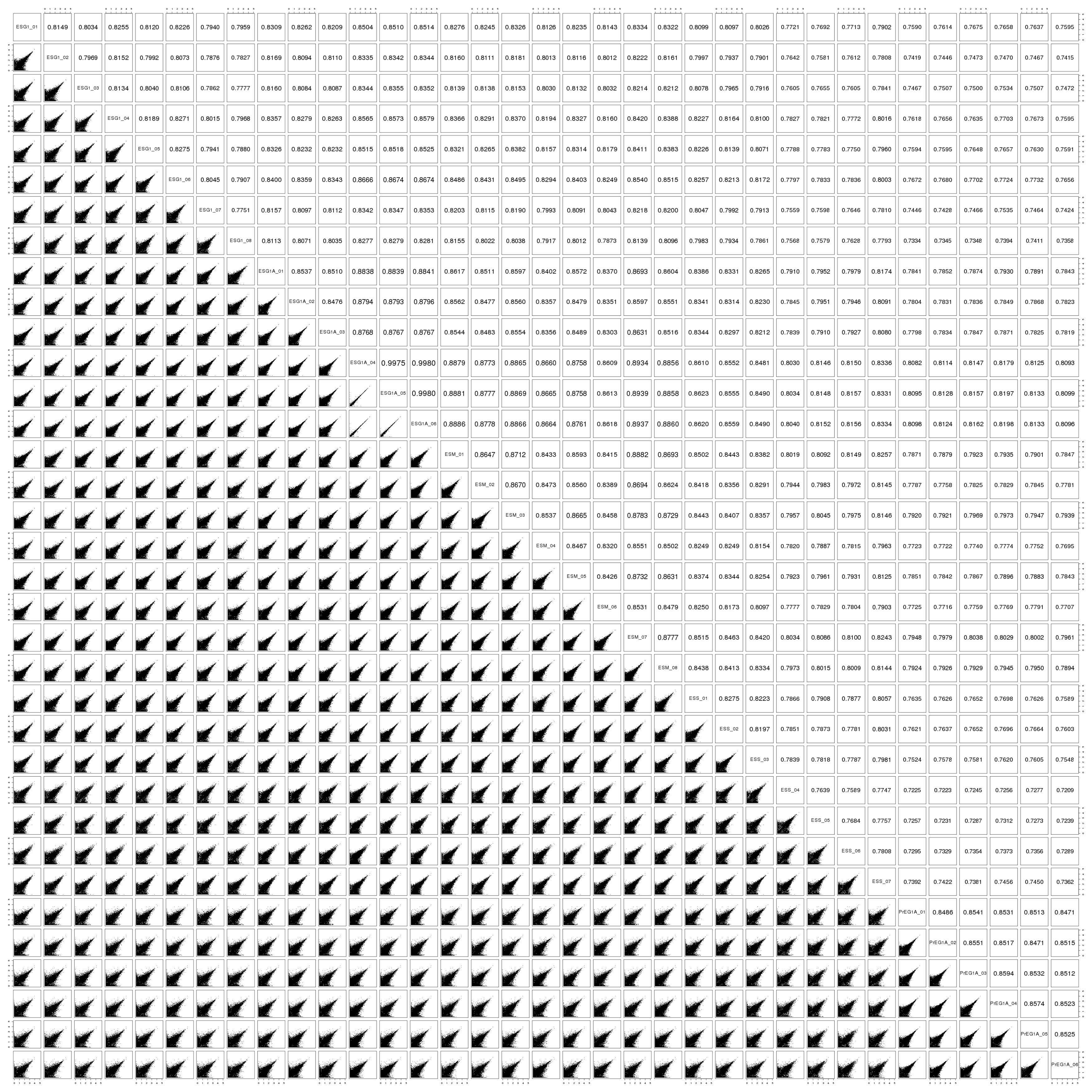

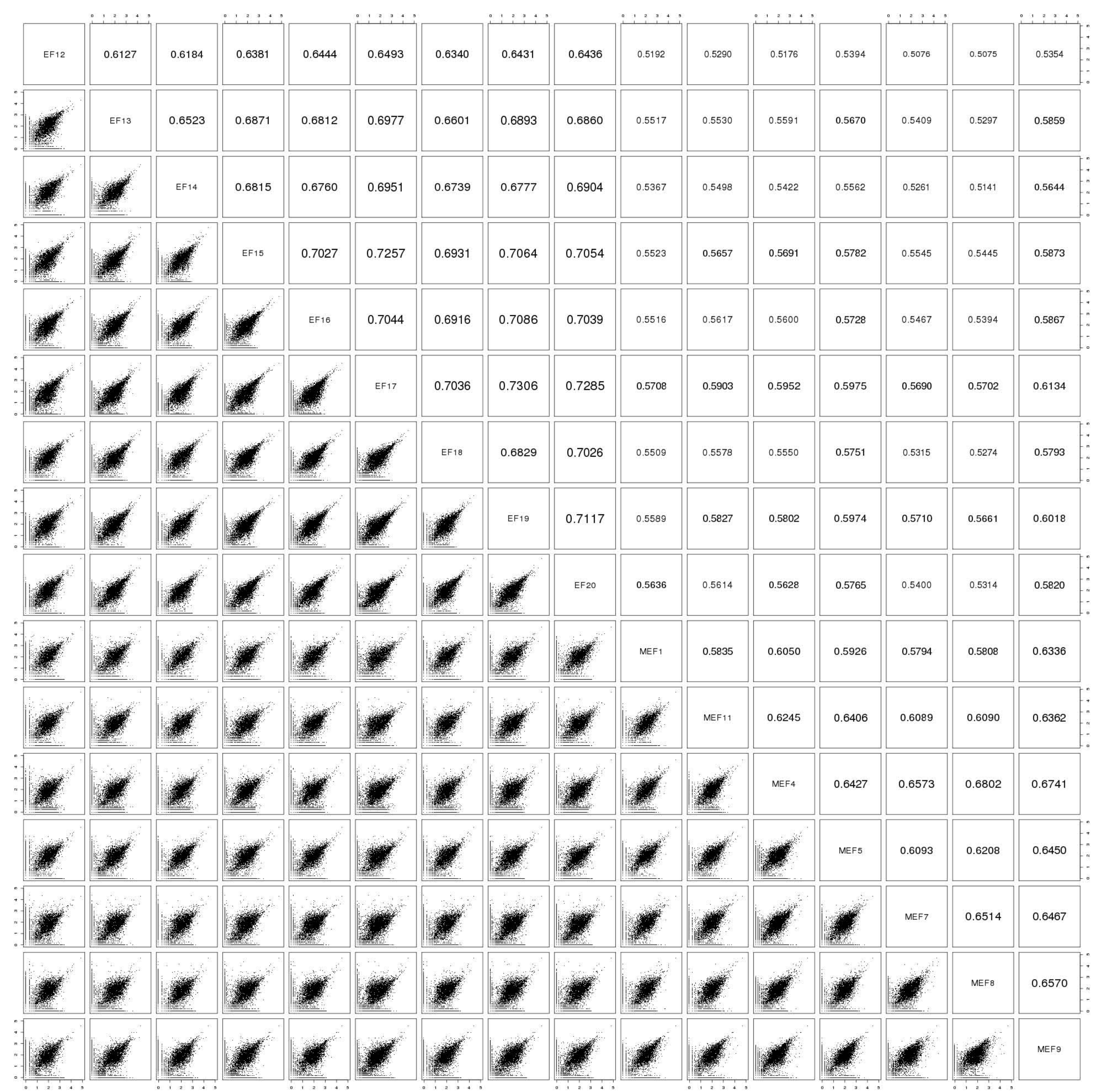

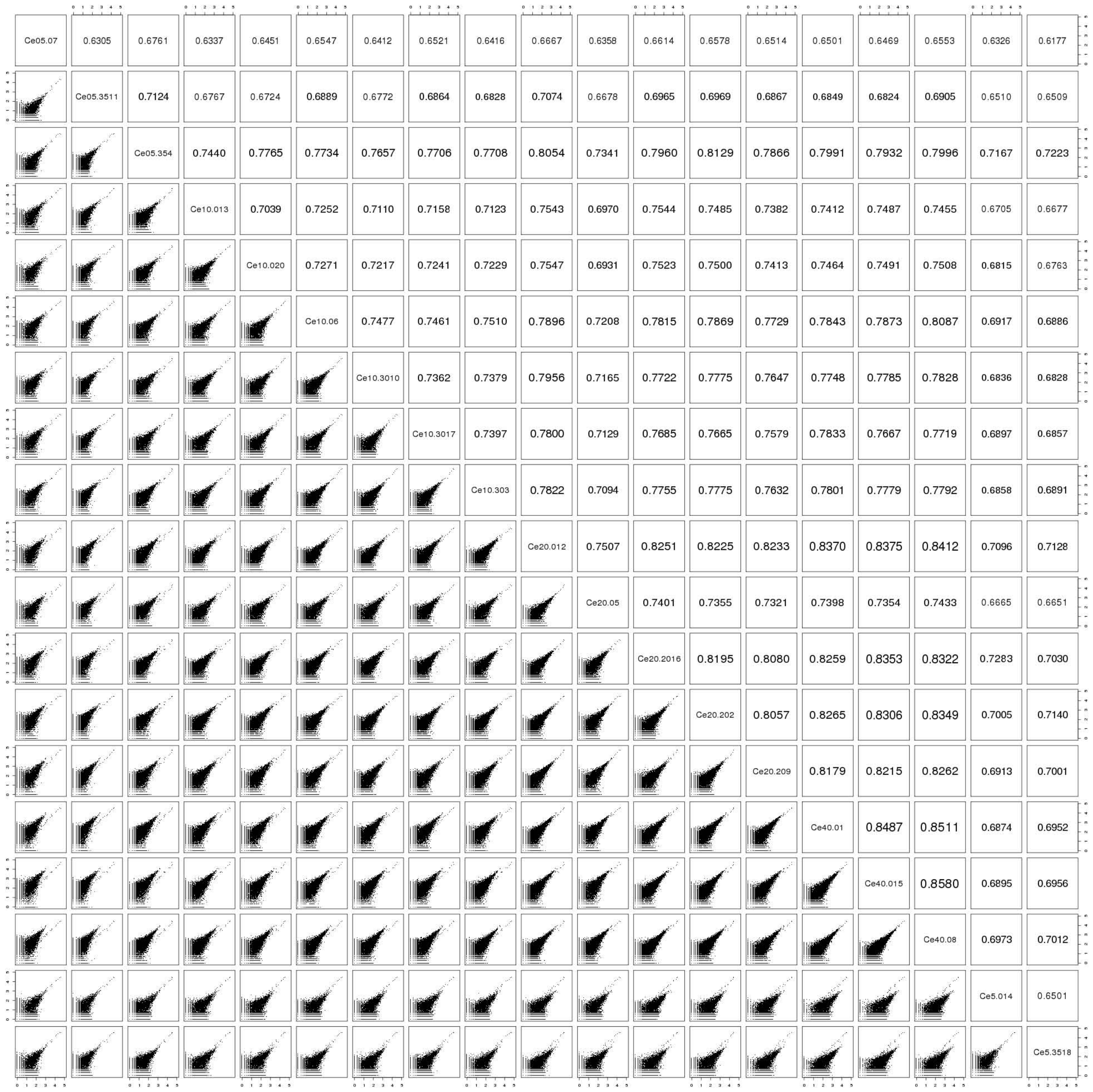

Supplement: Additional file 3 — Figure S7: All scatter plots [file gb-2013-14-4-r31-S3.PDF]

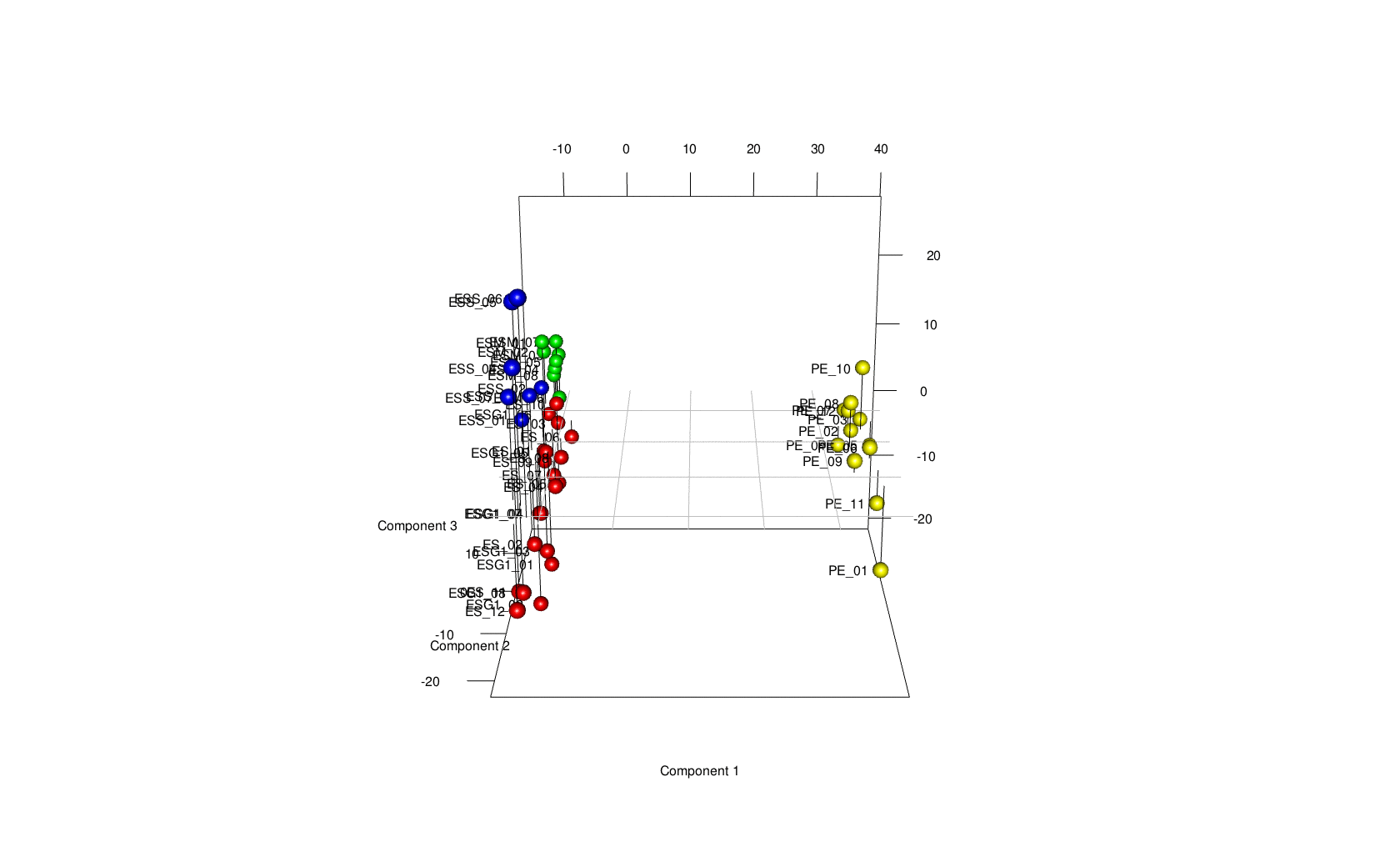

Supplement: Additional file 5 — Supplementary movie 1. Principal component analysis (PCA) with single-cell Quartz-Seq data of embryonic stem (ES) and primitive endoderm (PrE) single-cell preparations. [file gb-2013-14-4-r31-S5.GIF]

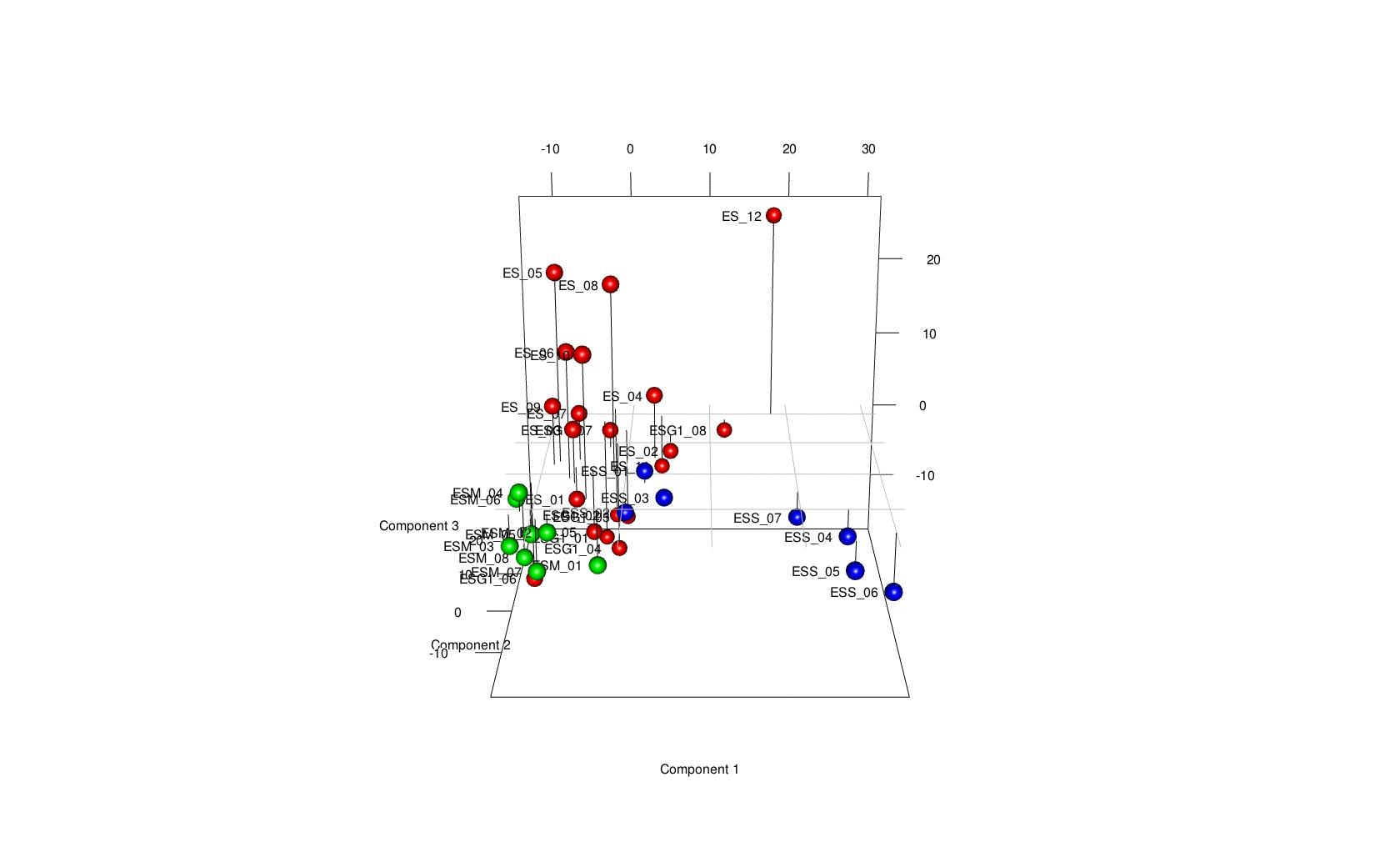

Supplement: Additional file 6 — Supplementary movie 2. Principal component analysis (PCA) with single-cell Quartz-Seq data of embryonic stem (ES) cells in different cell-cycle phases. [file gb-2013-14-4-r31-S6.GIF]
